# Bi-allelic pathogenic variants in *TRMT1* disrupt tRNA modification and induce a neurodevelopmental disorder

**DOI:** 10.1016/j.ajhg.2025.03.015

**Published:** 2025-04-16

**Authors:** Stephanie Efthymiou, Cailyn P. Leo, Chenghong Deng, Sheng-Jia Lin, Reza Maroofian, Renee Lin, Irem Karagoz, Kejia Zhang, Rauan Kaiyrzhanov, Annarita Scardamaglia, Daniel Owrang, Valentina Turchetti, Friederike Jahnke, Kevin Huang, Cassidy Petree, Anna V. Derrick, Mark I. Rees, Javeria Raza Alvi, Tipu Sultan, Chumei Li, Marie-Line Jacquemont, Frederic Tran-Mau-Them, Maria Valenzuela-Palafoll, Rich Sidlow, Grace Yoon, Michelle M. Morrow, Deanna Alexis Carere, Mary O'Connor, Julie Fleischer, Erica H. Gerkes, Chanika Phornphutkul, Bertrand Isidor, Clotilde Rivier-Ringenbach, Christophe Philippe, Semra Hiz Kurul, Didem Soydemir, Bulent Kara, Deniz Sunnetci-Akkoyunlu, Viktoria Bothe, Konrad Platzer, Dagmar Wieczorek, Margarete Koch-Hogrebe, Nils Rahner, Ann-Charlotte Thuresson, Hans Matsson, Carina Frykholm, Sevcan Tuğ Bozdoğan, Atil Bisgin, Nicolas Chatron, Gaetan Lesca, Sara Cabet, Zeynep Tümer, Tina D. Hjortshøj, Gitte Rønde, Thorsten Marquardt, Janine Reunert, Erum Afzal, Mina Zamani, Reza Azizimalamiri, Hamid Galehdari, Pardis Nourbakhsh, Niloofar Chamanrou, Seo-Kyung Chung, Mohnish Suri, Paul J. Benke, Maha S. Zaki, Joseph G. Gleeson, Daniel G. Calame, Davut Pehlivan, Halil I. Yilmaz, Alper Gezdirici, Aboulfazl Rad, Iman Sabri Abumansour, Gabriela Oprea, Muhammed Burak Bereketoğlu, Guillaume Banneau, Sophie Julia, Jawaher Zeighami, Saeed Ashoori, Gholamreza Shariati, Alireza Sedaghat, Alihossein Sabri, Mohammad Hamid, Sahere Parvas, Tajul Arifin Tajudin, Uzma Abdullah, Shahid Mahmood Baig, Wendy K. Chung, Olga O. Glazunova, Sigaudy Sabine, Huma Arshad Cheema, Giovanni Zifarelli, Peter Bauer, Jai Sidpra, Kshitij Mankad, Barbara Vona, Andrew E. Fry, Gaurav K. Varshney, Henry Houlden, Dragony Fu

**Affiliations:** 1 Department of Neuromuscular disorders, UCL Queen Square Institute of Neurology, London WC1N 3BG, UK; 2 Department of Biology, Center for RNA Biology, University of Rochester, Rochester, NY, USA; 3 Genes & Human Disease Research Program, Oklahoma Medical Research Foundation, Oklahoma City, OK 73104, USA; 4 Institute for Auditory Neuroscience and Inner Ear Lab, University Medical Center Göttingen, Robert-Koch-Str. 40, 37075 Göttingen, Germany; 5 Institute of Human Genetics, University Medical Center Göttingen, Heinrich-Düker-Weg 12, 37073 Göttingen, Germany; 6 Neurology Research Group, Institute of Life Science, Swansea University Medical School, Swansea University, Swansea SA2 8PP, UK; 7 Faculty of Medicine & Health, Camperdown, University of Sydney, Sydney, NSW, Australia; 8 Department of Pediatric Neurology, Institute of Child Health, Children’s Hospital, Lahore 54590, Pakistan; 9 McMaster University, 1280 Main St W, Hamilton, ON L8S 4L8, Canada; 10 Unité de Génétique Médicale et Centre de Référence Anomalies du Développement et Syndromes Malformatifs, CHU de la Réunion, Saint-Pierre, France; 11 Unité Fonctionnelle Innovation en Diagnostic Génomique des maladies rares, CHU Dijon Bourgogne, Dijon, France; 12 INSERM UMR1231 GAD, F-21000 Dijon, France; 13 Department of Clinical and Molecular Genetics, Vall d’Hebron University Hospital and Medicine Genetics Group, Vall d’Hebron Research Institute, Barcelona, Spain; 14 Department of Medical Genetics and Metabolism, Valley Children’s Hospital, Madera, CA, USA; 15 Hospital for Sick Children, Toronto, ON, Canada; 16 University of Toronto, Toronto, ON, Canada; 17 GeneDx, LLC, Gaithersburg, MD 20877, USA; 18 Department of Pediatrics, Southern Illinois University School of Medicine, Springfield, IL, USA; 19 Department of Medical Genetics, University of Groningen and University Medical Center Groningen, Department of Genetics, Groningen, the Netherlands; 20 Division of Human Genetics, Department of Pediatrics, Warren Alpert Medical School of Brown University, Hasbro Children’s Hospital, Providence, RI, USA; 21 Centre Hospitalier Universitaire de Nantes, Service de Génétique Médicale, Nantes, France; 22 INSERM, CNRS, UNIV Nantes, L’institut du Thorax, Nantes, France; 23 Hôpital Nord-Ouest, Service de Neuropédiatrie, Villefranche sur Saône, France; 24 Laboratoire de Génétique, Hôpital Mercy, CHR Metz-Thionville, Metz, France; 25 Department of Pediatric Neurology, Faculty of Medicine, Dokuz Eylül University, İzmir, Turkey; 26 İzmir Biomedicine and Genome Center, Dokuz Eylül University Health Campus, İzmir, Turkey; 27 İzmir International Biomedicine and Genome Institute, Dokuz Eylül University, İzmir, Turkey; 28 Division of Pediatric Neurology, Department of Pediatrics, Kocaeli University, Kocaeli, Turkey; 29 Department of Medical Genetics, Faculty of Medicine, Kocaeli University, Kocaeli, Turkey; 30 Institute of Human Genetics, University of Leipzig Medical Center, Leipzig, Germany; 31 Institute of Human Genetics, Medical Faculty and University Hospital Düsseldorf, Heinrich-Heine-University Düsseldorf, Düsseldorf, Germany; 32 Vestische Kinder- und Jugendklinik Datteln, Abteilung für Neuropädiatrie, Datteln, Germany; 33 MVZ Institute for Clinical Genetics and Tumor Genetics, Bonn, Germany; 34 Department of Immunology, Genetics and Pathology, Uppsala University, 751 85 Uppsala, Sweden; 35 Cukurova University AGENTEM (Adana Genetic Diseases Diagnosis and Treatment Center), Adana, Turkey; 36 VariantGen Genetic Diagnosis, Treatment, and Healthcare Center, Adana, Turkey; 37 Hospices Civils de Lyon, Service de Génétique, Centre Labélisé Anomalies du Développement CLAD Sud-Est, Lyon, France; 38 Institut Neuromyogène, Laboratoire Physiopathologie et Génétique du Neurone et du Muscle, Equipe Métabolisme énergétique et développement neuronal, CNRS UMR 5310, INSERM U1217, Université Lyon 1, Lyon, France; 39 Pediatric, Woman and Fetal Imaging Department, Hôpital Femme-Mère-Enfant, Hospices Civils de Lyon, 69500 Bron, France; 40 Institut NeuroMyoGène, CNRS UMR5292, INSERM U1028, Claude Bernard Lyon 1 University, 69000 Lyon, France; 41 Kennedy Center, Department of Clinical Genetics, Copenhagen University Hospital-Rigshospitalet, Copenhagen, Denmark; 42 Department of Clinical Medicine, Faculty of Health and Medical Sciences, University of Copenhagen, Copenhagen, Denmark; 43 Department of Paediatrics and Adolescent Medicine, University Hospital Herlev, Herlev, Denmark; 44 Department of Paediatrics, Metabolic Diseases, University of Münster, Albert-Schweitzer-Campus 1, 48149 Münster, Germany; 45 Department of Developmental and Behavioral Pediatrics, Children’s Hospital and Institute of Child Health, Multan, Punjab 60000, Pakistan; 46 Department of Biology, Faculty of Science, Shahid Chamran University of Ahvaz, Ahvaz, Iran; 47 Narges Medical Genetics and Prenatal Diagnosis Laboratory, Kianpars, Ahvaz, Iran; 48 Department of Pediatric Neurology, Golestan Medical, Educational, and Research Center, Ahvaz Jundishapur University of Medical Sciences, Ahvaz, Iran; 49 Department of Neurology, School of Medicine, Ahvaz Jundishapur University of Medical Sciences, Ahvaz, Iran; 50 Brain & Mind Centre, Faculty of Medicine & Health, Camperdown, University of Sydney, Sydney, NSW, Australia; 51 Kids Research, Children’s Hospital at Westmead, Sydney, NSW, Australia; 52 Nottingham Clinical Genetics Service, Nottingham University Hospitals NHS Trust, City Hospital Campus, Nottingham, NG5 1PB, UK; 53 Department of Clinical Genetics, Joe DiMaggio Children's Hospital, Hollywood, FL 33021, USA; 54 Clinical Genetics Department, Human Genetics and Genome Research Division, Centre of Excellence of Human Genetics, National Research Centre, Cairo, Egypt; 55 Department of Neuroscience, Rady Children’s Institute for Genomic Medicine, University of California, San Diego, San Diego, CA, USA; 56 Division of Pediatric Neurology and Developmental Neuroscience, Department of Pediatrics, Baylor College of Medicine, Houston, TX, USA; 57 Texas Children’s Hospital, Houston, TX, USA; 58 Department of Molecular and Human Genetics, Baylor College of Medicine, Houston, TX, USA; 59 Department of Medical Genetics, Basaksehir Cam and Sakura City Hospital, Istanbul, Turkey; 60 Arcensus GmbH, Rostock, Germany; 61 Neurogenetic Section, Department of Pediatrics, King Faisal Specialist Hospital and Research Center, Jeddah, Saudi Arabia; 62 Department of Medical Genetics, Faculty of Medicine, Umm Al-Qura University, Makkah, Saudi Arabia; 63 Department of Pediatrics, International Medical Center, Jeddah, Saudi Arabia; 64 Ege University Hospital, Department of Medical Genetics, İzmir 35100, Turkey; 65 Department of Clinical Genetics, CHU Toulouse, Toulouse, France; 66 Narges Medical Genetics and Prenatal Diagnosis Laboratory, Kianpars, Ahvaz, Iran; 67 Department of Dermatology, School of Medicine, Jundishapur University of Medical Sciences, Ahvaz, Iran; 68 Department of Medical Genetics, Faculty of Medicine, Ahvaz Jundishapur University of Medical Sciences, Ahvaz, Iran; 69 Health Research Institute, Diabetes Research Center, Jundishapur University of Medical Sciences, Ahvaz, Iran; 70 Department of Molecular Medicine, Biotechnology Research Center, Pasteur Institute of Iran, Tehran, Iran; 71 KPJ Puteri Specialist Hospital, Hospital Sultan Ismail Johor, Johor Bahru, Malaysia; 72 University Institute of Biochemistry and Biotechnology, Pir Mehr Ali Shah Arid Agriculture University, Rawalpindi 46301, Pakistan; 73 National Institute for Biotechnology and Genetic Engineering College (NIBGE-C), Faisalabad, Pakistan Institute of Engineering and Applied Sciences (PIEAS), Islamabad, Pakistan; 74 Department of Pediatrics, Boston Children’s Hospital and Harvard Medical School, Boston, MA, USA; 75 IHU Méditerranée Infection, 19–21 boulevard Jean Moulin, 13005 Marseille, France; 76 Department of Pediatric Gastroenterology, Hepatology and Genetic Diseases, Children’s Hospital and University of Child Health Sciences, Lahore, Pakistan; 77 CENTOGENE GmbH, Am Strande 7, 18055 Rostock, Germany; 78 Department of Radiology, Great Ormond Street Hospital for Children, London, UK; 79 Institute of Medical Genetics, University Hospital of Wales, Cardiff CF14 4XW, UK; 80 Division of Cancer and Genetics, School of Medicine, Cardiff University, Cardiff CF14 4XW, UK; 81 Developmental Biology and Cancer Section, University College London Great Ormond Street Institute of Child Health, London, UK

**Keywords:** intellectual disability, neurodevelopmental disorder, zebrafish, disease model, tRNA modification, TRMT1

## Abstract

The post-transcriptional modification of tRNAs plays a crucial role in tRNA structure and function. Pathogenic variants in tRNA-modification enzymes have been implicated in a wide range of human neurodevelopmental and neurological disorders. However, the molecular basis for many of these disorders remains unknown. Here, we describe a comprehensive cohort of 43 individuals from 31 unrelated families with bi-allelic variants in tRNA methyltransferase 1 (*TRMT1*). These individuals present with a neurodevelopmental disorder universally characterized by developmental delay and intellectual disability, accompanied by variable behavioral abnormalities, epilepsy, and facial dysmorphism. The identified variants include ultra-rare *TRMT1* variants, comprising missense and predicted loss-of-function variants, which segregate with the observed clinical pathology. Our findings reveal that several variants lead to mis-splicing and a consequent loss of TRMT1 protein accumulation. Moreover, cells derived from individuals harboring *TRMT1* variants exhibit a deficiency in tRNA modifications catalyzed by TRMT1. Molecular analysis reveals distinct regions of TRMT1 required for tRNA-modification activity and binding. Notably, depletion of Trmt1 protein in zebrafish is sufficient to induce developmental and behavioral phenotypes along with gene-expression changes associated with disrupted cell cycle, immune response, and neurodegenerative disorders. Altogether, these findings demonstrate that loss of TRMT1-catalyzed tRNA modifications leads to intellectual disability and provides insight into the molecular underpinnings of tRNA-modification deficiency caused by pathogenic *TRMT1* variants.

## Teaser

We identify bi-allelic variants in TRMT1, encoding a tRNA-modification enzyme, that cause intellectual disability and developmental delay. Functional studies in human cells and zebrafish provide insight into the molecular and cellular processes impacted by tRNA-modification deficiency that could underlie the pathology of TRMT1-linked disorders.

## Introduction

Intellectual disability is a neurodevelopmental disorder characterized by significant limitations in intellectual ability and adaptive function, with a prevalence estimated between 2% and 3% in the general population.^1^ Genomic sequencing studies have identified an increasing number of causative monogenic variants for intellectual disability in genes encoding a diverse group of proteins. Notably, pathogenic variants in genes encoding RNA-modification enzymes have been identified as the cause of several cognitive disorders in the human population.^2^^,^^3^^,^^4^ These findings highlight the emerging role of tRNA modification in normal neurological development and function.

Human tRNA methyltransferase 1 (TRMT1) is a tRNA-modification enzyme that catalyzes the formation of *N*2,*N*2-dimethylguanosine (m2,2G) in cytosolic and mitochondrial tRNAs.^5^^,^^6^ TRMT1 generates nearly all m2,2G modifications in the tRNA of human cells.^6^^,^^7^^,^^8^ The m2,2G modification has been proposed to play a role in tRNA structure and function.^9^^,^^10^^,^^11^ Human cells deficient in TRMT1 exhibit decreased global protein synthesis and reduced cellular proliferation.^6^ TRMT1 has also been found to be a cleavage target of the SARS-CoV-2 main protease, suggesting that perturbation of tRNA-modification patterns contributes to the cellular pathology of SARS-CoV-2 infection.^12^^,^^13^ Intriguingly, neuronal activation induces a change in the subcellular distribution of TRMT1, suggesting a role for TRMT1-catalyzed tRNA modification in neuronal transmission and plasticity.^7^

Frameshift variants in *TRMT1* have been identified as the cause of autosomal-recessive intellectual disability through exome sequencing (MIM: #618302).^14^^,^^15^^,^^16^^,^^17^ This was followed by the identification of a single homozygous missense variant in *TRMT1* associated with developmental delay, intellectual disability, and epilepsy.^18^ These studies suggest that TRMT1 protein function plays a key role in normal neurodevelopment and cognitive function. However, the impact of *TRMT1* variants on protein accumulation and function remains unknown for most cases. Moreover, the sparse number of *TRMT1* variants that have been identified and characterized has limited our understanding of cognitive disorders associated with *TRMT1* and their physiological consequences.

In humans, *TRMT1* appears to be ubiquitously expressed across all tested tissues (https://www.proteinatlas.org/ENSG00000104907-TRMT1). Moreover, *TRMT1* is expressed to comparable levels in the human brain with slightly higher expression in the cerebellum and cortex (https://gtexportal.org/home/gene/TRMT1). TRMT1 protein also appears to accumulate ubiquitously in the brain according to a proteomic map of anatomically distinct regions of the human brain.^19^ *TRMT1* also exhibits similar levels of expression in different regions of mouse and pig brains.^20^ Transcriptomics analysis of early fetal to late childhood human individuals finds that *TRMT1* exhibits generally similar levels of expression in major brain regions, with slightly decreased expression in all brain regions from gestation to birth that remains steady thereafter.^21^^,^^22^^,^^23^ These studies suggest that the underlying causes for the neurodevelopmental phenotypes associated with pathogenic *TRMT1* variants are not simply correlated with the levels or timing of mRNA or protein expression in tissues.

Here, we describe 43 affected individuals from 31 unrelated families presenting with clinical features of intellectual disability in which exome or genome sequencing identified ultra-rare bi-allelic segregating *TRMT1* variants. To functionally characterize the bi-allelic *TRMT1* variants, we explored tRNA modifications in proband-derived fibroblasts or lymphoblasts and quantified m2,2G modifications in cellular tRNAs. Moreover, we investigated the effects of *TRMT1* variants on reconstitution of activity and interaction between TRMT1 protein and tRNAs. Finally, we generate and characterize an animal model of TRMT1 deficiency that provides insight into the cellular mechanisms and pathways linked to the neurodevelopmental phenotypes. These studies significantly expand the spectrum of disease-causing *TRMT1* variants and elucidate the molecular underpinnings of *TRMT1*-derived disorders.

## Subjects, material, and methods

### Identification and recruitment of affected individuals

The families with bi-allelic *TRMT1* variants were identified using the GeneMatcher platform^24^ and data sharing with collaborators. Informed consent for genetic analyses was obtained from all subjects. Clinical details of the cohort were obtained by the follow-up of affected individuals. Seizure description is reported in line with the most recent International League Against Epilepsy guidance.^25^ Parents and legal guardians of all affected individuals gave their consent for the publication of clinical and genetic information according to the Declaration of Helsinki, and the study was approved by the Research Ethics Committee, Institute of Neurology, University College London (IoN UCL) (07/Q0512/26) and the local Ethics Committees of each participating center. Consent has been obtained from a subset of families to publish medical photographs and video examinations. Brain magnetic resonance imaging (MRI) scans were obtained from 12 affected individuals and were reviewed by an experienced team of pediatric neuroradiologists. Cerebellar atrophy, callosal thinning, and calvarial deformities were defined using standardized criteria.^26^^,^^27^^,^^28^ Facial photographs and/or videos of 24 individuals from 14 families were reviewed. Their dysmorphic features were described using terminology recommended by Elements of Morphology. Where no term was available for a dysmorphic feature seen in an individual, Human Phenotype Ontology terminology was used instead. All details on individuals and variant data can be found in Tables S1, S2, S3, and S4.

### Identification and interpretation of variants from genomic sequencing data

Single-nucleotide variations were identified by whole-exome sequencing or whole-genome sequencing in all individuals. Exomes or genomes were captured and sequenced on Illumina sequencers as described elsewhere^29^ in Macrogen, Korea or at collaborating centers (see Table S1). The bioinformatics filtering strategy included screening for only exonic and donor/acceptor splicing variants. Rare variations present at a frequency above 1% in gnomAD v.3.1.2 (https://gnomad.broadinstitute.org/) or present from exomes or genomes within datasets from UK Biobank and UK 100,000 genome project, or from internal research databases (e.g., Queen Square Genomics and UCL SYNaPS Study Group), were excluded. Candidate variants were then inspected with the Integrative Genomics Viewer and confirmed by Sanger sequencing in all the families. Sequence variants in *TRMT1* were described according to the recommendations of the Human Genome Variation Society and are based on reference sequence GenBank: NM_001136035. Sequence candidate variants were interpreted according to American College of Medical Genetics and Genomics guidelines.^30^

### Cell culture of primary dermal fibroblasts

Primary dermal fibroblasts were obtained from a skin biopsy of subjects. Fibroblasts were cultured in Dulbecco’s modified Eagle’s medium (DMEM; Thermo Fisher Scientific, Waltham, MA) supplemented with 10% fetal bovine serum (FBS; GE Healthcare) and penicillin-streptomycin (100 U/mL and 100 mg/mL, respectively; Thermo Fisher Scientific). For all experiments, the same passage number of subject and control fibroblasts was used. Primary fibroblasts were regularly tested for mycoplasma contamination and confirmed to be mycoplasma free.

### Cell culture of primary lymphoblasts

Lymphoblastoid cell lines (LCLs) are generated by Epstein-Barr virus transformation of the B lymphocytes within the peripheral blood lymphocytes of individuals. LCLs were cultured in cells in RPMI 1640 medium (Thermo Fisher Scientific) supplemented with 10% fetal bovine serum (FBS; GE Healthcare) and penicillin-streptomycin (100 U/mL and 100 mg/mL, respectively; Thermo Fisher Scientific) in standing flasks at 37°C and 5% CO_2_ for several days until adhering to the flask and reaching a desired cell count.

### Minigene splicing assay

Computational assessment of splicing effects used SpliceSiteFinder-like, MaxEntScan, NNSplice, and GeneSplicer embedded in Alamut Visual Plus v.1.6.1 (Sophia Genetics, Bidart, France), as well as SpliceAI 10K and AbSplice as included in SpliceAI Visual.^31^ Results can be found in Table S5.

RNA studies of variants were conducted following established protocols with some modifications^32^^,^^33^ using three constructs with variants annotated to GenBank: NM_001136035.4. In brief, the first construct comprised a 1,002-bp region spanning introns 2–5, encompassing the c.255−1G>T, c.310+5G>C, c.311−1G>A, and c.454−1G>C variants. The second construct involved a 416-bp segment spanning introns 8–10 to assay splice effects of the c.1107−1G>A variant. Finally, the third construct covered a 446-bp region spanning intron 10 to exon 12, targeting the c.1194G>A variant. These regions were amplified from genomic DNA obtained from the probands and a healthy control using primers containing specific restriction sites (Table S6). The PCR fragments were ligated between exons A and B of the linearized pSPL3 vector following digestion with restriction enzymes. The recombinant vectors were transformed into DH5α competent cells (NEB 5-alpha, New England Biolabs, Frankfurt, Germany), plated, and incubated overnight. Following colony PCR with SD6 F (Table S6) and the target-specific reverse primer, the wild-type (WT) and mutant-containing vector sequences were confirmed by Sanger sequencing and transfected into HEK 293T cells (ATCC, Manassas, VA, USA). 2 μg of the respective pSPL3 vectors was transiently transfected using 6 μL of FuGENE 6 Transfection Reagent (Promega, Walldorf, Germany). An empty vector and transfection negative reactions were included as controls. The transfected cells were harvested 24 h after transfection. Total RNA was isolated using an miRNeasy Mini Kit (Qiagen, Hilden, Germany). cDNA was synthesized using the High-Capacity cDNA Reverse Transcription Kit (Applied Biosystems, Waltham, MA, USA) following the manufacturer’s protocols. cDNA was PCR amplified using vector-specific SD6 F and SA2 R primers (Table S6). The amplified fragments were visualized on a 1% agarose gel. cDNA amplicons were TA cloned following standard protocols with the pCR2.1 vector kit (ThermoFisher, Darmstadt, Germany) and Sanger sequenced. Fragment analysis was performed for construct 1 with FAM-labeled SD6 F and SA2 R primers using the 3500xL Genetic Analyzer (Thermo Fisher Scientific). Analysis was performed using GeneMapper Software 5 (Applied Biosystems). Analysis and cataloging of protein-coding versus non-protein coding transcripts and their expression was performed using Ensembl and GTEx Portal. Non-coding transcripts were excluded in calculations to determine the average peak area for construct 1. The percentage of each band was calculated using fragment analysis (Figure S2, Data S1, and Table S7).

### Immunoblotting

For protein immunoblotting, fibroblast or lymphoblast cells were resuspended in hypotonic lysis buffer for protein extraction as noted previously.^18^^,^^34^^,^^35^ Cell extracts were boiled at 95°C for 5 min followed by fractionation on NuPAGE Bis-Tris polyacrylamide gels (Thermo Fisher Scientific). Separated proteins were transferred to Immobilon FL polyvinylidene difluoride (PVDF) membrane (Millipore) for immunoblotting. Membrane was blocked by Odyssey blocking buffer for 1 h at room temperature followed by immunoblotting with the following antibodies: anti-TRMT1 (sc-373687, Santa Cruz Biotechnology), anti-FLAG epitope tag (L00018, Sigma), and anti-actin (L00003, EMD Millipore). Proteins were detected using a 1:10,000 dilution of fluorescent IRDye 800CW goat anti-mouse immunoglobulin G (IgG) (925-32210; ThermoFisher).

### RNA analysis

RNA was extracted using TRIzol LS reagent (Invitrogen). For primer extension analysis, 1.5 μg of total RNA was pre-annealed with 5′-^32^P-labeled oligonucleotide and 5× hybridization buffer (250 mM Tris [pH 8.5] and 300 mM NaCl) in a total volume of 7 μL. The mixture was heated at 95°C for 3 min followed by slow cooling to 42°C. An equal amount of extension mix consisting of avian myeloblastosis virus reverse transcriptase (RT; Promega), 5× AMV buffer, and 40 μM dNTPs was added. The mixture was then incubated at 42°C for 1 h and loaded on 18%–20% 7 M urea denaturing polyacrylamide gels. Gels were exposed on a phosphor screen and scanned on a Sapphire Biomolecular Imager (Azure Biosystems). Quantification was performed using NIH ImageJ software followed by statistical analysis using GraphPad Prism. Primer extension oligonucleotide sequences were previously described.^6^ The percent (%) m2,2G RT block was calculated as the m2,2G stop band divided by the sum of the m2,2G stop band and the subsequent RT block band multiplied by 100.

### Liquid chromatography-mass spectrometry

RNAs were digested and processed by liquid chromatography-mass spectrometry (LC-MS) as described previously by our lab.^12^^,^^18^ In brief, 1 μg of total RNA from human tissue culture cells or zebrafish tissue was digested for 3 h at 37°C in 250 mM Tris-HCl (pH 8.0), 5 mM MgCl_2_, 50 units of benzonase, 5 units of CIP, 0.5 units of phosphodiesterase I, and 100 μg/mL pentostatin in a total volume of 50 μL. Ribonucleosides were purified using an Amicon Ultra Centrifugal 10-kDa molecular-weight cutoff filter. Ribonucleosides were separated using a Hypersil GOLD C18 Selectivity Column (Thermo Scientific) followed by nucleoside analysis using a Q Exactive Plus Hybrid Quadrupole-Orbitrap. The modification ratio was calculated using the *m*/*z* intensity values of each modified nucleoside following normalization to the sum of intensity values for the canonical nucleosides A, U, G, and C as previously described.^36^

### Transient transfection of 293T cells

The 293T *TRMT1*-knockout (KO) cell line has been described previously.^6^ 293T cells were transfected via the calcium phosphate transfection method.^37^ In brief, 2.5 × 10^6^ cells were seeded on 100 × 20-mm tissue culture grade plates (Corning) followed by transfection with 10 μg of plasmid DNA. Cells were harvested 48 h later by trypsin and neutralization with medium, followed by centrifugation of the cells at 700 × *g* for 5 min, a subsequent PBS wash, and a second centrifugation step.

### Protein-RNA purifications

Protein was extracted using hypotonic lysis and high salt immediately after cells were harvested. Cell pellets were resuspended in 0.5 mL of hypotonic lysis buffer (20 mM HEPES [pH 7.9], 2 mM MgCl_2_, 0.2 mM EGTA, 10% glycerol, 0.1 mM PMSF, and 1 mM DTT) per 100 × 20-mm tissue culture plate. Cells were kept on ice for 5 min, then subjected to three freeze-thaw cycles in liquid nitrogen and a 37°C water bath. NaCl was then added to the extracts at a concentration of 0.4 M, incubated on ice for 5 min, and centrifuged at 14,000 × *g* for 15 min at 4°C. After centrifugation 500 μL of supernatant extract was removed, and 500 μL of hypotonic lysis buffer supplemented with 0.2% NP-40 was added to obtain 1,000 μL of extract.

FLAG-tagged proteins were purified by incubating whole-cell lysates from the transiently transfected cell lines with 50 μL of Anti-DYKDDDDK Magnetic Beads (Syd labs, PA004830) for 2 h at 4°C. Magnetic resin was washed three times in hypotonic wash buffer (20 mM HEPES [pH 7.9], 2 mM MgCl_2_, 0.2 mM EGTA, 10% glycerol, 0.1% NP-40, 0.2 M NaCl, 0.1 mM PMSF, and 1 mM DTT). SDS-PAGE sample buffer was added to one portion of resin, and purified proteins were fractionated on a NuPAGE Bis-Tris polyacrylamide gel (ThermoFisher). The gel was transferred to an Immobilon-FL Hydrophobic PVDF Transfer Membrane (Millipore Sigma) with subsequent immunoblotting against the FLAG tag or actin.

RNA from input and purified samples were extracted using RNA Clean & Concentrator-5 columns (Zymo Research, Irvine, CA, USA). RNA extraction followed the TRIzol LS RNA extraction protocol (Invitrogen). RNA was resuspended in 5 μL of RNase-free water and loaded onto a 10% polyacrylamide/7 M urea gel. The gel was then stained with SYBR Gold nucleic acid stain (Invitrogen) to visualize RNA. For northern blot analysis, RNA was transferred from gels onto an Amersham Hybond-XL membrane (GE Healthcare). The blot was probed with the following oligonucleotides: Ala-AGC-8-Tloop, 5′-GGAGGATGCGGGCATCGATC-3′ or Glu-TTC, 5′-TTCCCTGGCCGGGAATCG-3′. The oligos were radiolabeled using T4 polynucleotide kinase (NEB) with adenosine [γ-^32^P]triphosphate (6,000 Ci/mmol, Amersham Biosciences). Northern blots were visualized by Phosphor-Imager analysis and stripped via two incubations at 80°C for 30 min in a buffer containing 0.15 M NaCl, 0.015 M Na-citrate, and 0.1% SDS.

### Ethics statement and zebrafish husbandry

All experimental animal care was performed in accordance with institutional and NIH guidelines and regulations. Zebrafish (*Danio rerio*) were raised and maintained in an Association for Assessment and Accreditation of Laboratory Animal Care (AAALAC)-accredited facility at the Oklahoma Medical Research Foundation (OMRF) under standard conditions. All experiments were conducted as per protocol (22–76) approved by the Institutional Animal Care Committee (IACUC) of OMRF.

### Generation of *trmt1* knockout zebrafish

We used established methods to generate *trmt1* F0 knockouts in WT or *Tg*(*olig2*:*dsRed*);*nacre* embryos.^26^^,^^38^ In brief, three guide sequences were designed using the CRISPOR tool, and guide RNAs (gRNAs) were chemically synthesized by Synthego (Redwood City, CA, USA). A 6-μL mixture containing 1 μL of 40 μM Cas9-NLS protein (UC Berkeley QB3 Macrolab, Berkeley, CA, USA), 500 ng of each gRNA (in 3 μL), and 2 μL of 1 M potassium chloride was injected into one-cell-stage embryos. As a control, WT or transgenic embryos were injected with a mixture containing Cas9 protein but no single-guide RNA (sgRNA). F0 embryos were raised to adulthood and pairwise outcrossed with WT to obtain mosaic allele carriers, which were identified by genotyping their F1 progeny. Two carriers were subsequently inbred to generate *trans*-heterozygous embryos for functional analysis and RNA sequencing (RNA-seq). The gRNAs and primer sequences are listed in Table S8.

### RNA extraction, RT-qPCR, and RNA-seq

Total RNA was extracted from whole larvae or head-only samples using TRIzol reagent (Thermo Fisher Scientific) and purified with the RNA Clean and Concentrator-5 kit (Zymo Research), following the manufacturer’s instructions. At 4 days post fertilization (dpf), larvae were anesthetized in 168 mg/L tricaine methanesulfonate/MS-222 (Sigma-Aldrich, MO, USA) before head dissection. Each experimental group comprised three biological replicates, with six larvae randomly pooled per replicate. cDNA synthesis was performed using the iScript RT Supermix (Bio-Rad, Hercules, CA, USA) and subsequently used as a template for RT-qPCR with SYBR Green Supermix (Thermo Fisher Scientific) on the Light Cycler 96 System (Roche, Pleasanton, CA, USA). All RT-qPCR reactions were conducted in biological triplicates with technical triplicates, using *18S* as a reference. Primer sequences for RT-qPCR are listed in Table S8. Relative gene-expression levels were calculated using the 2^−ΔΔCt^ method, with cycle threshold (Ct) values analyzed in Microsoft Excel.

For RNA-seq analysis, total RNA was extracted from WT and *trans*-heterozygous larvae at 5 dpf using TRIzol reagent and purified with the miRNeasy Mini kit (Qiagen, Hilden, Germany) following the manufacturer’s protocols. Each experimental group consisted of four biological replicates, with six larvae pooled per replicate. Differentially expressed genes (DEGs), comparisons, and data visualization| were analyzed using the BxGenomics platform (BioInfoRx) and iDEP version 2.01 with DESeq2 package.^39^ Visualization included quality control and principal component analysis (PCA) plots, heatmaps, volcano plots, gene-expression plots, gene ontology (GO), and Kyoto Encyclopedia of Genes and Genomes (KEGG) pathway analyses. DEGs were identified based on a *p* value of <0.01 or false discovery rate (FDR) of <0.05 and a fold change >2.

The raw sequence reads of RNA0seq results in FASTQ format are available at https://www.ncbi.nlm.nih.gov/bioproject/PRJNA1198105.

### Morphological phenotyping

To evaluate morphological phenotypes, zebrafish larvae were randomly selected at 4 dpf for imaging. The larvae were manually positioned in 2% methylcellulose (Sigma) under a stereomicroscope for visualization and image capture. Morphological measurements, including head, eye, and body sizes, were obtained directly from scale-calibrated images using ImageJ software (NIH). Head size was defined as the distance from the tip of the snout to the end of the operculum (gill cover). Eye size was measured as the diameter of the eye, while body size was determined as the length from the tip of the snout to the end of the tail. Bright-field images were captured using a Nikon DS-Fi2 high-definition camera mounted on a Nikon SMZ18 stereomicroscope (Nikon, Japan) equipped with auto-*z*-stacking capability.

### Whole-mount immunohistochemistry

Whole-mount immunohistochemistry was performed to label brain anatomical structures following established protocols.^40^ To assess cell proliferation, an immunohistochemical protocol specific to dissected brain tissue was utilized.^41^ The antibodies used are rabbit anti-phospho-histone H3 antibody (1:500, Sigma-Aldrich 06-570), mouse anti-acetylated-tubulin antibody (1:500, Sigma-Aldrich T7451), rabbit anti-acetylated-tubulin antibody (1:250, Cell Signaling Technology #5335), and mouse anti-SV2A antibody (1:500, DSHB SV2). The secondary antibodies used are goat anti-mouse IgG Alexa Fluor 647 antibody, goat anti-rabbit IgG Alexa Fluor 488 antibody (1:500, Jackson ImmunoResearch Laboratories, West Grove, PA, USA). Samples were mounted in 1.2% agarose and imaged using a Zeiss LSM710 with EC Plan-Neofluar 10×/0.3 NA Ph1 and Plan-Apochromat 20×/0.8 NA objectives.

### Behavioral assay

All behavior tests were conducted at room temperature, as previously described.^42^ In brief, to perform the light/dark transition (LDT) test, larvae at 4 dpf were carefully transferred into individual wells of a 96-well plate, each containing 150 μL of embryo water. The next day, the plate was placed in a Noldus chamber, and locomotion activity was recorded using the DanioVision system equipped with EthoVision XT software (Noldus Information Technology, Leesburg, VA, USA). At 5 dpf, larvae were given a 30-min habituation period in light, followed by two cycles of alternating 30-min dark and light periods. Locomotor activity was measured as the distance traveled (in millimeters) per minute. The minute-by-minute data were plotted using GraphPad Prism (GraphPad Software, San Diego, CA, USA). At 6 dpf, larvae were tested for acoustic-evoked behavioral response (AEBR) using the Zebrabox system (ViewPoint Life Sciences, Montreal, Canada). AEBR was quantified as the percentage of responses to 12 acoustic stimuli per larva. Data were visualized using box-and-whisker plots generated in GraphPad Prism. Error bars represent the range from the minimum to the maximum values, with the median indicated by the line within the box.

### Statistics

The statistical analysis was conducted using GraphPad Prism. Data are presented as indicated in figure legends. For all analyses, the significance level was set at 0.05. Significance was determined using a two-tailed unpaired Student’s t test with Welch’s correction for two comparisons, as detailed in the figure legends. *p* values were represented as follows: not significant (ns), *p* ≥ 0.05; ^∗^*p* < 0.05; ^∗∗^*p* < 0.01; ^∗∗∗^*p* < 0.001; and ^∗∗∗∗^*p* < 0.0001.

## Results

### Identification of pathogenic variants in *TRMT1* linked to neurodevelopmental disorders

**Figure 1:**
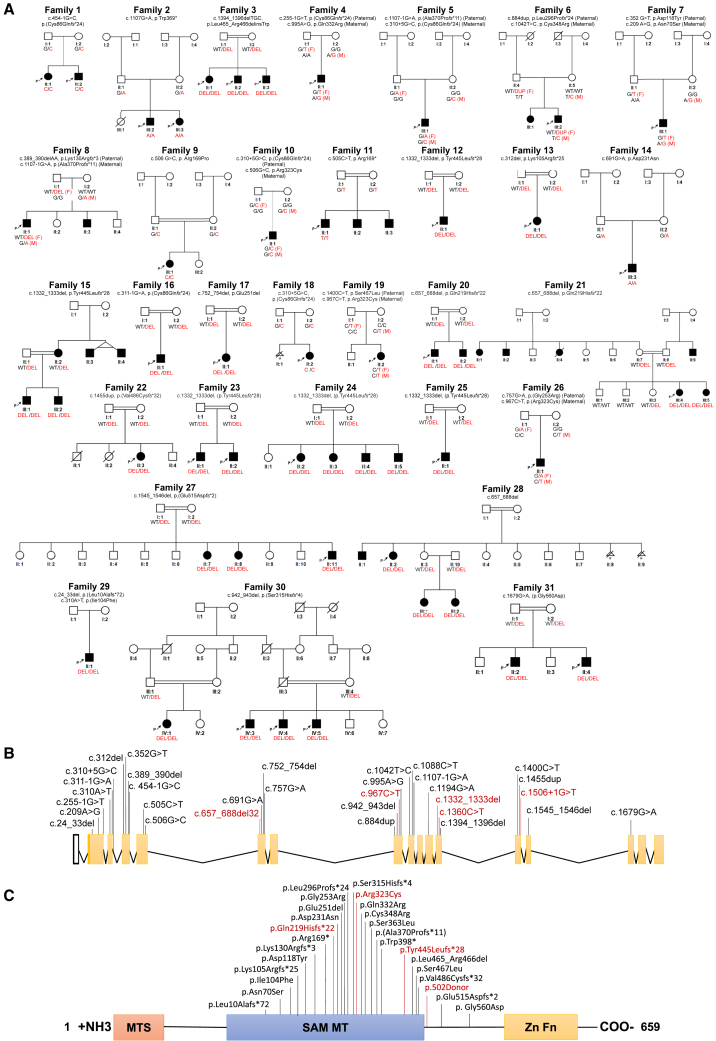
Genetic pedigrees of the reported individuals with homozygous *TRMT1* variants (A) Pedigrees of the families described. Squares, males; circles, females; black symbols, affected individuals; white symbols, unaffected individuals. Double lines indicate consanguinity. The text below each affected individual describes their alleles with variant alleles in red. (B) Coding exons of the *TRMT1* mRNA with variants noted. (C) Schematic indicating the domains of the TRMT1 protein. The red box represents the mitochondrial targeting signal (MTS), while the blue box indicates the class I *S*-adenosyl-methionine-dependent methyltransferase (SAM MT) domain. The yellow box indicates a C-terminal bipartite nuclear localization signal embedded within a C_3_H_1_-type zinc finger (Zn Fn) motif. Variants reported in this study are represented in black, while previously reported variants are in red.

Using the GeneMatcher platform and data sharing with collaborators, we identified 31 unique families containing 43 individuals affected with neurodevelopmental disorders secondary to bi-allelic variants in *TRMT1* (Figure 1A; Tables S1 and S2). We identified 11 missense, 2 nonsense, 13 splice site, and 8 frameshift variants. The missense variants were classified as damaging by SIFT, PolyPhen-2, REVEL, and Mutation Taster, with a mean CADD score of 27. None of the variants identified were present in the homozygous state in gnomAD v.3.1.2. Nine of the 24 identified *TRMT1* variants were absent across multiple genetic databases (∼1 million alleles), whereas the remaining variants appear to be ultra-rare (Table S1).

All detected variants were located within the conserved *S*-adenosylmethionine-dependent methyltransferase domain (Figures 1B and 1C; GenBank: NM_001136035.4 and NP_001129507). Of the missense variants, c.506G>C (GenBank: NM_001136035.4) (p.Arg169Pro), c.691G>A (p.Asp231Asn), and c.967C>T (p.Arg323Cys) are conserved from yeast to humans, while c.995A>G (p.Gln332Arg), c.1042T>C (p.Cys348Arg), and c.1400C>T (p.Ser467Leu) are semi-conserved (Figure S1). c.1332_1333del (p.Tyr445Leufs^∗^28) was found in two independent individuals (F12:S1 [II:1] and F15:S1 [III:1]) of Kurdish and Turkish origin, respectively. Similarly, c.657_688del (p.Gln219Hisfs*^∗^*22) was found in two independent families of Pakistani (F20) and Iranian (F21) origin. Overall, these recurring variants suggest a possible founder effect.

Among the 43 affected individuals, 14 individuals were identified with homozygous variants in *TRMT1*, and nine individuals contained compound heterozygous variants in *TRMT1*. Among the cohort, 27 individuals were male (63%) and 16 were female (37%). Consanguinity was reported in 18 families (58%), while two were likely consanguineous due to shared village origins of the grandparents, and the remaining 11 families were non-consanguineous. The median age at last follow-up was 11 years (interquartile range [IQR]: 14 years, ranging from 2 years 2 months to 47 years) (Table S2).

### Clinical features of individuals with bi-allelic *TRMT1* variants

**Figure 2:**
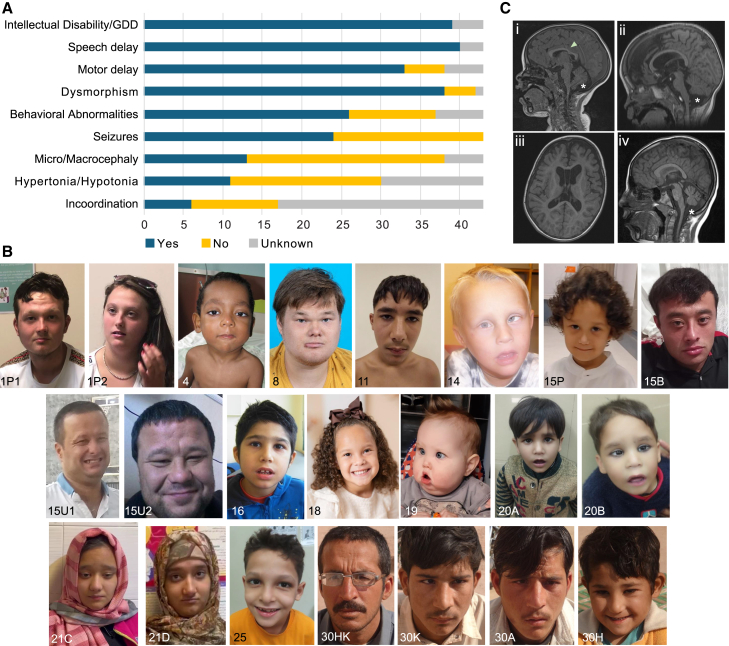
Genetic and phenotypic summary of the reported individuals with homozygous *TRMT1* variants (A) Clinical features of the affected individuals with bi-allelic *TRMT1* variants. GDD, global developmental delay. (B) Frontal facial photographs of *TRMT1* probands showing the most prominent and frequent dysmorphic features of *TRMT1*-related neurodevelopmental delay. (C) Representative neuroimaging features identified in individuals with intellectual disability. (i) Midsagittal T1-weighted MRI of the brain in a four-year-old boy (F-5) exhibits global (cerebral and cerebellar) atrophy, posterior thinning of the corpus callosum (arrow), and a mega cisterna magna (asterisk). (ii and iii) Midsagittal (ii) and axial (iii) T1-weighted MRI of the brain in a 4-year-old boy (F-8) shows further characteristic features of intellectual disability associated with *TRMT1*, namely, frontotemporal-predominant cerebral and midbrain atrophy with corresponding ventriculomegaly and uniform thinning of the corpus callosum (not all shown). Note is also made of the right posterior positional plagiocephaly. (iv) Midsagittal T1-weighted brain MRI of 7-year-old boy (F-23) exhibits cerebellar atrophy, a mega cisterna magna (asterisk), and downsloping of the corpus callosum.

The 43 affected individuals with bi-allelic *TRMT1* variants exhibited a core set of phenotypic features encompassing developmental delays, intellectual disability, and facial dysmorphism (Figures 2A and 2B). Case reports and detailed clinical history are provided in supplemental information and Table S2. Video recordings are available for affected individuals from family 1 (Videos S1 and S2).

Intellectual disability or global developmental delay (GDD) for individuals younger than 5 years of age was reported in all individuals who were tested (39/39). Intellectual disability/GDD varied in severity among individuals, and was assessed as mild/moderate in 79% (27/34) and severe/profound in 21% (7/34). Speech and language development was delayed among all participants tested (40/40), with the median age for first words spoken recorded at 24 months (range: 12 months to 8 years; IQR: 12). F21:S1 (III:4 in Figure 1) and F28:S2 (III:5) had absent speech at the ages of 19 and 30 years, respectively. A diverse range of behavioral issues were reported in 70% (*n* = 26/37) individuals, ranging from diagnosed autism spectrum disorder and attention-deficit/hyperactivity disorder to parent-reported concerns such as hyperactivity, aggression, anxious behavior, restlessness, poor autonomy, and irritability. Additionally, four individuals were reported to have motor/verbal tics (F1:S1 [II:1], F5:S1 [III:1], F15:S1 [III:1], and F23:S2 [II:2]). Feeding difficulties were reported in 40% of individuals (*n* = 16/40), encompassing issues such as chewing difficulties, choking, restrictive food choices, and the need for PEG feeding in F19:S1 (II:2). Additionally, 15% (*n* = 6/40) had poor weight gain, while three individuals (F7:S1 [II:1], F8:S1 [II:1], and F11:S1 [II:1]) exhibited obesity/overeating.

Motor milestones were delayed in the majority of tested individuals (87%, 33/38), with median ages of 10 months for unsupported sitting and 23.5 months for independent walking. F4:S1 (II:1) and F19:S1 (II:2) did not achieve independent ambulation at their last follow-up at 12 years and 2 years 8 months. In contrast, F8:S1 (II:1) began walking at 15 months but experienced regression following a status epilepticus episode at that age and did not achieve independent walking by 19 years. Other motor manifestations included an unsteady/broad-based gait (*n* = 2), clumsiness (*n* = 7), poor coordination, ataxia (*n* = 8) and tremors (*n* = 4). Seizures occurred in 56% of individuals (24/43), with 81% of individuals (13/16) having onset within the first two years of life. Seizure semiology varied and included febrile (*n* = 8), focal (*n* = 3), and generalized seizures (*n* = 8). 46% (*n* = 12/26) of the conducted EEGs were reported as abnormal for these individuals. Microcephaly was present in 29% (*n* = 11/38) of individuals. When data on follow-up orofacial cleft data were available, 67% (*n* = 4/6; F12:S1 [II:1], F19:S1 [II:2], F22:S1 [II:3], and F25:S1 [II:1]) developed secondary microcephaly, while 33% (*n* = 2/6; F4:S1 [II:1] and F16:S1 [II:1]) exhibited congenital microcephaly. Additionally, two affected individuals had macrocephaly (F3:S3 [II:3] and F7:S1 [III:1]). Moreover, short stature was observed in 18% (*n* = 7/39) individuals, while failure to thrive was noted in 15% (*n* = 6/40). Of these, four individuals exhibited both low height and weight (F4:S1 [II:1], F22:S1 [II:3], F24:S1 [II:2]. and F25:S1 [II:1]).

Facial photographs and/or videos were reviewed for 13 individuals from ten families (Figure 2B and Table S3, feature frequencies tabulated in Table S4). Based on this assessment, the most frequently seen facial dysmorphic features of *TRMT1*-related neurodevelopmental delay include high anterior hairline (54.2%), narrow forehead/bifrontal/bitemporal narrowing (54.2%), full or broad nasal tip (70.8%), and thin upper lip (45.8%). The facial features found are relatively non-specific, and recognizable facial gestalt for this disorder was not appreciated.

Neurological assessment revealed hypotonia in 23% (*n* = 7/30). F5:S1 (III:1) and F22:S1 (II:3) presented with hypotonia with normal deep tendon reflexes, while F25:S1 (II:1) and F26:S1 (II:1) exhibited early-onset hypotonia that later resolved. Hypertonia was observed in 10% (*n* = 3/30), with F8:S1 (II:3) and F16:S1 (II:1) displaying hypertonia of only the lower limbs, and F29:S1 (II:1) exhibiting axial hypotonia combined with hypertonia of all four limbs and admixed rigidity. Notably, F16:S1 (II:1) exhibited progressive spastic diplegia secondary to hemiconvulsion-hemiplegia syndrome. Four individuals (F11:S1 [II:1], F13:S1 [II:1], F18:S1 [II:2], and F19:S1 [II:2]) had impaired hearing.

Brain MRI was available for 12 individuals, performed between 4 months and 17 years of age (Figure 2C, summarized in Figure S2). The most prevalent neuroimaging findings in our cohort were cerebral atrophy (7/12; 58%); cerebellar atrophy (6/12; 50%), which was either global (*n* = 2), limited to the vermis (*n* = 2), or limited to the cerebellar hemispheres (*n* = 2); and posterior thinning of the corpus callosum (5/12; 42%). Two individuals (family 5 and family 20 proband 1) exhibited global brain atrophy. Cerebral atrophy was typically frontotemporal predominant and resulted in corresponding ventriculomegaly in three families (families 8, 11, and 15). Uniform thinning of the corpus callosum was present in one individual (family 8). Mega cisterna magna was identified in two probands (families 5 and 16). One individual (family 16) had an incidental middle cranial fossa arachnoid cyst, while one individual was noted to have right posterior positional plagiocephaly (family 9). Altogether, these findings identify a core pattern associated with bi-allelic *TRMT1* variants that can co-occur with a diversity of dysmorphic, neurological, and behavioral phenotypes.

### *TRMT1* splice site variants lead to aberrant splicing

A subset of *TRMT1* variants is predicted to alter mRNA splicing patterns based upon *in silico* splice site prediction algorithms (Table S5). To test the effects of the *TRMT1* variants on splicing, we generated minigene splicing reporter plasmids cloned from the genomic DNA of a healthy WT donor or affected individuals. The splicing reporters were transfected into 293T human embryonic cells, and splicing was analyzed by RT-PCR, sequencing, and fragment analysis (Figure S3; Tables S6 and S7; Data S1).

**Figure 3:**
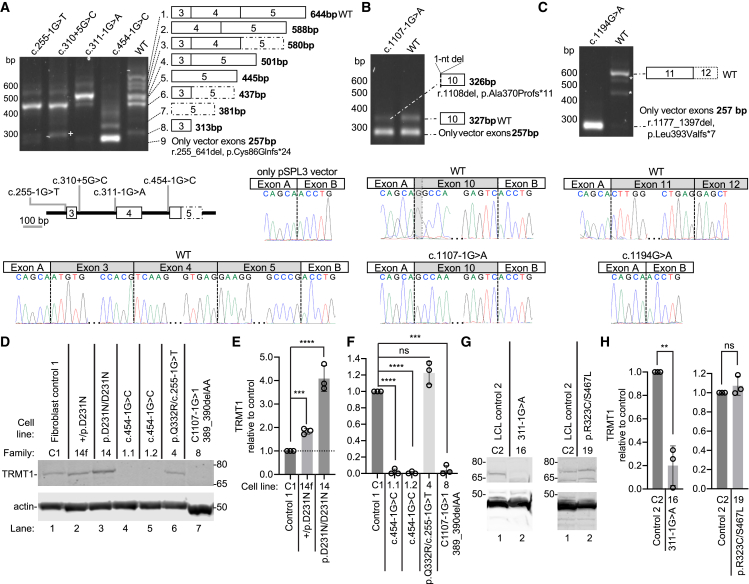
*TRMT1* variants induce splicing defects and changes in TRMT1 protein levels (A) RT-PCR analysis of RNA from HEK293T cells transfected with *TRMT1* minigenes. The presence of additional bands in the WT is attributed to alternative splicing and quantified in Table S7. The splicing schematic is shown for each band to the right. The variant schematic is shown below. Sanger sequencing results showing the correctly spliced WT product with the deleterious variant effect, exon skipping, shown by the presence of only pSPL3 vector. The dotted box represents the short version of exon 5. (B) RT-PCR analysis of RNA from cells transfected with the c.1107−1G>A minigene. The splicing schematic is shown for each band to the right. Exon 10 with 1-bp deletion is represented with a dotted line corresponding to the single-bp deletion. (C) RT-PCR of the c.1194G>A variant. Asterisks represent assay artifacts. The splicing schematic is to the right. Assay design captured part of exon 12 that was correctly spliced in the WT control. (D) Immunoblot of lysates from fibroblast cell lines derived from control (C1) or affected individuals. (E and F) Quantification of TRMT1 levels relative to the control fibroblast cell line after normalization to actin. (G) Immunoblot of lysates from lymphoblast cell lines (LCLs) derived from control WT (C2) or affected individuals. (H) Quantification of TRMT1 levels in LCLs after normalization to actin. *n* = 3. Error bars represent standard deviation from the mean. Statistical analysis was performed using one-way ANOVA. ^∗^*p* ≤ 0.05, ^∗∗^*p* ≤ 0.01, ^∗∗∗^*p* ≤ 0.001, ^∗∗∗∗^*p* < 0.0001; ns, non-significant (*p* > 0.05).

The c.255−1G>T and c.310+5G>C (GenBank: NM_001136035.4) variants are predicted to abolish the splice acceptor and donor sites of exon 3, respectively, while the c.311−1G>A variant is predicted to abolish the splice acceptor site of exon 4. The c.454−1G>C (GenBank: NM_001136035.4) variant is predicted to eliminate the splice acceptor site of exon 5. The c.255−1G>T, c.310+5G>C, c.311−1G>A, and c.454−1G>C variants were tested using a construct containing introns 2–5. RT-PCR spanning exons 3 through 5 from cells transfected with the WT construct showed a complex splicing pattern due to alternative splicing that was analyzed through Sanger sequencing and fragment analysis (Figure 3A and Data S1). From fragment analysis, a total of 53% of protein coding transcripts in WT include exons 3–5 that were completely eliminated in assays for the c.255−1G>T, c.310+5G>C, and c.454−1G>C variants with an abundance of only 10.6% in sample c.311−1G>A (r.255_641del [p.Cys86_Arg214del]) (Tables S5, S6, and S7).

The c.1107−1G>A variant was predicted to abolish the splice acceptor site of exon 10 and create a cryptic splice site 1 nt downstream of the native canonical splice site that likely causes a deletion of 1 bp and a frameshift r.1108del (p.Ala370Profs^∗^11). The c.1107−1G>A variant was evaluated using a construct spanning introns 8–10. RT-PCR of both WT and the c.1107−1G>A variant showed two bands, which we attribute to the pSPL3 vector (Figure 3B, gel). The frameshift was validated by Sanger sequencing (Figure 3B, chromatograms).

The splice prediction scores for the c.1194G>A variant suggested that either a cryptic donor gains 14 bp from the native splice acceptor site or no splice effect. The c.1194G>A variant was assayed using a construct spanning intron 10 to exon 12. The RT-PCR for the c.1194G>A variant showed skipping of exons 11 and 12 leading to a frameshift (r.1177_1397del [p.Leu393Valfs^∗^7]) that was validated with Sanger sequencing (Figure 3C). Altogether, our splicing analyses reveal that a subset of *TRMT1* variants can induce aberrant splicing that is expected to reduce mRNA abundance and/or produce altered protein products.

### *TRMT1* variants differentially impact TRMT1 protein levels

To examine the impact of intellectual disability-associated *TRMT1* variants, we next investigated TRMT1 protein accumulation in available cell lines using immunoblotting. For the p.Asp231Asn missense variant in family 14, we obtained fibroblast cells from the heterozygous father (individual 14f) and the homozygous offspring that were compared to a control fibroblast cell line (control 1, WT fibroblast). The heterozygous p.Asp231Asn fibroblasts exhibited a ∼2-fold increase in TRMT1 compared to WT fibroblast cells (Figure 3D, compare lanes 1 and 2; quantified in Figure 3E). The homozygous p.Asp231Asn fibroblast cell line exhibited an even greater ∼4-fold increase in TRMT1 levels compared to WT fibroblast cells (Figure 3D, compare lanes 1 and 3; quantified in Figure 3E). These results suggest that the p.Asp231Asn variant affects the folding of TRMT1 leading to increased stability against degradation and/or turnover.

We also generated fibroblast cell lines from individuals in families 1, 4, and 8 that harbor *TRMT1* splicing variants (Table S1). We detected nearly complete loss of TRMT1 accumulation in cell lines derived from two different members of family 1 who have a homozygous splicing variant that eliminates the splice acceptor site of exon 5 (Figure 3D, individuals 1.1 and 1.2, lanes 4 and 5; quantified in Figure 3F). Family 8 is compound heterozygous for a splice site and a frameshift variant in *trans*. TRMT1 accumulation is also reduced to nearly undetectable levels in fibroblasts from family 8 (Figure 3D, individual 8, lane 7; quantified in Figure 3F). The fibroblasts from individual 4 are compound heterozygous for the p.Gln332Arg missense variant and a splice variant that is predicted to abolish the splice acceptor of exon 3 (c.255−1G>T). We did not detect a significant change in TRMT1 levels in the fibroblasts from individual 4 compared to controls (Figure 3D, individual 4, lane 6; quantified in Figure 3F). No additional bands indicative of alternatively spliced or truncated proteins were detected in cell lines from family 1, 4, or 8 (full blots in Figure S4).

For individuals from families 16 and 19, we derived lymphoblastoid cell lines (LCLs) that were compared to control LCLs obtained from a healthy donor (control 2, WT LCL). We detected a substantial reduction in TRMT1 in the cell line from family 16 containing a homozygous splicing variant predicted to abolish the splice acceptor site of exon 4 (Figure 3G, quantified in Figure 3H; see Figure S4 for full blot). The individual from family 19 harbors compound heterozygous missense variants in *trans* in *TRMT1*. We detected no substantial change in TRMT1 levels in the individual 19 LCL compared to control LCLs (Figure 3G, quantified in Figure 3H). These results suggest that the p.Arg323Cys and p.Ser467Leu missense variants do not significantly affect TRMT1 levels. Altogether, these results demonstrate that *TRMT1* splice variants as well as certain missense variants can impact TRMT1 protein accumulation.

### Human cells with bi-allelic *TRMT1* variants exhibit a reduction in m2,2G modification in tRNAs

We next tested the functional impact of *TRMT1* variants on tRNA modification in the cell lines derived from individuals. TRMT1 has been shown to generate the m2,2G modification at position 26 in human tRNAs.^6^^,^^7^ To monitor the m2,2G modification, we used a primer extension assay in which the presence of m2,2G leads to a block of RT. A decrease in m2,2G modification allows for read-through and extension up to a subsequent RT-blocking modification. We performed the primer extension assay on tRNA-Met-CAU and mitochondria (mt)-tRNA-Ile-GAU, both of which contain m2,2G at position 26.^6^^,^^18^

**Figure 4:**
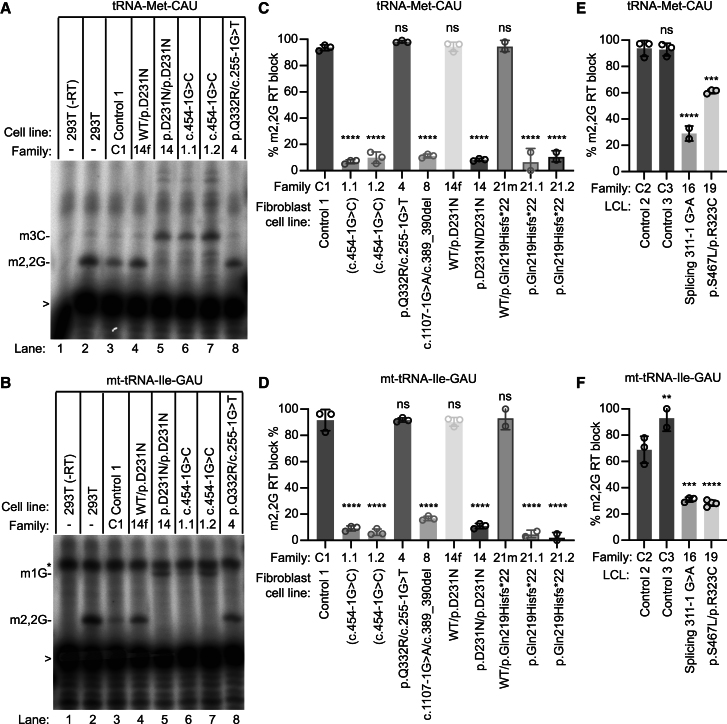
Human cell lines with bi-allelic *TRMT1* variants exhibit a reduction in m2,2G modifications in tRNAs (A and B) Representative gels of primer extension assays to monitor the presence of m2,2G in tRNA-Met-CAU and mt-tRNA-Ile-GAU from the indicated cell lines. m3C_20_, 3-methylcytosine; m2,2G_26_, dimethylguanosine; m1G_9_, 1-methylguanosine. “>” points to labeled oligonucleotide used for primer extension; asterisk denotes background signal. (C–F) Quantification of m2,2G formation by primer extension for the indicated tRNAs. % m2,2G RT block represents the m2,2G stop signal divided by the sum of the m2,2G and read-through m3C or m1G stop signal. The number of replicates is shown in each bar graph with a minimum of two replicates per cell line. Error bars represent standard deviation from the mean. Statistical analysis was performed using one-way ANOVA. For (C) and (D), the mean of each column was compared to the control 1 cell line. For (E) and (F), the mean of each column was compared to the control 2 cell line. ^∗^*p* ≤ 0.05, ^∗∗^*p* ≤ 0.01, ^∗∗∗^*p* ≤ 0.001, ^∗∗∗∗^*p* < 0.0001; ns, non-significant (*p* > 0.05).

As reference, we performed the primer extension assay with RNA extracted from 293T human embryonic cells. In the absence of RT, only background bands were detected in reactions containing the radiolabeled probe and RNA from 293T human cells (representative gels shown in Figures 4A and 4B, lane 1). Addition of RT led to the appearance of an extension product up to the m2,2G modification at the expected position in both tRNA-Met-CAU and mt-tRNA-Ile-GAU in 293T human embryonic cells and a control fibroblast cell line from a healthy control with WT *TRMT1* alleles (Figures 4A and 4B, lanes 2 and 3, 293T and WT control). Fibroblast cells from family 14 that are heterozygous for the p.Asp231Asn variant exhibit similar levels of m2,2G modification in tRNA-Met-CAU and mt-tRNA-Ile-GAU compared to the control fibroblast cell line (Figures 4A and 4B, compare lanes 3 and 4; quantified in Figures 4C and 4D). In contrast, fibroblast cells from family 14 that are homozygous for the p.Asp231Asn variant exhibited nearly complete loss of the m2,2G modification block in tRNA-Met-CAU and mt-tRNA-Ile-GAU (Figures 4A and 4B, compare lane 5 to lanes 3 and 4; quantified in Figures 4C and 4D). These results indicate that the p.Asp231Asn variant impairs the methyltransferase activity of TRMT1 to form m2,2G.

The m2,2G modification in tRNA-Met-CAU and mt-tRNA-Ile-GAU was also reduced in cell lines from families 1, 8, 16, 19, and 21 (Figures 4A, 4B, S5A, and S5B; quantified in Figures 4C–4F). The cell lines from families 1 and 16 are homozygous for *TRMT1* splicing variants, while the cell line from family 8 is compound heterozygous for a splice site and frameshift variant. The cell lines from family 21 are derived from the mother (21m) and the mother’s children (21.1 and 21.2) who are heterozygous or homozygous for a *TRMT1* frameshift variant, respectively. The reduction in m2,2G modification in cell lines with homozygous splice site and/or frameshift variants is consistent with the loss of full-length TRMT1 accumulation. The cell line from family 19 is compound heterozygous for the p.Ser467Leu/p.Arg323Cys missense variants. The reduction in m2,2G modification in this cell line indicates that the p.Ser467Leu and p.Arg323Cys variants reduce the activity of TRMT1.

No significant change in m2,2G modification was detected in the individual 4 cell line, which is compound heterozygous for the p.Gln332Arg missense and a splice site variant (Figures 4A and 4B, lane 8; quantified in Figures 4C and 4D). These results suggest that the combination of these two *TRMT1* alleles produces enough active protein to maintain m2,2G modification in tRNA-Met-CAU and mt-tRNA-Ile-GAU. This finding is consistent with our observation that the individual 4 cell line exhibits levels of TRMT1 comparable to those of WT human cells (Figure 4D).

To determine the effects of *TRMT1* variant alleles on total m2,2G levels, we used LC-MS to quantify m2,2G levels in RNA of individual cell lines. The m2,2G levels were reduced to near-background levels in cell lines 1.1, 1.2, 8, 14, 16, 19, 21.1, and 21.2 compared to control cell lines (Figures S6A and S6B). In contrast, fibroblast cells from the heterozygous father of individual 14 (14f) or the heterozygous mother of individuals 21.1 and 21.2 exhibited no significant change in m2,2G levels compared to the cell line derived from a healthy control donor (Figure S6A). Moreover, cell line 4 exhibited levels of m2,2G similar to those of control cells (Figure S6A). These results are consistent with the primer extension shown above and provide evidence that global m2,2G levels are perturbed in nearly all the individuals with bi-allelic *TRMT1* variants described here.

### TRMT1 protein variants exhibit defects in reconstituting m2,2G modification in cells

We next used a *TRMT1*-KO cell line derived from 293T human embryonic kidney cells to test *TRMT1* variants for their ability to rescue m2,2G formation *in vivo*. The *TRMT1*-KO cell line exhibits the absence of m2,2G modifications in all tested tRNAs.^6^ The TRMT1-deficient 293T cell line allowed us to further characterize the functionality of *TRMT1* variants, including variants for which cell lines were not available from families. As a comparison, we also tested a *TRMT1* c.1088C>T (GenBank: NM_001136035.4) (p.Ser363Leu) missense variant that was present as a minor allele in certain populations and is predicted to be non-pathogenic based upon mutation screenings.^43^

**Figure 5:**
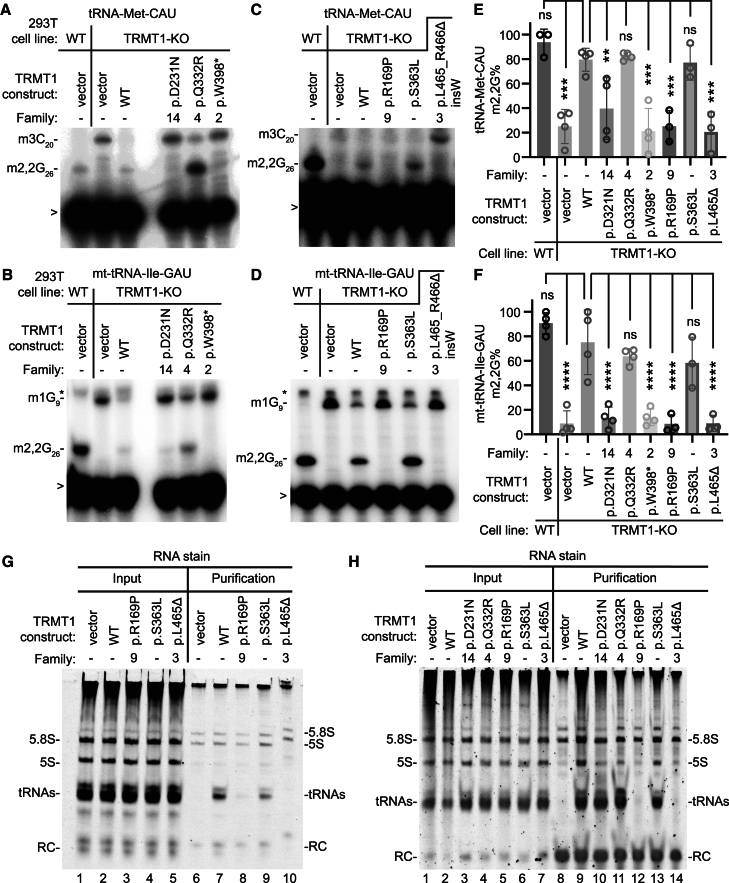
TRMT1 protein variants exhibit defects in reconstitution of tRNA-modification activity and interaction with tRNAs (A–D) Representative primer extension gels to monitor the presence of m2,2G in tRNA-Met-CAU and mt-tRNA-Ile-GAU from 293T cell lines transfected with the indicated constructs. m3C_20_, 3-methylcytosine; m2,2G_26_, dimethylguanosine; m1G_9_, 1-methylguanosine. “>” points to oligonucleotide used for primer extension; asterisk denotes background signal. (E and F) Quantification of m2,2G formation by primer extension for the indicated tRNAs. Primer extensions were performed at least three times per variant, and error bars represent the standard error of the mean. Statistical analysis was performed using one-way ANOVA. ^∗^*p* ≤ 0.05, ^∗∗^*p* ≤ 0.01, ^∗∗∗^*p* ≤ 0.001, ^∗∗∗∗^*p* < 0.0001; ns, non-significant (*p* > 0.05). (G and H) Nucleic acid stain of RNAs extracted from the indicated input or purified TRMT1-FLAG samples after denaturing PAGE. The migration pattern of tRNAs, 5.8S, and 5S ribosomal RNA is denoted. The p.L465_R466Δ insW variant is denoted as L465Δ in (E)–(H).

Using transient transfection of plasmid constructs, we expressed mRNAs encoding WT-TRMT1 or TRMT1 variants in the *TRMT1*-KO cell line. We then assessed for rescue of m2,2G formation in tRNA-Met-CAU or mt-tRNA-Ile-GAU using the primer extension assay described above. As expected, WT 293T cells transfected with vector alone exhibited an RT block at position 26 of tRNA-Met-CAU and mt-tRNA-Ile-GAU, indicative of the m2,2G modification (Figures 5A–5D, lane 1). The m2,2G modification was absent in tRNA-Met-CAU and mt-tRNA-Ile-GAU from the vector-transfected *TRMT1*-KO cell line leading to read-through to the next RT block (Figures 5A–5D, lane 2). Transfection of a plasmid encoding WT TRMT1 into the *TRMT1*-KO cell line was able to restore m2,2G formation (Figures 5A–5D, lane 3). Due to incomplete transfection efficiency that caused variable TRMT1 accumulation, the level of m2,2G modification was increased in the *TRMT1*-KO cell line but not completely rescued to the level of the original WT cell line.

Using this assay, we found that the TRMT1 p.Gln332Arg protein variant from family 4 exhibited similar reconstitution of m2,2G formation as WT TRMT1 (Figures 5A and 5B, lane 5, quantified in Figures 5E and 5F). The WT activity of the p.Gln332Arg variant is consistent with the WT levels of m2,2G modification detected in the tRNAs of the cell line derived from family 4 (Figures 5A–5D). The TRMT1 p.Ser363Leu minor variant also retained the ability to reconstitute m2,2G formation similar to that observed in WT TRMT1 (Figures 5C and 5D, lane 5; quantified in Figures 5E and 5F).

Notably, we found that the TRMT1 p.Asp231Asn variant from family 14 and TRMT1 truncation variant (1–398) from family 2 were greatly reduced in their ability to reconstitute m2,2G formation in the *TRMT1*-KO cell line (Figures 5A and 5B, lanes 4 and 6; quantified in Figures 5E and 5F). The reduced activity of the TRMT1 p.Asp231Asn variant from family 14 is consistent with the drastically reduced m2,2G levels in cells homozygous for the p.Asp231Asn variant (Figure 4). We also found that the p.Arg169Pro missense variant from family 9 and *TRMT1* c.1394_1396del (GenBank: NM_001136035.4) (p.Leu465_Arg466delinsTrp) variant from family 3 exhibited defects in reconstituting m2,2G formation in *TRMT1*-KO cell lines (Figures 5C and 5D, lanes 4 and 6; quantified in Figures 5E and 5F). These results suggest that individuals homozygous for these variants are likely to be deficient in m2,2G modifications.

### TRMT1 variants exhibit defects in tRNA binding

**Figure 6:**
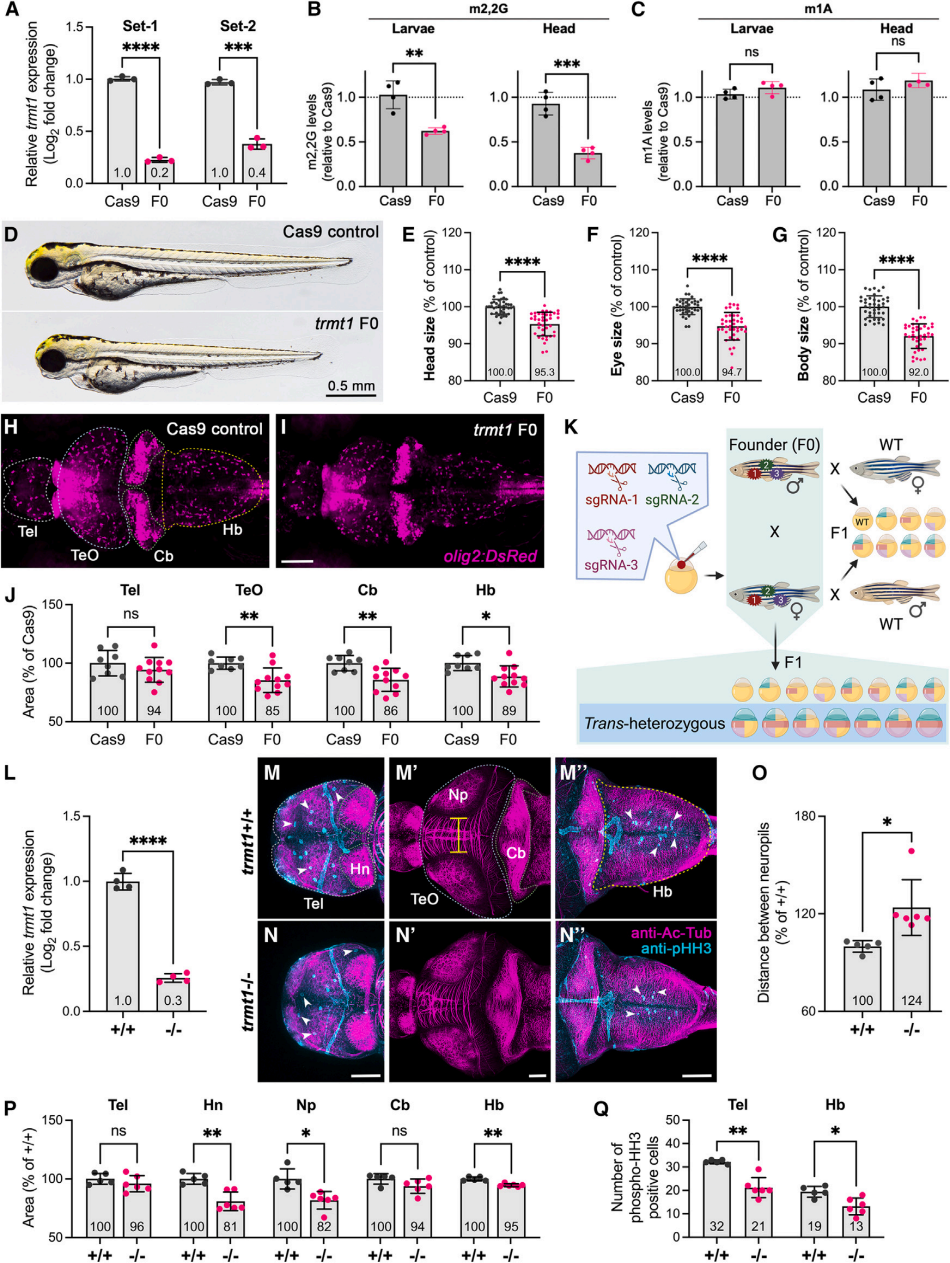
Depletion of Trmt1 in zebrafish causes developmental delay and reduced brain size due to decreased cell proliferation (A) RT-qPCR analysis of *trmt1* expression in Cas9-injected control and *trmt1* F0 knockout animals at 4 dpf. Expression levels were normalized to *18S* and compared to the Cas9 controls. (B and C) LC-MS analysis of m2,2G or m1A in whole larvae or head-only samples. (D) Representative image for Cas9-injected control (Cas9) and *trmt1* F0 knockout (F0) larvae at 3 dpf. (E–G) Quantifications of head, eye, and body sizes for Cas9 and *trmt1* F0 animals (*n* = 40 embryos per group). Values are presented as a percentage of the mean value of Cas9 controls. Each dot represents one larva. (H and I) Representative live confocal images of Cas9 and *trmt1* F0 larvae in *Tg*(*olig2*:*dsRed*);*nacre* reporter line at 5 dpf. Images are dorsal view with anterior to the left. dsRed is shown in magenta. Scale bar, 0.1 mm. (J) Quantification of brain regions as depicted in (H) for Cas9 (*n* = 8 larvae) and F0 (*n* = 11 larvae) larvae. (K) Schematic illustrating the experimental design: three sgRNAs targeting *trmt1* exons were injected into one-cell-stage embryos to generate F0 knockouts. Sexually mature F0 knockouts were bred with WT fish, and the resulting F1 progeny were genotyped to identify inheritable mutant allele carriers. Positive F0 founder carriers were inbred to obtain *trans*-heterozygous (−/−) F1 knockout progeny. (L) RT-qPCR analysis of *trmt1* expression in WT (+/+) control and *trmt1* F1 knockout (−/−) larvae at 5 dpf. Experiments were performed with four biological replicates in technical triplicates. Expression levels were normalized to *18S* and compared to the WT controls. (M and N) Confocal images of dissected *trmt1*^+/+^ (M to M″, *n* = 5 brains) and *trmt1*^−/−^ (N to N″, *n* = 6 brains) larval brain at 5 dpf, stained with anti-acetylated tubulin (Ac-Tub, magenta) and anti-phospho-histone H3 (pHH3, cyan). (M) and (N) show the telencephalon, (M′) and (N′) show the optic tectum and cerebellum, and (M″) and (N″) show the hindbrain. Images are dorsal view with anterior to the left. Brain regions are outlined with a dotted line, and pHH3-positive cells are indicated by white arrowheads. Scale bars, 50 mm. (O) Quantification of the distance between two neuropils as indicated in (M′). (P) Quantifications of areas defined by dotted lines in (M), (M′), and (M″). (Q) Quantification of the number of phospho-histone H3-positive cells in telencephalon and hindbrain. Error bars indicate mean ± SD. For (L), (O), and (P), values are presented as a percentage of the mean value of *trmt1*^+/+^ controls. Mean values are displayed at the bottom of each bar. Statistical significance was calculated by unpaired t test with Welch’s correction: ns, non-significant (*p* > 0.05); ^∗^*p* < 0.05, ^∗∗^*p* < 0.01, ^∗∗∗^*p* < 0.001, ^∗∗∗∗^*p* < 0.0001. Tel, telencephalon; Hb, habenula; TeO, optic tectum; Np, tectal neuropil; Cb, cerebellum; Hb, hindbrain.

We next investigated the interaction between TRMT1 variants and tRNAs to dissect the molecular defects associated with individual TRMT1 variants. We have previously shown that human TRMT1 displays a stable interaction with substrate tRNAs that are targets for m2,2G modification.^6^^,^^18^ Using this system, we expressed an FLAG-tagged version of the TRMT1 variants in 293T human embryonic kidney cells followed by affinity purification and analysis of copurifying RNAs. Sample recovery of copurifying RNAs was confirmed through the spike-in addition of a synthetic RNA that served as a recovery control. We analyzed the same set of TRMT1 variants as in Figure 6. Immunoblotting confirmed the accumulation and purification of each TRMT1 variant on anti-FLAG resin (Figures S7A and S7B).

In the control purification from vector-transfected cells, we detected only background contaminating 5.8S and 5S ribosomal RNAs without tRNAs (Figure 5G, lane 6; Figure 5H, lane 8). In contrast, the purification of WT TRMT1 resulted in the enrichment of tRNAs along with rRNAs as we have previously shown (Figure 5G, lane 7; Figure 5H, lane 9). Using northern blotting to test specificity, we detected enrichment of m2,2G-containing tRNA-Ala isoacceptors with WT TRMT1, while tRNA-Glu-UUC lacking m2,2G exhibited only background binding to TRMT1 (Figure S7C).

In contrast to WT TRMT1, we found that the TRMT1 p.Arg169Pro variant from family 9 and p.Leu465_Arg466delinsTrp variant in family 3 exhibited defects in binding to tRNAs compared to WT TRMT1 (Figure 5G, lanes 8 and 10; repeated in Figure 5H, lanes 12 and 14). Further confirming the binding defect, the enrichment of tRNA-Ala isoacceptors was abolished with the TRMT1 p.Arg169Pro and p.Leu465_Arg466delinsTrp deletion variants compared to WT TRMT1 (Figure S7C). The reduced tRNA binding by the TRMT1 p.Arg169Pro and p.Leu465_Arg466delinsTrp deletion variants could explain their diminished ability to reconstitute m2,2G formation in cells (Figures 5C and 5D). The TRMT1 p.Ser363Leu minor variant exhibited similar binding to tRNAs compared to WT TRMT1. The WT tRNA binding of the TRMT1 p.Ser363Leu variant is consistent with the WT activity of this variant in reconstitution assays observed above.

The TRMT1 p.Asp231Asn and p.Gln332Arg variants retained interaction with tRNAs similar to WT TRMT1 (Figure 5H, compare lane 9 to lanes 10 and 11). This result indicates that the p.Asp231Asn substitution perturbs an enzymatic step separable from substrate tRNA binding such as SAM binding, methyl transfer, or catalysis, since the TRMT1 p.Asp231Asn variant causes loss of m2,2G formation in cells and is defective in reconstituting methyltransferase activity. Moreover, the lack of any detectable loss-of-function phenotype associated with the TRMT1 p.Gln332Arg variant suggests that the c.995A>G (p.Gln332Arg) allele is non-pathogenic. Altogether, these findings uncover the molecular effects of intellectual disability-associated TRMT1 variants on methyltransferase activity and tRNA binding that underlie deficits in m2,2G modification in human cells.

### TRMT1 variants reveal distinct functional regions required for TRMT1 enzyme activity

To gain insight into the functional effects, we mapped the variants onto a predicted human TRMT1 structure generated through AlphaFold.^44^ The hypothesized structure of human TRMT1 was aligned with the solved structure of Trm1 bound to SAM from the archaea *Pyrococcus horshiki*.^45^ Based upon this structural alignment, human TRMT1 is predicted to fold into two domains coinciding with the SAM-dependent methyltransferase domain and a C-terminal domain unique to Trm1 enzymes (Figure S8, N-terminal domain in blue, C-terminal domain in yellow). The N-terminal domain of TRMT1 forms a putative active site for binding of the SAM methyl donor and a pocket for accommodating the G26 nucleotide that undergoes methylation (Figure S8, red dashed circle denotes active site, and SAM is denoted in green).

Notably, the p.Gln332Arg and p.Arg323Cys variants are situated near the predicted G26 pocket (Figure S8, p.Asp231Asn and p.Arg323Cys). As shown above, the p.Asp231Asn and p.Arg323Cys variants are defective in tRNA modification activity but retain levels of tRNA binding similar to those of WT TRMT1. This result is consistent with these variants perturbing G26 substrate positioning in the active site and preventing catalysis without a major effect on overall tRNA recognition and binding. Similar to the p.Arg323Cys and p.Asp231Asn variants, the p.Arg169Pro variant lies nearby the putative G26 binding pocket of TRMT1. However, in contrast to the p.Arg323Cys and p.Asp231Asn variants, the p.Arg169Pro variant is predicted to disrupt the formation of a conserved α helix within the active site that is likely to cause broader changes in the N-terminal domain. This drastic alteration in structure is consistent with the p.Arg169Pro variant exhibiting defects in both tRNA-modification activity and tRNA binding (Figure 5).

The p.Cys348Arg, p.Leu465_Arg466delinsTrp deletion, and p.Ser467Leu variants lie within the C-terminal domain that is unique to the Trm1 enzyme family. The p.Cys348Arg variant resides within the C1 subdomain. In *Pyrococcus horshiki* Trm1, the C1 subdomain makes numerous hydrophobic contacts with the N-terminal domain.^45^ Thus, the p.Cys348Arg variant could alter the folding of the C1 subdomain, thereby impacting the N-terminal catalytic domain. The p.Leu465_Arg466delinsTrp deletion and p.Ser467Leu variants lie within a predicted alpha helix of the C3 subdomain, which faces across from the active site (Figure S8, C3). The C3 subdomain exhibits similarity with subdomains in phenylalanine tRNA synthetase that bind the anticodon region of tRNA-Phe.^46^ This similarity suggests that the C3 subdomain of TRMT1 could form additional contacts with the tRNA anticodon domain during substrate binding. Consistent with this role, we have found that the p.Leu465_Arg466delinsTrp deletion variant disrupts tRNA binding and reconstitution of tRNA-modification activity. The p.Ser467Leu missense variant might have a milder effect on tRNA binding and TRMT1 enzymatic activity, since it is less drastic a change compared to the p.Leu465_Arg466delinsTrp deletion variant that substitutes two residues in the C3 subdomain helix with a bulky tryptophan residue. Consistent with a milder effect on TRMT1 enzymatic activity, cells expressing the TRMT1 p.Ser467Leu variant in combination with the p.Arg323Cys variant contain more m2,2G modifications than cell lines with complete loss-of-function TRMT1 variants. Altogether, the TRMT1 variants reveal distinct functional activities linked to specific subdomains within TRMT1.

### Depletion of Trmt1 in zebrafish causes behavioral and developmental perturbations

To investigate loss of function *in vivo*, we used zebrafish as a model and employed the CRISPR-Cas9 method to generate bi-allelic mutations in *trmt1* using three gRNAs targeting the functional domain (Table S8). We analyzed the phenotype in the F0 (founder) generation because our previous data suggest that F0 knockouts recapitulate phenotypes from the stable genetic knockouts.^42^^,^^47^ RT-qPCR results found significant downregulation of *trmt1* mRNA expression in F0 knockouts (Figure 6A). We then used LC-MS to measure m2,2G levels in whole larvae or head-only samples from Cas9-injected control and *trmt1* F0 knockout larvae. Consistent with the depletion of Trmt1, the levels of m2,2G modification were reduced in both whole larvae and head-only samples from *trmt1* F0 knockout larvae (Figure 6B). In contrast, no significant difference was detected in the levels of the 1-methyladenosine (m1A) modification, which is another widespread tRNA modification (Figure 6C).

Given that human individuals with pathogenic *TRMT1* variants exhibit behavioral phenotypes, we explored swimming patterns in zebrafish larvae under alternating light and dark conditions. Behavioral assays revealed that *trmt1* mutant larvae displayed increased locomotor activity in both light and dark cycles (Figures S9A–S9C). During dark cycles, the F0 knockouts showed similar activity to controls during the initial 10 min (black bars in Figure S9A, quantified in Figure S9D) but showed sustained higher activity during the subsequent 20 min (green bars in Figure S9A, quantified in Figure S9E), indicative of hyperactivity-like behavior. Additionally, mutants showed a pronounced increase in movement during the first minute of light cycles (Figure S9F), potentially indicating light-induced seizure-like behavior.^42^ Furthermore, *trmt1* knockout larvae exhibited decreased AEBR, suggesting impaired auditory function (Figure S9G).

We next performed morphological phenotyping and found that *trmt1* F0 fish exhibited reduced head, eye, and body sizes (Figures 6D–6G). We further analyzed brain development in F0 knockouts using a reporter line, *Tg*(*olig2*:*dsRed*);*nacre*, which expresses dsRed in oligodendrocytes. Using this reporter line, we found that *trmt1* F0 larvae exhibited a reduction in the size of optic tectum, cerebellum, and hindbrain (Figures 6H–6J). These results show that Trmt1 depletion in zebrafish leads to developmental delays and reduced size of brain regions.

To investigate the heritability and specificity of these phenotypes, *trmt1* F0 fish were raised to sexual maturity and pairwise outcrossed with WT fish. The F1 progeny were genotyped to identify inheritable F0 mutant allele carriers, which were then inbred to produce *trans*-heterozygous (*trmt1*^−/−^) F1 progeny for further analysis (Figure 6K). RT-qPCR confirmed significantly decreased *trmt1* mRNA expression in *trmt1*^−/−^ larvae (Figure 6L).

We examined the tectal neuropil in zebrafish, which is a major visual processing center and plays a crucial role in generating behavior responses in the zebrafish brain. The tectal neuropil contains a dense network of neuronal processes, including axons, dendrites, and synapse within the optic tectum. Immunohistochemistry using anti-acetylated tubulin and anti-synaptic vesicle glycoprotein 2 (SV2) revealed increased distance between tectal neuropils in *trmt1*^−/−^ animals, indicative of significant reductions in brain structure (Figures 6M–6O and S10A–S10C). Moreover, size measurements of different brain regions showed that the habenula, tectal neuropils, and hindbrain exhibited a reduction in area (Figures 6P and S10D). These results reveal that Trmt1 deficiency causes decreased neuronal cell populations and reduced projected neurite formation.

Since our previous *in vitro* studies found that TRMT1-deficient human cells exhibit reduced cell proliferation, we investigated whether this phenotype also manifests *in vivo* and contributes to the neuronal deficits in the brain. Staining with anti-phospho-histone H3, a mitotic marker to identify actively dividing cells in the brain, detected decreased cell proliferation in *trmt1*^−/−^ larvae compared to WT larvae (Figure 6Q). These results indicate that Trmt1 deficiency causes a reduction in neuronal cell proliferation linked to decreased neuronal cell populations. Collectively, these findings show that *trmt1* knockout phenotypes in zebrafish recapitulate a subset of symptoms in humans with bi-allelic *TRMT1* variants, underscoring a conserved role for TRMT1 function in development, neuronal proliferation, and behavior.

### Differentially expressed genes in *trmt1*^−/−^ zebrafish are associated with disrupted cell cycle, immune response, and visual sensing

**Figure 7:**
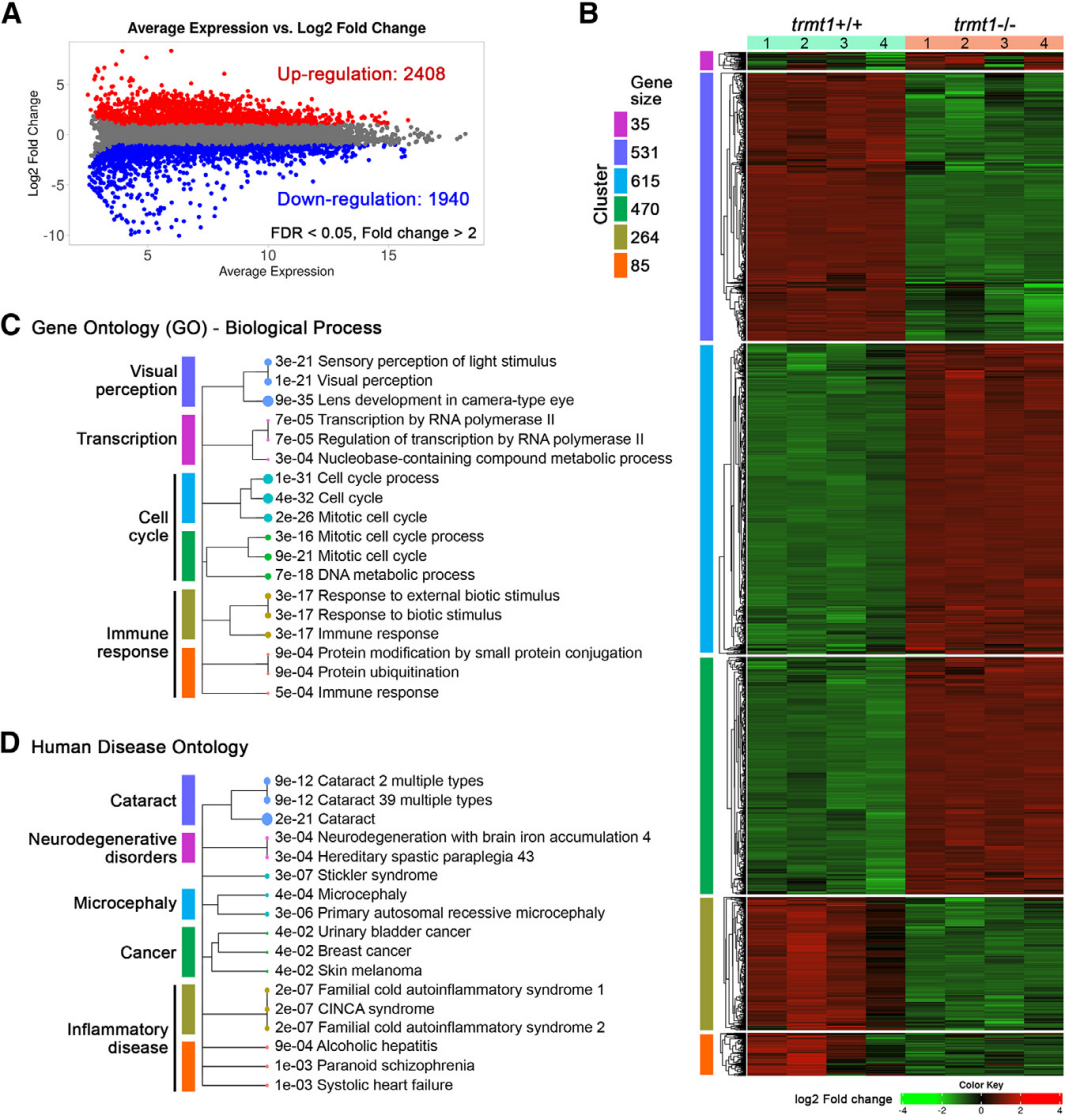
Transcriptomic analysis of Trmt1-depleted zebrafish larvae reveals differential gene expression related to multiple biological processes and human diseases (A) MA plot highlighting significant DEGs with base-2 log fold-change thresholds of ±1 and a false discovery rate (FDR) of <0.05. Red dots represent upregulated DEGs, while blue dots indicate downregulated DEGs. (B) Heatmap of the 2,000 DEGs, clustered using *k*-means based on their SD across all samples. Genes were grouped into six clusters, with the number of genes in each cluster shown at top left. (C) Hierarchical tree of the top three enriched GO biological process terms for each cluster, with FDR values displayed before the corresponding GO terms. (D) Hierarchical tree of the top three enriched Alliance Human Disease Ontology (DO) terms for each cluster, with FDR values placed before the DO terms. See Table S9 for detailed lists.

To explore the molecular mechanisms underlying the phenotypes, we conducted RNA-seq on *trmt1 trans*-heterozygous (−/−) larvae and WT (+/+) controls at 5 dpf. A PCA showed a clear distinction between *trmt1*^−/−^ and *trmt1*^+*/*+^ samples, highlighting significant transcriptomic differences (Figure S11A). The distribution of the transformed data is shown in Figures S11B and S11C. An MA plot, using threshold of fold change >2 and FDR < 0.05, revealed substantial changes in gene expression, with 2,408 upregulated DEGs and 1,940 downregulated DEGs in *trmt1*^−/−^ larvae compared to WT controls (Figure 7A; DEGs listed in Table S9).

We prioritized the top 2,000 DEGs based on their standard deviation and applied *k*-means clustering, visualized through a heatmap, to identify six distinct gene clusters (Figures 7B and S11D). These clusters were analyzed for functional enrichment using gene ontology (GO) and Alliance Human Disease Ontology terms (Figures 7C and 7D; see Table S9 for the full list of analyzed results). GO biological process enrichment revealed that downregulated DEGs are primarily linked to visual perception and immune response, whereas upregulated DEGs are predominantly associated with transcription and cell-cycle regulation. Notably, KEGG pathway analysis of the p53 signaling pathway supported the observation of cell-cycle arrest rather than increased apoptosis (Figure S12). Moreover, these DEGs are implicated in a range of conditions, including vision abnormalities, neurodegenerative disorders, microcephaly, cancers, immune system dysfunctions, and Stickler syndrome; a condition characterized by facial dysmorphisms and vision and hearing problems (Figure 7D and Table S9). In sum, these findings reveal a number of biological pathways and disease pathologies associated with Trmt1 depletion that align with phenotypes reported in human individuals affected by pathogenic *TRMT1* variants.

## Discussion

In this study, we identify and characterize variants in *TRMT1* that impact mRNA splicing, protein levels, and/or enzymatic activity. Our studies define a core set of phenotypic features universally associated with pathogenic *TRMT1* variants that encompasses global developmental delay, intellectual disability, and facial dysmorphism. While no major intrafamilial phenotypic variability was observed, the present cohort exhibited remarkable interfamilial phenotypic variability characterized by a spectrum of behavioral, morphological, and physiological features. These findings are significant by indicating that TRMT1 activity is required for a common set of developmental and neurological pathways with further clinical outcomes determined by genetic and environmental factors specific to each family.

We find that the penetrance of the core phenotypic effects can depend on the severity of the variant on tRNA modification as well as the specific type of change caused by the variant. For example, we find that *TRMT1* variants can induce aberrant splicing, but with distinct outcomes that could differentially impact the functional levels of TRMT1. In addition to loss-of-function splice isoforms, there could be aberrant splice variants that exert dominant negative effects by coding for TRMT1 proteins that bind tRNA substates without modifying them or exhibit gain-of-function properties. It will also be interesting to determine whether any of the splice variants serve regulatory roles that are perturbed by the *TRMT1* variants.

Since TRMT1 is known to modify numerous tRNA targets, each tRNA could be affected to a different extent by a TRMT1 variant that could account for the variable phenotypic outcomes. For example, the compound heterozygous p.Ser467Leu/p.Arg323Cys variant appears to more severely impact the modification of mt-tRNA-Ile compared to cytoplasmic tRNA-Met. In addition, the TRMT1 variants that affect catalytic activity without impacting tRNA binding could retain RNA chaperone functions that are completely abrogated for other TRMT1 variants.^48^^,^^49^^,^^50^ Moreover, there could be additional methylation targets of TRMT1 besides tRNA that would be impacted by pathogenic *TRMT1* variants. Future studies that profile the global modification status and levels of individual RNAs in cells from each family would shed light on the differential effects of each TRMT1 variant.

The variable clinical presentations and age of onset of individuals with similar genotypes support the existence of additional currently unidentified modifying variants in other genes besides *TRMT1*. Future studies will focus on identifying genetic modifiers in this cohort that could reveal the biological pathways and processes that are connected to TRMT1 function. Importantly, the functional demonstration of pathogenicity for so many variants in multiple families across the world indicates that *TRMT1* should be included in genetic registries as a key disease gene linked to developmental brain disorders with autosomal recessive Mendelian inheritance.^51^

Depletion of Trmt1 and m2,2G modifications in zebrafish recapitulates developmental and behavioral phenotypes resembling core features of *TRMT1*-associated phenotypes in human individuals. Specifically, zebrafish *trmt1* knockouts exhibited global developmental delay, reduced brain size, and aberrant behaviors. These results underscore the conserved role of *TRMT1* orthologs in central nervous system development function across vertebrates. Notably, the general reduction in brain size, which mirrors the microcephaly phenotype seen in human syndromes, was observed in both F0 and *trans*-heterozygous knockouts. Furthermore, we observed a significant reduction in cell proliferation in the brains of *trmt1* knockout zebrafish. RNA-seq analysis revealed an upregulation of DEGs involved in the cell cycle, particularly the mitotic phase. This finding aligns with previous *in vitro* studies showing that TRMT1-deficient human cells exhibit slower progression through G_2_/M phase, leading to reduced cellular proliferation.^6^ Overall, our findings support a model in which loss of m2,2G modifications due to a decrease in functional TRMT1 protein and/or activity results in downstream perturbations in molecular and cellular processes that cause neurodevelopmental phenotypes. The future generation of pathogenic variants in zebrafish models will enable a more detailed characterization of the phenotype spectrum in an isogenic background.

## Data and code availability

The data that support the findings of this study are available within the paper and in the supplemental information. Whole-exome sequencing data are not publicly available due to privacy or ethical restrictions. The *TRMT1* variants reported in this paper were submitted to the LOVD database (https://databases.lovd.nl/shared/genes/TRMT1) with the LOVD variant IDs #0000944528, #0000944622, #0000944624, #0000944625, #0000944626, #0000944640, #0000944641, #0000944642, #0000944643, #0000944646, #0000944620, #0000944621, #0000944647, #0000944709, #0000944708, #0000944710, #0000944712, #0000944713, #0000944714, #0000944715, #0000944716, #0000959740, #0000959741, #0000959742, and #0000959743.

## Acknowledgments

The authors thank the affected individuals and their families for their support of this study. One of the authors of this publication (Z.T.) is a member of the European Reference Network on Rare Congenital Malformations and Rare Intellectual Disability, ERN-ITHACA (EU Framework Partnership Agreement ID: 3HP-HP-FPA ERN-01-2016/739516). B.V. is a member of the European Reference Network on Rare Congenital Malformations and Rare Intellectual Disability (ERN-ITHACA) (EU Framework Partnership Agreement ID: 3HP-HP-FPA ERN-01-2016/739516).

The research in this paper was supported by NIH GM141038 to D.F. Studies performed in the lab of G.K.V. was funded by NIH/ORIP R24OD034438. The clinic-genetic research was funded in part by the 10.13039/100010269Wellcome Trust (WT093205MA and WT104033AIA). This study was funded by the 10.13039/501100000265Medical Research Council (MR/S01165X/1, MR/S005021/1, and G0601943), The National Institute for Health Research University College London Hospitals Biomedical Research Centre, 10.13039/501100000833Rosetrees Trust, Ataxia UK, 10.13039/100013128Multiple System Atrophy Trust, Brain Research United Kingdom, Sparks Great Ormond Street Hospital Charity, Muscular Dystrophy United Kingdom (MDUK), 10.13039/100005202Muscular Dystrophy Association (MDA USA), and the 10.13039/501100006282King Baudouin Foundation. S.E. and H.H. were supported by an MRC strategic award to establish an International Centre for Genomic Medicine in Neuromuscular Diseases (ICGNMD) MR/S005021/1. B.V. was supported by the 10.13039/501100001659Deutsche Forschungsgemeinschaft (DFG) DFG VO 2138/7-1 grant 469177153. J.S. is supported by 10.13039/501100000289Cancer Research UK and 10.13039/501100000765University College London. A.F. and S.C. were supported by 10.13039/100012068Health & Care Research Wales, Epilepsy Research UK, and Swansea University PhD funding.

## Author contributions

Conceptualization, S.E., A.E.F., R.M., and D.F.; data curation, S.E., C.D., C.L., K.Z., S.-J.L., R.L., I.K., and D.F.; formal analysis, S.E., C.D., C.L., K.Z., R.L., I.K., D.O., J.S., K.M., B.V., and D.F.; methodology, S.E., A. Scardamaglia, B.V., G.K.V., and D.F.; funding acquisition, B.V., G.K.V., H.H., and D.F.; investigation, all authors; recruitment and clinical and diagnostic evaluations, R.K., F.J., J.R.A., T.S., C.L., M.-L.J., F.T.-M.-T., M.V.-P., R.S., G.Y., M.M.O., J.F., E.H.G., C.P., B.I., C.Petree, C. Phornphutkul, C. Philippe, S.H.K., D.S., V.B., K.P., D.W., M.K.-H., N.R., A.-C.T., H.M., C.F., S.T.B., A.B., N.C., G.L., S.C., Z.T., T.D.H., G.R., T.M., J.R., E.A., M.Z., R.A., H.G., P.N., N.C, M.S.Z., J.G.G., D.G.C., D.P., A.R., I.S.A., G.O., A.E.F., M.B.B., G.B., S.J., J.Z., S.A., G.S., A. Sedaghat, A. Sabri, M.H., S.P., T.A.T., U.A., S.M.B., W.K.C., O.O.G., S.S., H.A.C., G.Z, and P.B.; writing – original draft, S.E., C.D., C.L., K.Z., R.L., I.K., S.-J.L., D.O., J.S., K.M., B.V., and D.F.; writing – review and editing, all authors.

## Declaration of interests

M.M.M. and D.A.C. are employees of and may own stock in GeneDx, LLC. R.S. is on the advisory board of Guide Genetics and Egetis Pharmaceuticals.

## Web resources

gnomAD, https://gnomad.broadinstitute.org/
OMIM, https://www.omim.org/

## Supplemental information

Document S1. Figures S1–S12, Tables S5–S8, Data S1, and a link to access Videos S1 and S2

Table S1. List of the different variants in TRMT1 including testing/methodology information, variant description, allele frequency, and *in silico* predictions and classifications

Table S2. Clinical table of affected individuals with bi-allelic TRMT1 variants Clinical description includes parental consanguinity, family history, ethnicity, birth history, birth weight, birth occipitofrontal circumference, birth length, developmental milestones, motor changes, intellectual disability, behavior, feeding, vision, sleep, abnormal movements, seizures, other medical history, and neurological assessment.

Table S3. Dysmorphology assessment Facial features of the individuals in this study. Individuals within the same family are shaded with the same color.

Table S4. Dysmorphology frequency Matched HPO term and prevalence across individuals in this study.

Table S9. RNA-seq analysis Differentially expressed genes (DEGs), normalized expression, heatmap cluster, gene ontology biological process (GO-BP), and diseases pathway analysis are contained on each sheet.

Document S2. Article plus supplemental information
