# Supplementary material for: Bi-allelic pathogenic variants in TRMT1 disrupt tRNA modification and induce a neurodevelopmental disorder
Source: Am J Hum Genet. 2025 Apr 16;112(5):1117–38. doi: 10.1016/j.ajhg.2025.03.015 (PMC12120178; doi:10.1016/j.ajhg.2025.03.015)
Supplement: Document S1. Figures S1–S12, Tables S5–S8, Data S1, and a link to access Videos S1 and S2 [file mmc1.pdf]

The American Journal of Human Genetics, Volume *112*

## Supplemental information

# Bi-allelic pathogenic variants in *TRMT1* disrupt tRNA modification and induce a neurodevelopmental disorder

**Stephanie Efthymiou, Cailyn P. Leo, Chenghong Deng, Sheng-Jia Lin, Reza Maroofian, Renee Lin, Irem Karagoz, Kejia Zhang, Rauan Kaiyrzhanov, Annarita Scardamaglia, Daniel Owrang, Valentina Turchetti, Friederike Jahnke, Kevin Huang, Cassidy Petree, Anna V. Derrick, Mark I. Rees, Javeria Raza Alvi, Tipu Sultan, Chumei Li, Marie-Line Jacquemont, Frederic Tran-Mau-Them, Maria Valenzuela-Palafoll, Rich Sidlow, Grace Yoon, Michelle M. Morrow, Deanna Alexis Carere, Mary O'Connor, Julie Fleischer, Erica H. Gerkes, Chanika Phornphutkul, Bertrand Isidor, Clotilde Rivier-Ringenbach, Christophe Philippe, Semra Hiz Kurul, Didem Soydemir, Bulent Kara, Deniz Sunnetci-Akkoyunlu, Viktoria Bothe, Konrad Platzer, Dagmar Wieczorek, Margarete Koch-Hogrebe, Nils Rahner, Ann-Charlotte Thuresson, Hans Matsson, Carina Frykholm, Sevcan Tuğ Bozdoğan, Atil Bisgin, Nicolas Chatron, Gaetan Lesca, Sara Cabet, Zeynep Tümer, Tina D. Hjortshøj, Gitte Rønde, Thorsten Marquardt, Janine Reunert, Erum Afzal, Mina Zamani, Reza Azizimalamiri, Hamid Galehdari, Pardis Nourbakhsh, Niloofar Chamanrou, Seo-Kyung Chung, Mohnish Suri, Paul J. Benke, Maha S. Zaki, Joseph G. Gleeson, Daniel G. Calame, Davut Pehlivan, Halil I. Yilmaz, Alper Gezdirici, Aboulfazl Rad, Iman Sabri Abumansour, Gabriela Oprea, Muhammed Burak Bereketoğlu, Guillaume Banneau, Sophie Julia, Jawaher Zeighami, Saeed Ashoori, Gholamreza Shariati, Alireza Sedaghat, Alihossein Sabri, Mohammad Hamid, Sahere Parvas, Tajul Arifin Tajudin, Uzma Abdullah, Shahid Mahmood Baig, Wendy K. Chung, Olga O. Glazunova, Sigaudy Sabine, Huma Arshad Cheema, Giovanni Zifarelli, Peter Bauer, Jai Sidpra, Kshitij Mankad, Barbara Vona, Andrew E. Fry, Gaurav K. Varshney, Henry Houlden, and Dragony Fu**

**Prenatal/Neonatal Course and Presenting Complaints**

The prenatal and neonatal courses were unremarkable in the majority of individuals. However, exceptions include preterm delivery (13%, n=6/38), intrauterine growth restriction with maternal febrile infections during pregnancy (F16-S1), twin loss with the surviving twin requiring neonatal intensive care unit admission due to hypoglycaemia (F15-S1), delayed crying (F2-S2), and preterm birth with renal agenesis, short femora, and polyhydramnios (F4-S1). Additionally, F21-S1 presented with neonatal jaundice and feeding difficulties within the first day, while the affected sibling F21-S2 had feeding difficulties during the initial 10 hours after birth. Fetal distress and fetal heart rate anomalies (F29-S1 and F32-S1) and rapidly resolving bilateral pneumothorax (F31-S1) were also reported. The first concern for the majority of the individuals was the delay or regression of achieved milestones (65%, n=20/31), whereas other presenting issues included seizures (F13-S1, F21-S2, F23-S1, and F23-S2), hypotonia (F5-S1, F12-S1, and F26-S1), dysmorphic features (F12-S1, F24-S1, and F32-S1), prenatal ultrasound findings (F4-S1), slow and insufficient movements (F11-S1), feeding difficulties, failure to thrive, or short stature (F18-S1, F19-S1, and F24-S1), NICU admission (S15, S1), and frequent infections (F19-S1).

**Supplemental Note: case reports**

**Family 1**

This family presents with two affected siblings. The index case (F1:S1) is a 17-year-old male born full- term following induced ventouse delivery to consanguineous healthy Irish traveller parents. His neonatal period was unremarkable with normal birth weight (2.93 kg, -1.06 SD), height and occipitofrontal circumference. He presented with delayed speech and motor developmental milestones. By 2 years of age, he could only speak single words and at 7.5 years of age he could count 1-10 and has derangement of speech. He presented with stiff unsteady gait, jerky movements, frequent stumbling at 5 years of age and he could not peddle on bike at 7 years of age. At 5 years of age he was around 2 years delayed with fine motor development and at 7.5 years of age he had difficulty with pincer grip and threading beads. He was unable to write and could only read a few words. At 14 years of age he could read simple books, however writing a challenge, he presented with poor concentration, was very active and hence attended a special needs school. He presents with moderate intellectual disability. An important neurological symptom was complex motor stereotypies and tics (flaps hands when excited, licks hands, rubs hands on head, extends neck, extends arms behind back), verbal tics, frequent jerks and unusual posturing of arms (onset at 5 years of age). Physical examination at 14 years and 4 months of age revealed normal OFC (53.5 cm, -0.74 z). Growth parameters at 7.5 years of age were height at 117.4 cm (-1.24 SD) and weight at 22 kg (-0.76 SD) and OFC at 14 years and 4 months was at 53.5 cm (-0.78 SD). He presents with facial features such as broad eyebrows, epicanthic folds, telencanthus, prominent ears, thick upper ear helix and wide-spaced teeth, but also hand and foot deformities such as 5th finger clinodactyly and camptodactyly, single palmer creases. Brain MRI was also normal. There is no epilepsy however, he displayed eye convergence during tic-like behavior and EEG at 5 years of age was abnormal with bursts of high amplitude generalized 2-3 Hz spike and wave activity in both awake and sleep states (photosensitive). Video telemetry showed discharges on EEG persisted but did not coincide with any of his jerky movements or tics.

His affected 12-year-old sister was born full- term following normal delivery. Her neonatal period was unremarkable with normal birth weight (2.75 kg, -1.29 SD), height and occipitofrontal circumference. She also presented with delayed speech and motor developmental milestones at 2 years of age. However, her delay was milder to her brother's, and she could speak single words at 4 years and 10 months of age. She presents with mild intellectual disability. She presented with ADHD at 11 years of age and needs one-on-one help at school. She has intermittent vision with a convergent squint, and she is quite restless when asleep. On neurological examination she presents with some fidgety movements, however no motor stereotypies. She presents with facial features such as Epicanthic folds, mild telencanthus, diastema but also hand and feet deformities such as Incurved 5th toes, normal creases, hypoplastic 5th toenail left foot. Brain MRI was also normal. Physical

examination at 4 years and 10 months of age revealed a normal OFC (49.5 cm, -0.45 SD) and growth parameters were height at 102.6 cm (-1.51z) and weight at 15.2 kg (-1.44z). EEG at 5 years of age was abnormal with several bursts of generalized spike and slow wave activity one of which was seen during hyperventilation.

Video S1. Individual 1.1 from family 1, available using link below
Video S2. Individual 1.2 from family 1, available using link below

https://www.ebi.ac.uk/biostudies/studies/S-BSST1486?key=20082a02-2ab5-420b-8b36-85c2a4848101

**Family 2**

This family presents with two affected siblings. The index case (F2:S1) is a 9-year-old male born full-term following SVD delivery to non-consanguineous healthy Pakistani parents. His neonatal period was unremarkable with normal birth weight, height and occipitofrontal circumference. He presented with delayed speech and motor developmental milestones. By 4 years of age, he could only speak single words and at 9 years of age he could form 2-3 word sentences. He achieved sitting at 1 year old followed by walking at 3 years old and is currently ambulatory in all settings. He has an impaired difficulty in understanding simple tasks. At 6 months of age, he developed febrile seizures (4-5 episodes in total) with uprolling of eyes, cyanosis and loss of body tone during the seizure episode. At the last review at 5.5 years old, he was hyperactive ang aggressive and presented with repetition of words (echolalia). On neurological examination, he presented with brisk reflexes. MRI imaging at 3 years of age showed T2W periventricular & peritrigonal hyperintensities and white matter changes suggestive of ischemic demyelination.

His affected 8-year-old sister was born full- term following SVD delivery. Her neonatal period was unremarkable with normal birth weight, height and occipitofrontal circumference. She also presented with delayed speech and motor developmental milestones. She could speak a single word at 1 year old and currently only says 4-5 words. She presents with mild intellectual disability. At 2 years of age, she developed an episode of generalized, tonic, clonic seizures and uprolling of eyes. She started on levetiracetam and has been seizure free for 1.5 years. On neurological examination she presented with brisk reflexes. MRI imaging at 2 years of age showed T2W periventricular & peritrigonal hyperintensities and white matter changes suggestive of ischemic demyelination.

**Family 3**

This family presents with three affected siblings. The index case (F3:S1) is a 24-year-old female born full term following NVD delivery to consanguineous healthy Mennonite parents. Her neonatal period was unremarkable with normal birth weight (3.01 kg, -0.79 SD). She presented with global developmental delay, particularly speech delay at around 2 years of age. By 4 years of age, she could speak but had derangement of speech. She presents with gross motor delay, poor coordination, mild ataxia and her fine motor skills are mildly delayed. She presents with moderate intellectual disability and attends an individual education plan with education assistance. On neurological examination she presents with brisk reflexes, poor coordination, mild dysmetria, mild dysrhythmia. Her current growth parameters include height at 163.5 cm +0.04 SD), weight at 76.4 kg +1.16 SD) and OFC at 54 cm -0.29 SD). MRI imaging was normal.

Her affected 28-years-old brother was born full term following NVD delivery. He follows a similar clinical picture with the additional presentation of seizures over a decade ago which have been controlled with anticonvulsant medication. His current growth parameters include height at 171.4 cm (-0.72 SD), weight at 71.7 kg (+0.16 SD) and OFC at 56.5 cm (+0.62 SD).

Their also affected 30-years-old brother was born full term following NVD delivery. He follows a similar clinical picture with the additional presentation of increased range of motion of the joints. His current growth parameters include height at 171.6 cm (-0.70 SD), weight at 62 kg (-0.84 SD)and OFC at 58 cm (+2.02 SD).

**Family 4**

The index case (F4:S1) is a 14-year-old male born at 35 weeks following induced delivery to non-consanguineous healthy Creole parents. The first concerns were during prenatal period due to unilateral renal agenesis, short femora and polyhydramnios. He had low birth parameters with weight at 2.05 kg (-1.32 SD), height at 41 cm -2.27 SD) and occipitofrontal circumference at 31 cm (-0.87 SD). He has never achieved walking or crawling. He presents with moderate intellectual disability and attends a specialized school. He presents with speech delay, autism and echolalia. He presents with facial features such as broad eyebrows, lower lid everted, malar hypoplasia, anteverted nares, long philtrum, prominent ears, narrow mouth, crowded teeth but also equine of the feet. Brain MRI and EEG studies were normal. His growth parameters at 12 years of age include height at 109.5 cm (<1p, -5.42 SD) and weight at 18.7 kg (-3.66 SD) while OFC at 9 years of age was 49 cm (-3.22). Other medical history include asthma, multiple fractures, osteoporosis, unilateral renal agenesis, macrophage activation syndrome.

**Family 5**

The index case (F5:S1) is an 8-year-old male born at full term with normal delivery to healthy non-consanguineous Spanish parents. His neonatal period was unremarkable with normal birth weight (3.40 kg, (-0.26 SD), height (50 cm, -0.06 SD) and occipitofrontal circumference (35 cm (-0.40 SD)). At 6 months of age, he first presented with hypotonia and then by the age of 3 years with global developmental delay which includes intellectual disability, speech and motor delay. She presented with global developmental delay, particularly speech delay at around 2 years of age. By 4 years of age, he could only speak about 10-20 words. On neurological examination he presents with motor stereotypies, hand flapping and tics. At 3.5 years of age, he developed febrile seizures and had an abnormal EEG. He presents with constantly open mouth and high palate. MRI imaging at 3 years and 10 months of age showed corpus callosum dysgenesis. Cerebellar vermis hypoplasia. His growth parameters at 8 years old include height at 104 cm (+0.77 SD), weight at 18 kg (+0.94 SD) and OFC at 50.5 cm (+0.13 SD).

**Family 6**

The index case (F6:S1) is an 8-year-old male born at full term with Cesarean section to healthy non-consanguineous Hispanic parents. His neonatal period was unremarkable with normal birth weight (3.30 kg, -0.42 SD). He has a sister with *KCNN2*-related NDD. At 2 years of age, he first presented with delayed speech and motor development. He has been attending speech and physical therapy since 2 years old as well as special education since 4 years old. He presents with mild intellectual disability and ADHD. Early on, he also developed localization-specific seizures and was weaned off Keppra. His EEG showed mild background slowing-no more focal epileptiform discharges. He presents with facial features such as occipital prominence, slight upward slant to palpebral fissures, epicanthal folds, malar hypoplasia, full cheeks and thick helices and thin upper vermillion. His hands show hockey stick creases. MRI imaging showed partial empty sella with intact pituitary tissue and bright spot. His growth parameters at 8 years old include height at 126.2 cm (-0.29 SD), weight at 31.7 kg (+0.99 SD) and OFC at 51 cm (-0.94 SD). Other medical history includes undescended testicles bilaterally.

**Family 7**

The index case (F7:S1) is a 6-year-old male born full term with Caesarean section to non-consanguineous healthy parents of German, Irish, African, Scottish and Scandinavian origin. The

father's maternal half-brother has a learning disability. His neonatal period was unremarkable with normal birth weight (4.110 kg, +1.10 SD) and height (55.88 cm, +2.18 SD). He was able to walk at 10 months of age. He presents with speech regression and non-verbal autism (level 3, requiring very substantial support) with poor eye contact. He presents with developmental delays, obesity and sleep apnea (on continuous positive airway pressure). He will respond to his name but does not say any words. He attends special Education kindergarten, speech and occupations therapies. A Child Development Inventory done at 2 years and 2 months of age noted Social age: 14 months, Self-help: 17 months, Gross motor age: 21 months, Fine motor age: 2 years, Expression language: 12.5 months, Language Comprehension: 12 months, General development: 17.5 months.

At 5 years of age, he finger feeds himself but does not use utensils. He is very restrictive in his food choices. His mother also states that he chokes on some foods and will vomit when he cries sometimes. Socially, he does not interact well with others, he hits himself if he is angry, and has decreased pain reaction. On neurological examination, he presents with an abnormal movement which includes hand flapping, back and forth moving and spinning when excited or angry. Physical examination at 5 years of age current age revealed macrocephaly (58cm, +4.87 SD) height at 134 cm (+5.45 SD) and weight at >6 *SD*. His face is also brachycephalic. He has no history consistent with seizures, but the family did not complete the EEG which was recommended. He has not had a brain MRI either. He had a normal chromosomal microarray.

**Family 8**

The index case (F8:S1) is a 19-year-old male born at 37+3 with Caesarean section to non-consanguineous healthy parents of Dutch origin. Family history is remarkable for a brother who exhibits intellectual disability and similar facial dysmorphisms (though not tested yet) and a sister who has mild intellectual disability and epilepsy and is a carrier for c.389_390delAA, p.(Lys130fs) in *TRMT1*. At birth, he was found to have low weight (2.24 kg, -2.11 SD) and had a height of 46 cm (-1.56 SD). He first presented with global developmental delay and regression of achieved milestones. He began having seizures at 1 year old and experienced status epilepticus at 15 months of age, after which his seizures are well-managed on anti-seizure medications. An EEG after status epilepticus was consistent with too slow, non-differentiated encephalopathic EEG with no epileptic activities (that comprised of post-ictal/ post-anoxic/ metabolic encephalopathy). In terms of motor milestones, he started walking before 15 months old, however he regressed after experiencing status epilepticus at that age and independent walking was never achieved.

An MRI at 2 years old showed frontotemporal atrophy with corresponding ventriculomegaly, uniform thinning of the corpus callosum secondary to ventricular enlargement, midbrain atrophy in keeping with supratentorial atrophy, mega cisterna magna and posterior plagiocephaly.

On neurological examination at 19 years of age, he presented with hypertonicity in the lower limbs and had poor coordination and severe *pes equinus*. He is in a wheelchair and can only walk with assistance for a short distance. There is a severe delay in speech, he can speak in few single words and attends a special needs school. He presents with dysmorphic features including broad and full arched eyebrows, mild telecanthus, mild blepharophimosis, mild upslant, deepset eyes, deep nasal bridge, full nasal tip, thin upper lip, prominent and broad mandibula and chin, relatively short and broad fingers with short distal phalanges and *pes equinus*. His growth parameters at 19 years old include height at 177 cm (+0.06 SD) and OFC at 16 years of age at 53.4 cm (-1.41 SD). He is also overweight.

**Family 9**

The index case (F9:S1) is a 37-year-old female born at term through NVD to a potentially consanguineous family in Puerto Rico. Maternal great grandfather and paternal great grandfather were thought to be cousins, though the degree of relationship is unknown. At birth, her weight was 3.17 kg (-0.62 SD) and height was 53.43 cm (+1.44 SD). She first presented with global developmental delay

at around the age of 18 months. Regarding gross motor skills, she achieved sitting 8 months and began walking at 2 years. Additionally, there were difficulties with fine motor skills as she cannot write. She had a delay in language development, speaking her first words after the age of two. She demonstrated aggressive behavior and attended a special education programme. She has a history of seizures, frequent migraine attacks, sensorineural hearing loss, anxiety and hypertension. Family history is unremarkable for seizures and intellectual disability. Her examination at follow up was remarkable for dysmorphic features including broad eyebrows and brachydactyly. She was overweight with a BMI of 25.7. Brain MRI imaging was unremarkable.

**Family 10**

The index case (F10:S1) is a 6-year-old male born at term with normal delivery to healthy non-consanguineous Caucasian parents. At birth, he weighed 3.77 kg (+0.43 SD), measured 52 cm (+0.70 SD) in height, and had an occipitofrontal circumference of 35 cm (-0.40 SD*)*. His developmental milestones indicate that he began walking at 18 months. His language development was delayed, he spoke his first word at 3 years and at 5 years of age he could speak a few words. Learning difficulties and hyperactivity were observed in his early years. Notably, he experienced three episodes of generalized febrile seizures starting at the age of 2. His examination at follow up was remarkable for dysmorphisms, including a large forehead, prominent eyebrows, and large low-set ears as well as mild hypertrichosis on legs. His growth measurements were height at 104 cm (-1.27 SD), weight at 18 kg (-0.40 SD) and OFC at 52cm (+0.64 SD). An MRI conducted was unremarkable.

**Family 11**

The index case (F11:S1) is a 14-year-old male born at term in Turkey to a consanguineous family. He had an uneventful perinatal course and was born with low weight (2.35 kg, -2.50 SD) and normal height (50 cm, -0.06 SD) for gestational age. Insufficient and slow movements and not crying when hungry were immediately notable in the neonatal period. Developmentally, he began walking at 3 years old and had a delay in language development. He started speaking single words after the age of 8 and his language skills remain severely limited, now at the age of 13 years and 4 months he can speak with 20-30 single words and rarely constructs short two-word sentences in the last 6-7 months. Learning difficulties were evident, as he could not read or write. Despite attending a special education class, he shows minimal progress. He has intellectual disability; he only knows simple commands and forgets them in a short time. Additionally, he was unable to learn how to read and write. Behaviorally, he is notably calm and does not communicate pain if hurt. He is constantly eating, eating uncontrollably even when full. Two episodes of febrile seizures occurred at 18 months and 2 years of age, an EEG conducted at 18 months was unremarkable. He was put on Valproic acid from 2 to 4 years old and was seizure-free after the age of 4. Other past medical history includes vision loss in his left eye is accompanied by a suggestion for glasses, which he cannot use. Irregular sleep patterns persist, with only 4-5 hours of sleep at night and stereotypic behavior of waking up, opening, and closing doors.

Moderate hearing loss is present in the right ear, and mild in the left. There is mild aortic insufficiency, though the last echocardiography was normal. Additionally, there is a family history of a maternal cousin who has myopathy.

His examination at follow-up was notable for dysmorphic features including small eyeballs, a narrow and long face, prominent antihelix bilaterally, a groove in the columella, a narrow and high palate, and arachnodactyly in both hands and feet. Growth parameters revealed microcephaly (OFC 50.5 cm, -2.51 SD), normal weight (46 kg, -0.05 SD) and height (161 cm, +0.48 SD). Neurological examination showed brisk deep tendon reflexes without upper motor neuron signs. Movements were prominently slow and he had walking difficulties due to balance problems. Unpurposeful arm and leg movements were noted. No significant verbal communication could be performed, but he could follow some simple commands such as opening his mouth. Brain MRI imaging done at 8 years of age revealed a dilated and asymmetric ventricular system, anterior thickening and posterior thinning of the

corpus callosum, deep white matter atrophy with corresponding ventriculomegaly, and cerebellar atrophy.

**Family 12**

The index case (F12:S1) is a 5-year-old male born at full term to a Kurdish consanguineous family with an uneventful prenatal history. At birth, he weighed 2.72 kg (-1.32 z), measured 51 cm (+ 0.59z) in height, and had an OFC of 35 cm (+0.42z). He presented with developmental delay, severe hypotonia and trigonocephaly. He had a global developmental delay failing to meet developmental milestones in multiple areas of functioning. He had moderately delayed gross motor milestones with walking at 25 months and mild delays in fine motor milestones. Speech development was also delayed, first words at 17 months and two-word sentences at 2 years and 9 months and at 4 years 4 months his vocabulary was limited to only 50 words. Mild intellectual disability became evident, coupled with challenges in concentration. His physical examination at 2 years and 9 months revealed microcephaly (46.5 cm, -3.2 SD), height at 92 cm (-0.41 SD) and weight at 11.5 kg (-1.77 SD). He had ataxic gait and was able to speak two words.

His last follow up examination at 4.4 years was remarkable for prominent metopic suture, narrow and upslanting palpebral fissures, flat midface, hypoplastic alae nasi, an open mouth appearance, a small chin, and small ears. Neurological examination was notable for hypotonia, clumsiness, poor coordination and poor concentration. There was delayed speech, he could use 50 active words and echolalia was noted. An MRI at 4 months was notable for slightly delayed myelinization and enlarged inner and outer ventricles, while EEG was unremarkable. He additionally carries a *de-novo* microdeletion of 15q13.

**Family 13**

The index case (F13:S1) is a 13-year-old female born at term through NVD to consanguineous Turkish parents. She had an uneventful perinatal course and was born with weight at 2.6 kg (-2.71 SD), height of 47 cm (-1.10 SD) and OFC at 31 cm (-2.58 SD). She first presented with febrile seizures at the age of 9.5 months which continued until 1 year of age. She then presented with motor delay (walked at around 18 months of age) and speech delay (spoke around 5 words at around 30 months). She has moderate intellectual disability and hearing impairment, thus she attends state special school for the deaf. She was diagnosed with sensorineural hearing loss and wears cochlear implants. She is quite an anxious child, often spills foods and is quite restless while asleep. She has a short stature and also presents with hyperopia. She is hypertonic in the lower extremities. Her physical examination revealed dysmorphic features that include low front hairline, bushy eyebrows, upslanting palpebral fissures, depressed nasal bridge, full lips, flat midface. At 12.9 years of age, her growth parameters were height at 145 cm (-1.33 SD), weight at 45.3 kg (+0.10 SD) and OFC at 53.3 cm (-0.03 SD). An EEG and MRI carried out at 8 years of age were both normal.

**Family 14**

The index case (F14:S1) is a 7.5-year-old male born via Caesarean section at term. He is from non-consanguineous Swedish/Caucasian parents, however, his maternal and paternal grandmothers are both from the same village. Birth weight was 3.6 kg (+0.08 SD), height was 52 cm (+0.70 SD) and OFC was 34.5 cm (-0.61 SD). There is a history of seizures on the maternal side of the family. As a teenager, the mother had a few seizures in conjunction with lack of sleep. A cousin of the mother presented with seizures at the age of 10 years while another cousin diagnosed himself with ADHD and seizures as a teenager, has a son with the sister of the proband's father, who presented with hydrocephalus, developmental delay and language impairment.

Developmental delays were first noted at 6 months and then at the age of 10 months, he presented with febrile seizures, at 1.5 years seizures were afebrile, generalized tonic-clonic and were treated

with levetiracetam. At the age of 2, he started speaking and walking. At 4 years and 11 months old, his height was 106.4 cm (-0.32 SD), weight at 16.4 kg (-0.89 SD) and OFC at 50.4 cm (-0.37 SD). At that age, he presented with delayed gross motor development with broad based and imbalanced gait and frequent stumbling. He could only speak in single words and occasionally could construct two-word sentences. He had slender built, had small hands and feet and pes planus. Additionally, he had astigmatism and unilateral strabismus.

At the follow up examination at 7.5 years, his growth parameters were height at 120 cm (-0.76 SD) and weight at 20 kg (-1.48 SD). He could speak with two-word sentences. A diagnosis of moderate ID was established. He was very active and sociable and was enrolled in special education. Brain MRI at age 2 was unremarkable. EEG at 2.5 years of age revealed right sided frontal epileptogenic activity during sleep and mild general background slowing. Repeated EEG at 6 years of age revealed no epileptogenic activity but the same slowing of background activity was present.

**Family 15**

This family presents with two affected siblings. The index case (F15:S1) is a 4-year-and-10-month old male born via Caesarean section to consanguineous Turkish parents. The pregnancy was complicated by maternal gestational diabetes, and it was an IVF twin pregnancy with the loss of one twin at 6 weeks. Born at 36 weeks, he weighed 2.4 kg (-1.01 SD) and measured 48 cm (+0.11 SD)in height. He was in NICU for 11 days due to hypoglycemia and subsequent hospitalization at 2 months due to pneumonia and eczema. In terms of speech, he started speaking at 2 years old, but his language skills are severely limited at 3 years and 3 months with speaking in single words. Walking started at 3 years, marked by clumsiness and difficulty, and he faces challenges with fine motor skills such as stacking cubes. Moderate intellectual disability is present, and he exhibits sociable behavior but also shows signs of hyperactivity and self-mutilation under stress. His eating habits are highly restrictive, preferring crunchy foods and facing allergies to several food items. Irregular night sleep, including episodes of waking due to shortness of breath and apnea attacks, is noted. Itching upon removing his top and vocal tics before sleep are additional nighttime challenges. Other past medical history includes lathosterolosis (hepatosplenomegaly, oedema of the lower legs, adrenal insufficiency, cholestasis, jaundice, hypoalbuminemia, hypocholesterolemia and hypertriglyceridemia), common variable immunodeficiency syndrome for which he receives IVIG, anemia and thrombocytopenia due to chronic ITP, asthma and atopic constitution. The family history indicates one brother and two monozygotic uncles on the maternal side have the same intellectual disability and facial dysmorphic features, however, they have not been genetically tested yet.

At the follow-up visit at three years and three months of age, his OFC was at 44 cm (-3.26 SD), height at 90.3 cm (-1.77 SD) and weight at 14 kg (-0.51 SD). At 4 years and 10 months, he had microcephaly (OFC at 48 cm, -2.03 SD), he measured 104 cm (-0.73 SD) in height and 19 kg (+0.38 SD) in weight. On examination, several dysmorphic features were noted including microcephaly, broad nasal bridge, epicanthus inversus, upslanted palpebral fissures, full nasal tip, sparse eyebrows, sparse hair, micrognathia (pointed chin), retrognathia, thin upper lips and high palate. He also presents with tooth decays, petechiae on the palate due to low platelet count, brachydactyly, toe 2-3 finger partial syndactyly, and a sandal gap was also observed. On neurological examination, hypotonia and difficulty in walking were evident. MRI findings at 2 years of age indicate cerebellar atrophy, cerebral cortical atrophy, triventriculomegaly, and mild hypoplasia of the adenohypophysis. Although not clinically diagnosed, his EEG at 3 years of age, shows irregularities not typical for his age, with high-amplitude sharp wave activity in the fronto-centro-temporal region and mild background rhythm irregularity suggest an inactive epileptiform anomaly originating from cortical tissues.

His affected 20-year-old brother (F15:S2) was born term following a normal delivery. His birth height and weight were unremarkable (55 cm, +1.84 SD and 3.5 kg, -0.10 SD, respectively). He presented at 2 years old with delayed speech and motor development. He started speaking his first words at 2 years old and started forming meaningful and long sentences at 7 years old. He achieved independent

ambulation at 2 years old; however, due to hypotonia his gait was unsteady. His balance improved at 10 years old. At his current age at 20, he has mild ataxia, poor coordination, able to do simple chores, but unable to paddle a bike. He can hold a pencil, write letters and draw a stickman. He cannot read or write sentences. Intellectually, he has mild to moderate disability. He used to be hyperactive until 10 years of age, now he is very calm and his restrictive food choices resolved, he used to only consume eggs and yoghurt. Generalized tonic-clonic seizures were first noted at the age of 8. At that time, EEG findings revealed that although physiological elements of sleep were present, their maturation was not consistent with the individual's age. High-amplitude sharp wave activity was observed multiple times in the fronto-centro-temporal region. Additionally, the EEG demonstrated mild irregularity in the background rhythm of cerebral bioelectric activity, which was also incompatible with the individual's age, alongside inactive epileptiform anomalies originating from cortical tissues. Seizures remained well-controlled with treatment until the age of 12, after which the treatment was discontinued, and the individual has remained seizure-free since then. An MRI brain at 12 years old revealed mild cerebellar atrophy, mild cerebral cortical atrophy, and mild ventriculomegaly.

On last follow-up at 20 years, he had mild microcephaly with an OFC at 54 cm (-2.47 SD), he measured 175 cm (-0.19 SD) in height and weighed 55 kg (-2.03 SD). Examination revealed broad nasal bridge, epicanthus inversus, upslanted palpebral fissures, full nasal tip, sparse eyebrows, sparse hair, micrognathia, high palate, multiple tooth decay and sandal gap. Neurological examination was remarkable for hypotonia with normal deep tendon reflexes and ataxia.

**Family 16**

The index case (F16:S1) is an 8-year-old male born term via Caesarean section to consanguineous Turkish first-cousin parents. There is family history that includes motor disorder in a first cousin's son. Prenatal history was significant for intrauterine growth retardation and an infection. Birth weight was 2.25 kg (-2.10 SD), height was 43.5 cm (-2.49 SD), and OFC was 31 cm (-2.15 SD). Developmental delay was obvious from the first months of life. He could sit at 9 months and walk at 2 years of age, but started speaking at 3 years. A global developmental delay was confirmed through the Brunet Lezine test, showing an age equivalent of 20 months at 5 years and 8 months chronological age. At the age of 8 years, he had severe intellectual disability, with no language, limited verbal comprehension out of context, and a lack of autonomy. He attended specialized school, struggling with basic daily tasks, and requiring assistance for eating and dressing.

His medical history includes febrile seizures starting at 2 years old, which evolved to secondary focal seizures and became seizure-free at 4 years old with a combination of valproic acid and levetiracetam, with ongoing treatment. EEG findings include a poor background rhythm and a few abnormal waves. On follow up examination, he was clumsy and had spastic diplegia with bilateral epileptic tremors, predominantly distal and affecting mainly the left leg, has resulted in secondary orthopedic deformities. Reflexes were brisk. Growth parameters were weight at 22 kg (-1.22 SD), height at 119 cm (-1.57 SD), and OFC at 47 cm (-3.83 SD). MRI imaging at 3 years of age was normal, but subsequent scans at 4 and 6 years of age revealed a left anterior middle cranial fossa arachnoid cyst, posterior thinning of the corpus callosum, and right hemispheric cerebellar atrophy.

**Family 17**

The index case (F17:S1) is an 11-year-old female born full-term to consanguineous Pakistani parents after an uneventful prenatal period. Her birth parameters were weight at 2.80 kg (-1.19 SD) and height at 52 cm (+0.93 SD). She presented with delayed gross motor skill development, where she never crawled and walked at the age of 2 years. She also had delays in speech development; she had small sentences at 3 years old and currently, at the age of 11 years, she knows the alphabet, but connection to words and spelling is difficult. According to her father, she is stagnant and needs special help at school. Writing is difficult for her due to restlessness and fine motor development delay. At school she needs teachers' instructions and guidance and works best in small groups. She knows the alphabet, but the connection to words and spelling is difficult. She knows numbers, but the addition of

numbers above 15 is difficult. She is restless, impulsive, easily distracted and has a short attention span and is diagnosed with moderate ADHD. Cerebral MRI imaging done at 3 years and 4 months of age showed multiple calcifications in frontal and temporal lobe on the right hemisphere. An EEG was unremarkable. On physical examination, she has small skin protrusions on her hands, fallen feet and smaller ear auricular lobules than those of her parents. Growth measurements showed a height of 149.3 cm (+0.73 SD), a weight of 41 kg (+0.34 SD) and an OFC of 52 cm (-0.49 SD).

**Family 18**

The index case (F18:S1) is a 6-year-old female born full term to non-consanguineous Hispanic parents with an uneventful prenatal history. Her birth parameters were weight at 2.778 kg (-1.23 SD) and height at 49.5 cm (+0.02 SD). She first presented with early poor growth and weight gain, then her motor development was delayed with clumsy walking. Her speech is delayed and currently she says only a few words and does not use sentences. She exhibits moderate intellectual disability and needs extra help in school. On neurological examination, she presents with poor coordination and experiences frequently falls. She is a picky eater and restless sleeper. She presents with myopia as well as esotropia, for which she had surgery. She has experienced multiple allergies, which include bronchospasm (under therapy) and middle ear infections, which has caused decreased hearing. On physical examination, she has a prominent forehead. Her current growth parameters include height at 117 cm (+0.43 SD), weight at 20.1 kg (-0.06 SD) and OFC at 51 cm (+0.14 SD).

**Family 19**

The proband (F19:S1) is a 2-year-8-month old female born term to non-consanguineous Caucasian parents. At birth, she had an OFC of 34 cm (-0.54 SD), weighed 3.53 kg (+0.23 SD) and her height was 53 cm (+1.29 SD). She presented with delayed achievement of developmental milestones, feeding problems and frequent respiratory infections. There was a delay in her motor development with achieving unsupported sitting at 18 months and she has currently not achieved independent walking at the age of 2 years and 8 months. She spoke her first at 18 months and can use six different words at current age. She has mild intellectual disability and exhibits no behavioral issues. She is fed via PEG to address the feeding issues. Other medical history included strabismus convergens alterans, hepatopathy, liver fibrosis, liver transplanted at age 2 years and 1 month, impaired hearing, thrombocytopenia and arterial hypertension.

At the follow up at 2 years and 8 months, she weighed 13 kg (-0.16 SD), her height was 86 cm (-1.55 SD) and OFC was 45 cm (-2.12 SD). Neurological examination showed hypotonia. An MRI brain and EEG was unremarkable.

**Family 20**

The proband (F20:S1) was a 4-year-old boy born via c-section to a consanguineous family from Pakistan. His birth weight was 2.4 kg (-2.50 SD). He presented with global developmental delay. His motor development was delayed with sitting at 10 months and walking independently at 2 years of age. He spoke his first word at 2 years and had moderate ID. He started having seizures at 1 years which are flexor spasm and febrile in nature and well-controlled with carbamazepine. He is fit-free for six months. At follow up at 4 years old, he had an OFC of 47 cm (-2.27 SD) weight of 16 kg (-0.14 SD) and height of 99 cm (-0.76 SD). An MRI brain imaging showed diffuse abnormal signal area involving bilateral peritrigonal white matter with areas of hypomyelination, possibly of periventricular leukomalacia, whereas an EEG at 3.5 years of age showed focal epileptogenic activity arising from right hemisphere, occipital, parietal and temporal area.

His affected 2-year-and-2-month-old brother was born with a weight of 2.7 kg (-1.38 SD ) and had developmental delay with sitting at 9 months, walking at 1 year and 9 months old and speaking his first words at 15 months. He had moderate ID. Similarly to his brother, he developed seizures at 1 month of age and his seizures are well-controlled on carbamazepine and he has been fit-free for 1.5

years. At the follow up, he had microcephaly, weighed 11.5 kg (-0.96 SD) and measured 83 cm (-1.21 SD).

**Family 21**

This family presents with two affected siblings born to double first cousin Iranian Arab parents. The family history is remarkable for an affected uncle and aunt.

The proband (F21:S1) is a 19-year-old female born preterm(4 weeks earlier than due date). Her birth measurements were OFC of 33.5 cm (+0.62 SD), weight of 2.48 kg (-0.55 SD) ,and height of 46 cm (-0.4 SD). Initial concerns arose within the first 24 hours of life due to neonatal jaundice and poor feeding. Profound congenital deafness was also identified early. Her gross motor skills are clumsy with achieving independent sitting at 9 months and ambulation at 15 months, and fine motor skills are abnormal, with intention tremor noted. She has moderate intellectual disability and absent speech at her current age. Behaviorally, she is reported to have a bad temper and did not attend school. Seizures of an unknown type began at 27 months, though an EEG performed at the time was unremarkable. Vision, sleep, and feeding have remained unremarkable. At her last follow-up at 19 years old, her growth parameters, including height, weight, and OFC, were within normal ranges. Physical findings included bilateral fingertip skin stiffening and bilateral flat feet. Neurological examination revealed a clumsy gait and decreased deep tendon reflexes. Brain MRI findings indicated notable anomalies in the centrum semiovale and global white matter volume loss.

The affected sibling (F21:S2) is a 17-year-old female born at term via normal vaginal delivery. Concerns arose within the first 10 hours of life due to poor feeding. Her birth measurements were not concerning, with a weight of 2.95 kg (-0.90 SD), height of 52 cm (+0.93 SD), and occipitofrontal circumference (OFC) of 33 cm (-1.22 SD). She exhibited clumsy gross motor skills, achieving independent sitting at 8 months and ambulation at 12 months. Fine motor skills are abnormal, with intention tremor noted. Seizures began at 25 months, which prompted medical attention. She has mild to moderate intellectual disability and stuttering. She currently attends the third grade at a special needs primary school and is reported to have a bad temper. Vision, sleep, and feeding have remained unremarkable. At her most recent follow-up at 17 years old, her growth parameters, including height, weight, and OFC, were within normal ranges. Physical examination revealed bilateral fingertip skin stiffening and bilateral flat feet. Neurological examination showed a clumsy gait and decreased deep tendon reflexes. An EEG performed at the time was unremarkable.

**Family 22**

The index case (F22:S1) is a 2.5-year-old female born at full term via Caesarean section to consanguineous Egyptian parents. The family history is notable for an intrauterine fetal death and a stillbirth in older siblings. Her birth measurements included a weight of 2.9 kg (-1.00 SD), height of 47 cm (-1.10 SD), and an OFC of 33 cm (-1.22 SD).

She presented with delayed developmental milestones and intellectual disability. She has moderate intellectual disability and speaks only in single letters or words. Sitting was achieved at 1 year of age, and walking independently at 2 years and 3 months. At her current age, she can walk approximately 10 meters. Fine motor development is delayed; she could grasp objects and hold a pen by 1.5 years but continues to lack fine motor skills. She exhibits autistic features, including stereotypic movements such as hand flapping and excessive vocalizations. Additional concerns include poor concentration, hyperactivity, and limited social engagement.
Her ophthalmological findings include infrequent nystagmus, hypermetropia, and optic nerve dysfunction. An electroretinogram (ERG) performed at 4 months revealed moderate bilateral peripheral retinal dysfunction and poor macular function. Other medical history includes difficulty chewing food (preferring soft foods), restlessness, poor sleep, and skin photosensitivity.

At her most recent follow-up at 2.5 years, her growth parameters were low: OFC of 45 cm (-2.05 SD), height of 80 cm (-2.78 SD), and weight of 10 kg (-2.41 SD). Physical examination revealed a broad face, high forehead, sparse scalp hair, open and narrow mouth, abnormal hand creases, and fifth finger clinodactyly. Neurological examination showed mild hypotonia and brisk reflexes. A brain MRI demonstrated diffuse deep abnormal white matter signals in the bilateral peritrigonal area, a thin corpus callosum, and an EEG was unremarkable.

**Family 23**

This family presented with two affected siblings born to consanguineous Kurdish parents. There is no other relevant family history. The index case (F23:S1) is an 18-year-old male born term via NVD. His neonatal period was unremarkable with normal birth weight at 3.6 kg (+0.08 SD) and OFC at 35cm (0.42z). He presented with recurring predominantly febrile seizures at 15 months. His seizures were generalized and he experienced his last seizure at the age of 8. Since then, he has been seizure free on no antiepileptic drugs and his EEG was unremarkable. He had delayed achievement of speech/language milestones with speaking his first words at 6 years of age and simple sentences at 8 years. In terms of motor milestones, he started walking at 18 months and had good fine motor skills, he could hold a glass at 6 years of age He has moderate to severe intellectual disability and graduated from a special high school. Behavioral concerns include a bad temper and self-mutilation, with a recent history of punching a window during a fight, requiring surgical intervention for hand injuries. Additional medical history includes strabismus and unilateral cryptorchidism, corrected surgically at 2 years of age. Feeding, sleep, and vision have remained unremarkable. At his most recent follow-up, his growth parameters were as follows: height 168 cm (-1.06 SD), weight 54 kg (-1.61 SD), and OFC 55 cm (-0.07 SD). Dysmorphic features noted included a high-arched palate, pes planus, and arachnodactyly. Neurological examination revealed normal deep tendon reflexes, and a brain MRI was unremarkable.

The younger affected brother (F23:S2) is an 14-year old male born term through NVD. The mother experienced deep vein thrombosis during pregnancy. His neonatal period was unremarkable with normal birth weight (3.4 kg, -0.26 SD) and OFC (34cm, -0.83 SD). At 18 months, he presented with recurrent seizures, primarily during sleep, which were afebrile and generalized. He has been seizure free for 1.5 years on antiepileptic treatment and had an unremarkable EEG and MRI brain scan. Developmentally, speech acquisition was delayed. He started speaking his first words at 4.5 years of age and currently speaks single words and understands simple commands. Independent walking was achieved at 18 months, and fine motor skills were inadequate and has intention tremor. He has severe intellectual disability and attends to a special school. He is friendly, anxious and has obsessions and motor stereotypies and tics including hand flapping when excited. He can self-feed and has no sleep issues. Other medical history includes strabismus and gynecomastia. On his last follow-up, his growth parameters were height at 140 cm (-2.78 SD), weight at 48 kg (-0.32 SD) and OFC at 53 cm (-1.04 SD). His examination revealed dysmorphic features including high arched palate and pes planus. Neurological examination revealed normal deep tendon reflexes.

**Family 24**

The index case (F24:S1) is a 13-year-and-3-month-old female born at term via normal vaginal delivery to consanguineous first-cousin Middle Eastern parents. Her birth weight was 2.9 kg (-1.00 SD), and according to the parents, she was noted to have the umbilical cord around her neck at birth. First concerns became apparent shortly after birth with toe anomalies.

She has intellectual disability and delayed speech development. While her first words were not delayed, she has expressive language impairment with poor articulation. She achieved independent walking at 20 months, though her gait is abnormal with out-toeing and bilateral tight Achilles tendons. She exhibits learning difficulties, moderate attention deficit, social anxiety, and low self-esteem. She attends a regular school but requires special 1:1 classes and has low academic performance.

There is no history of seizures, feeding, or sleeping issues. An EEG conducted was unremarkable. Her medical history includes astigmatism, alopecia, vitamin D deficiency, and short stature attributed to growth hormone deficiency, for which she is receiving growth hormone therapy. Family history is notable for a brother with isolated lactic acidosis of unknown etiology and two siblings with short stature.

At her last follow-up at 13 years and 3 months, her growth parameters were significantly low, with a height of 135.5 cm (-3.12 SD) and weight of 29.15 kg (-2.53 SD). Physical findings included bilateral flexible pes planus with medial arch correction on tiptoeing, toe syndactyly, and hypoplasia of toes. Neurological examination was unremarkable.

**Family 25**

The index case (F25:S1) is an 8-year-and-8-month-old male, born at term via normal vaginal delivery to Egyptian consanguineous parents. His birth growth parameters were unremarkable, with a weight of -0.9 SD, height of -0.81 SD, and OFC of -0.61 SD. He initially presented with delays in motor development, cognitive skills, and articulation, and he has a moderate intellectual disability with an IQ score of 88. During assessment, he demonstrated knowledge of body parts and objects but was unable to identify colors. He has learning difficulties; while he can recognize letters at school, he is unable to read or write. Behaviorally, he struggles with poor concentration and hyperactivity.
His motor development was delayed, achieving unsupported sitting at 2 years and independent ambulation at 3 years, but he now walks well. However, his fine motor skills remain underdeveloped—he can hold and play with toys but cannot use a pencil. He began speaking single words at the age of 2, and although he currently constructs short sentences, his speech remains unclear.

He experienced apneic spells during the first 2.5 years of life, which resolved. EEGs at 2 and 4 years showed generalized epileptogenic discharges, while a follow-up EEG at 5 years and 5 months was unremarkable. Additionally, he has a squint and suffers from sleeplessness.

At his follow-up visit at 8 years and 8 months, his growth parameters included an OFC of 48 cm (-3.33 SD), weight of 17 kg (-3.14 SD), and height of 118 cm (-2.22 SD). Dysmorphic features observed included a high forehead, squint, depressed nasal bridge, smooth philtrum, thin lips, cupped large ears, an open mouth, and a thin upper lip. A neurological examination revealed normal tone and deep tendon reflexes, though he had hypotonia in early life. Brain MRI findings showed mild deep white matter signal changes, a thin corpus callosum, and mild cerebellar vermian hypoplasia.

**Family 26**

The index case (F26:S1) is a 7-year-old male born at term to non-consanguineous, healthy Caucasian parents. His birth measurements were within normal ranges, with an OFC of 34 cm (-0.83 SD), height of 47 cm (-1.18 SD) and weight of 2.81 kg (-1.20 SD). He initially presented with hypotonia, delayed speech and motor development. Speech milestones were delayed with speaking first words after the age of 2. He exhibited orofacial dyspraxia, and poor speech. Gross motor milestones were also delayed, he achieved independent sitting at 12 months, ambulation at 19 months old and he has poor coordination. Fine motor skills remain delayed, characterized by difficulties with fine motor skills, including holding a pencil, writing, brushing teeth and using a knife. He has a mild intellectual disability and is enrolled in a mainstream school. He experiences difficulty chewing hard food and snores while sleeping. There is no history of seizures or vision problems. His medical history includes asthma.

At 7 years of age, a follow-up evaluation revealed persistent difficulties with fine motor skills and mild cognitive impairment. Physical examination showed broad eyebrows, epicanthus, and normal height, weight, and OFC for age. Neurological assessment was unremarkable, with normal muscle

tone and intact deep tendon reflexes. A brain MRI conducted during this evaluation was also unremarkable.

**Family 27**

This family includes three affected siblings born to consanguineous Farsi parents who are second cousins. The eldest sibling, a 47-year-old female (F27:S1), initially presented with dependency as a primary concern. She exhibited delayed speech as well as gross and fine motor development during early childhood. She has moderate intellectual disability and did not attend school. She is diagnosed with autism spectrum disorder and is described as shy.
The individual has a history of seizures without recurrence and is currently seizure-free. Feeding and sleeping patterns are normal. Her medical history includes diabetes mellitus. On her last follow-up at 47 years of age, she was reported to have normal growth parameters, including a normal OFC, no dysmorphic features, and an unremarkable neurological examination.

The middle sibling (F27:S2), a 35-year-old female, has a history of mild to moderate intellectual disability and delayed speech and motor development. Despite these delays, her first words, independent sitting, and independent ambulation occurred within the expected timeframe during childhood. Her behavior is described as social and acceptable, and she did not attend school. Feeding and sleeping patterns are normal, and there is no significant medical history, including seizures. At her last follow-up, she was noted to have ectodermal dysplasia, dental complications, and skin lesions on the palmar surfaces. Her OFC, height, and weight were within normal ranges, and her neurological examination was unremarkable

The youngest sibling (F27:S3), a 25-year-old male, has mild to moderate intellectual disability and delayed speech. He did not attend school, and his behavior is described as social and acceptable. While his gross motor milestones were achieved on time, his fine motor skills were delayed. He has a history of seizures, for which he was treated with ASMs. His vision, feeding, and sleep patterns are normal. At his last follow-up at 25 years old, he showed no dysmorphic features, and his OFC, height, and weight were within normal ranges. His neurological examination was also unremarkable.

**Family 28**

This family presents with two affected siblings and a history of three deceased siblings. The parents are of Arab origin and are first cousins. The index case (F28:S1) is a 43-year-old male with moderate to severe intellectual disability. He attended school but did not acquire any learning. Behaviorally, he is described as agitated and anxious. He exhibited delayed speech development, speaking his first words at the age of 6 years. His gross motor development was moderately delayed, with independent sitting achieved at 2 years and walking at 4 years. Fine motor development was also moderately delayed. The individual has a history of seizures from birth, which ceased at the age of 12. His feeding is normal, but his sleep is reported to be poor. On his last follow-up at 43 years old, he was noted to have genu valgum. His OFC, weight, and height were within normal ranges, and his neurological examination was unremarkable.

The younger sibling (F28:S1) is a 30-year-old female with moderate intellectual disability and absent speech. She has delayed motor skills, achieving independent sitting at 2 years and ambulation at 4 years. She did not attend school and is described as bad-tempered. Her sleep patterns are poor, and she often wakes up during the night. She also has a history of seizures. At her last follow-up at 30 years old, she was noted to have genu valgum and flat feet. Her OFC, height, and weight were normal, and her neurological examination was unremarkable.

**Family 29**

The index case (F29:S1) is an 11-year-old male born preterm at 36 weeks with fetal distress to non-consanguineous Malay parents. His family history is significant for a sister with global developmental

delay and spasticity. At birth, his measurements included an OFC of 33.3 cm (+0.20 SD) and a weight of 2.5 kg (-0.78 SD). He first presented with intellectual disability, delayed speech, and walking. His intellectual disability is classified as moderate to severe, and he receives no formal schooling. He exhibits poor attention span, restlessness, and is unable to stand still. His speech development was significantly delayed, with his first words spoken at age 2. At 11 years old, he has only four words and is unable to form phrases. His gross motor milestones are delayed; he achieved sitting between 12 to 18 months and independent ambulation at 3 years. At his current age, he can only walk three to four steps and is unable to jump. In terms of fine motor skills, he exhibits minimal tremors and adventitious movements with some rigid posturing. He also demonstrates minimal stereotypic behavior and had bradykinesia in the early stages. His feeding is prolonged, vision is grossly normal but with a squint, and his sleep patterns are fairly normal. On his last review at 11 years old, he was minimally ambulant, stiff, and mostly dependent on others for daily activities. He was unable to read or write effectively.

Neurological examination at his last follow up at 11 years revealed truncal hypotonia with appendicular hypertonia in all four limbs, more pronounced in the lower limbs. There was spasticity mixed with rigidity, increased deep tendon reflexes in the lower limbs (4+), and clonus was present. Muscle bulk and power were normal. His posture was hypertonic. His weight was low at 16 kg (-3.98 SD). A brain MRI demonstrated a diffuse abnormal signal involving the bilateral peritrigonal white matter with areas of hypomyelination, presenting a leucomalacia-like picture, but more consistent with leukodystrophy.

**Family 30**

The index case (F30:S1) is a 4-year-old female born to consanguineous Pakistani parents with no relevant family history. Limited data is available regarding her clinical and developmental history. She was born at term via normal vaginal delivery. She presents with moderate intellectual disability, stuttering, and a history of seizures. Behaviorally, she is sociable but unable to self-feed and has not yet started schooling. There are no dysmorphic features, and her vision is normal.

**Family 31**

This family presents with two affected siblings born to double first cousin Kurdish parents. There is a family history of a parental uncle with a neurodevelopmental disorder.

The index case (F31:S1) is an 11-and-a-half-year-old male born at term, who had a rapidly resolving bilateral pneumothorax after birth. His birth weight was normal at 3.45 kg (-0.35 SD). He first presented at 2.5 years old with delayed speech. He began speaking single words at age 2 and two-word sentences at age 2.5 years, within a bilingual context. He has mild to moderate intellectual disability and attends a special needs school. His gross motor development was unremarkable, achieving unsupported sitting and ambulation at 15 months.
His past medical history includes recurrent ear infections during infancy, adenectomy, and the insertion of transtympanic aerators. At his last follow-up at 11.5 years of age, his OFC was 51.5 cm (-1.38 SD), weight was 37 kg (-0.08 SD), and height was 131.5 cm (-2.06 SD). Physical examination revealed epicanthus, splayed lower lip, brachycephaly, a small mouth, and adducted chips.

The affected sibling (F31:S2) is a 4-year-old male born at 36+6 weeks with induction due to fetal heart rate concerns. His birth measurements included an OFC of 33 cm (-1.27 SD), weight of 2.7 kg (-1.38 SD), and height of 47 cm (-1.18 SD). Concerns were first noted at birth due to finger and arm malformations and nystagmus. He has moderate intellectual disability and attends the second year of primary school with the assistance of a helper. His speech development is delayed, with the first words spoken at 3 years old. He achieved independent ambulation at 15 months. He also has nystagmus and wears glasses. On his last follow-up, physical examination revealed a wide face, brachycephaly, eversee lower lip, short philtrum, maxillary hypoplasia, adduction chips, and discreet

hallux valgus. His growth parameters at 4 years were: height 102 cm (-0.05 SD), weight 16 kg (-0.14 SD), and OFC 50 cm (-0.32 SD).

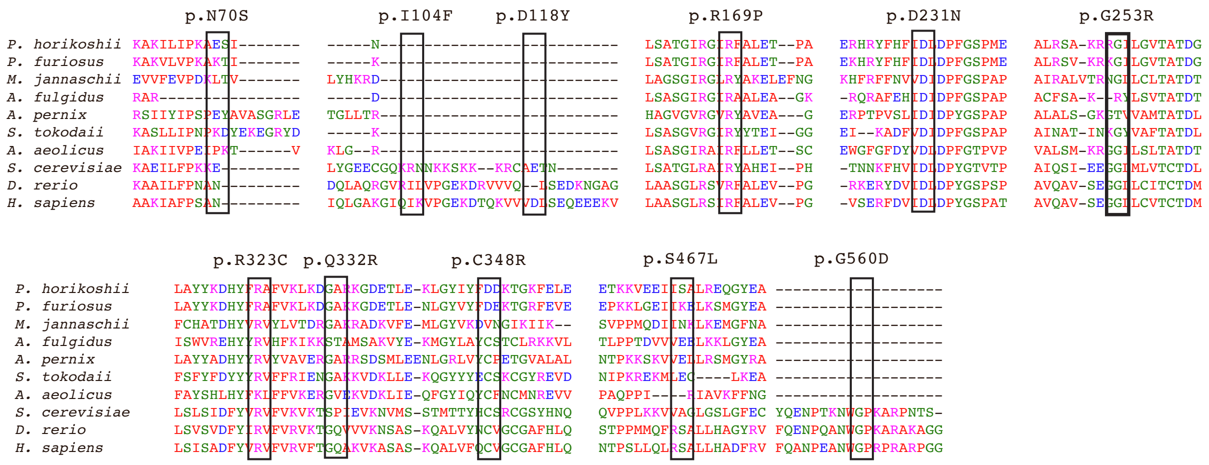


**Figure S1.** Multiple sequence alignment of TRMT1 protein orthologues shows level of conservation of the identified missense residues (indicated in dotted line boxes).

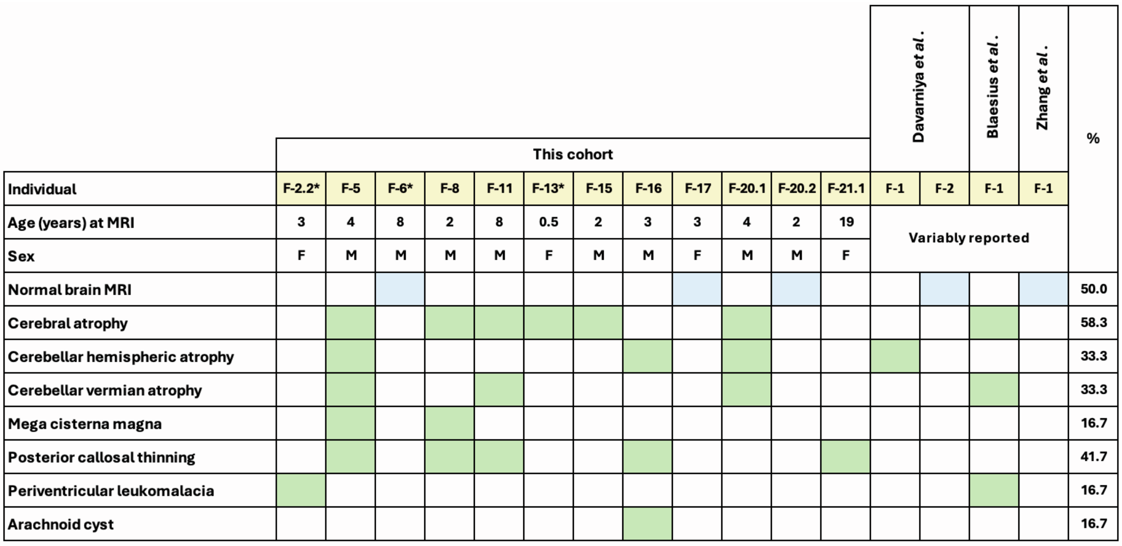


**Figure S2. Neuroimaging features of *TRMT1*-ID in our cohort (*n*=12) and previously reported cases (*n*=4) with neuroimaging available for review.** Despite significant phenotypic heterogeneity, the most prevalent neuroimaging features were cerebral and cerebellar atrophy, the latter of which could be restricted to either the vermis or cerebellar hemispheres. Global brain atrophy was present in two individuals (F-5 and F-20.1; Figure 2C). Thinning of the corpus callosum was typically limited to the isthmus and splenium, with thinning of the callosal body present in a minority and uniform thinning present in only one individual. Captions: F-1 Davarniya *et al*. corresponds to family 9000105, individual 2; F-1 Blaesius *et al*. corresponds to individual V.2. *Denotes local neuroimaging not available for central review.

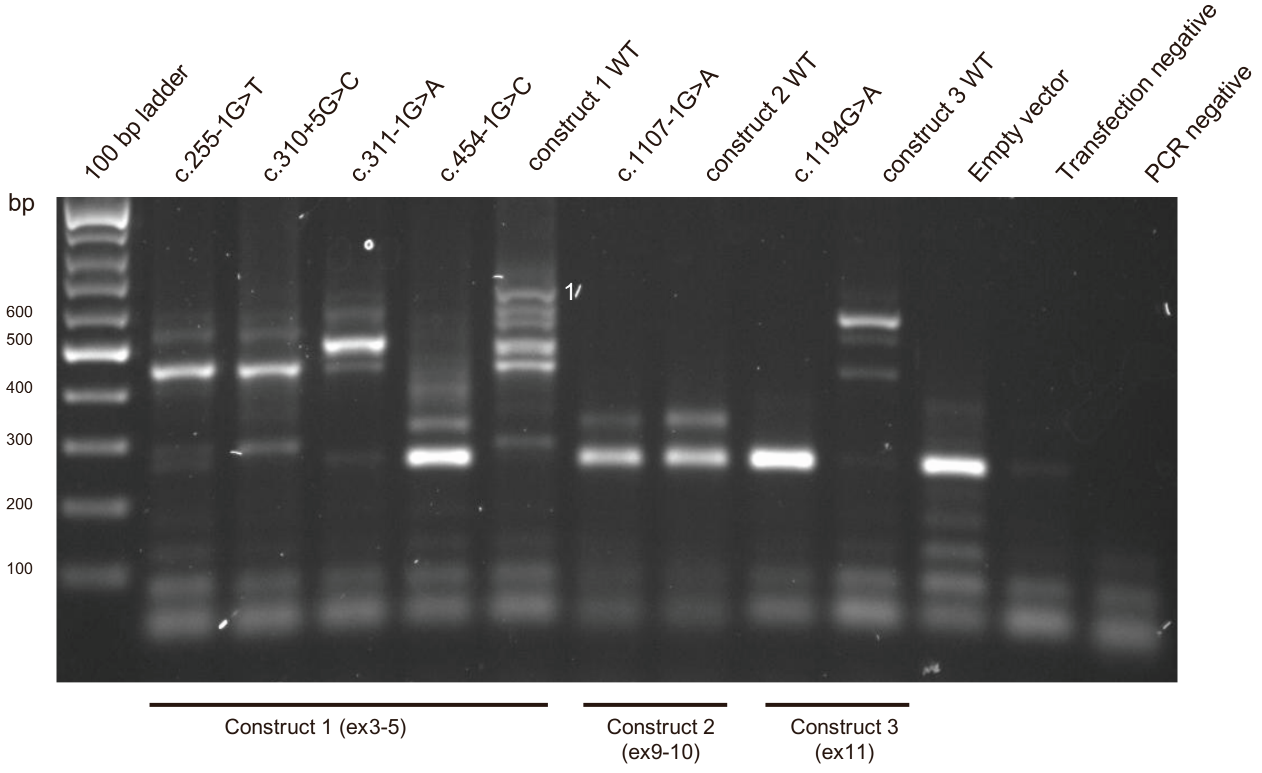


**Figure S3. Agarose gel analysis of RT-PCR reactions from *TRMT1* minigene reporter constructs.** The indicated constructs were transfected into 293T human cells and RT-PCR performed on RNA. The reactions were loaded onto a 1% agarose gel and visualized by staining.

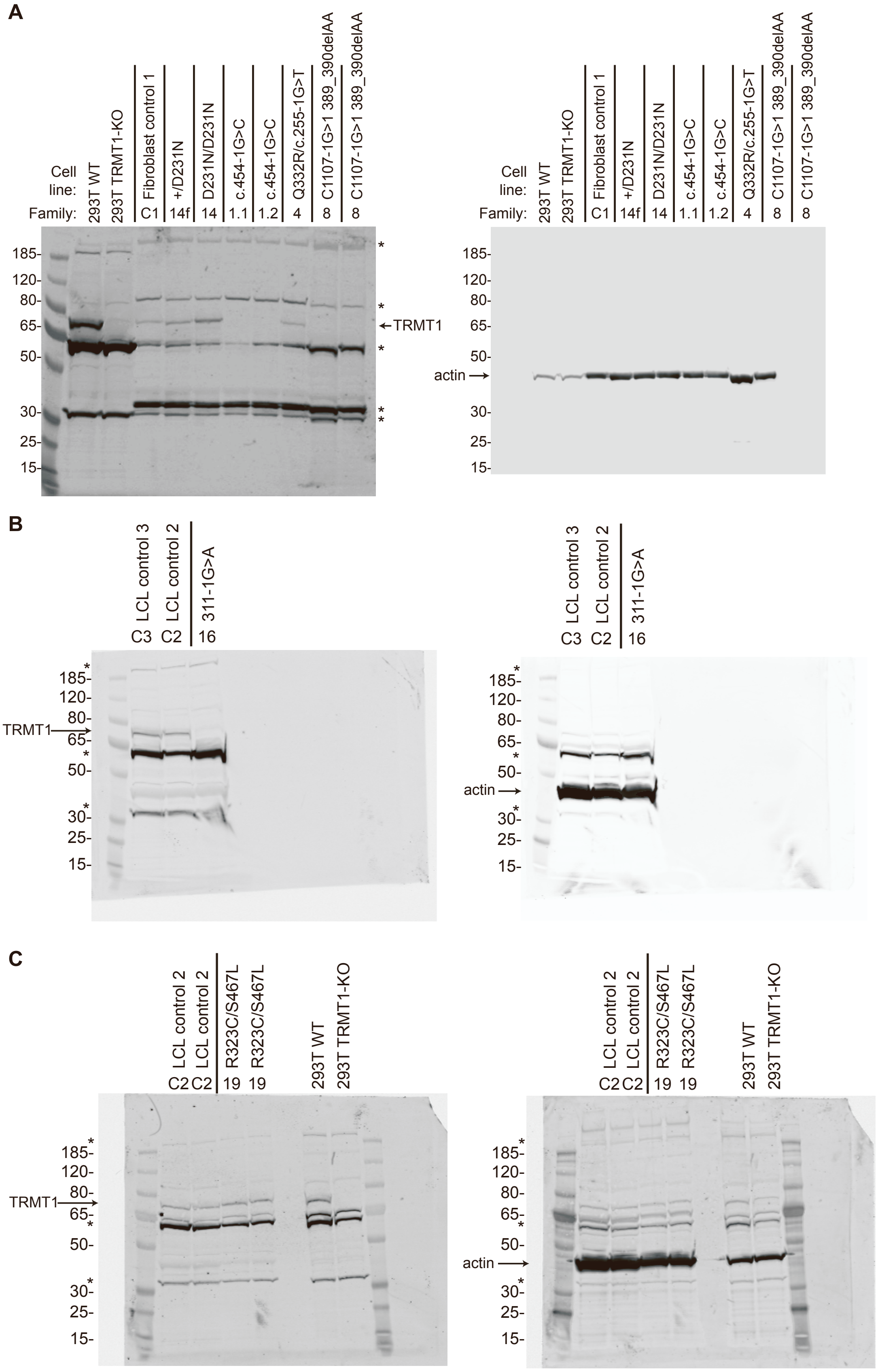


**Figure S4.** (A to C) Full scans of immunoblots shown in Figure 3. The full-length TRMT1 and actin protein bands are noted with arrows. Asterisks (*) represent non-specific bands. Molecular weight markers are in kiloDaltons (kDa).

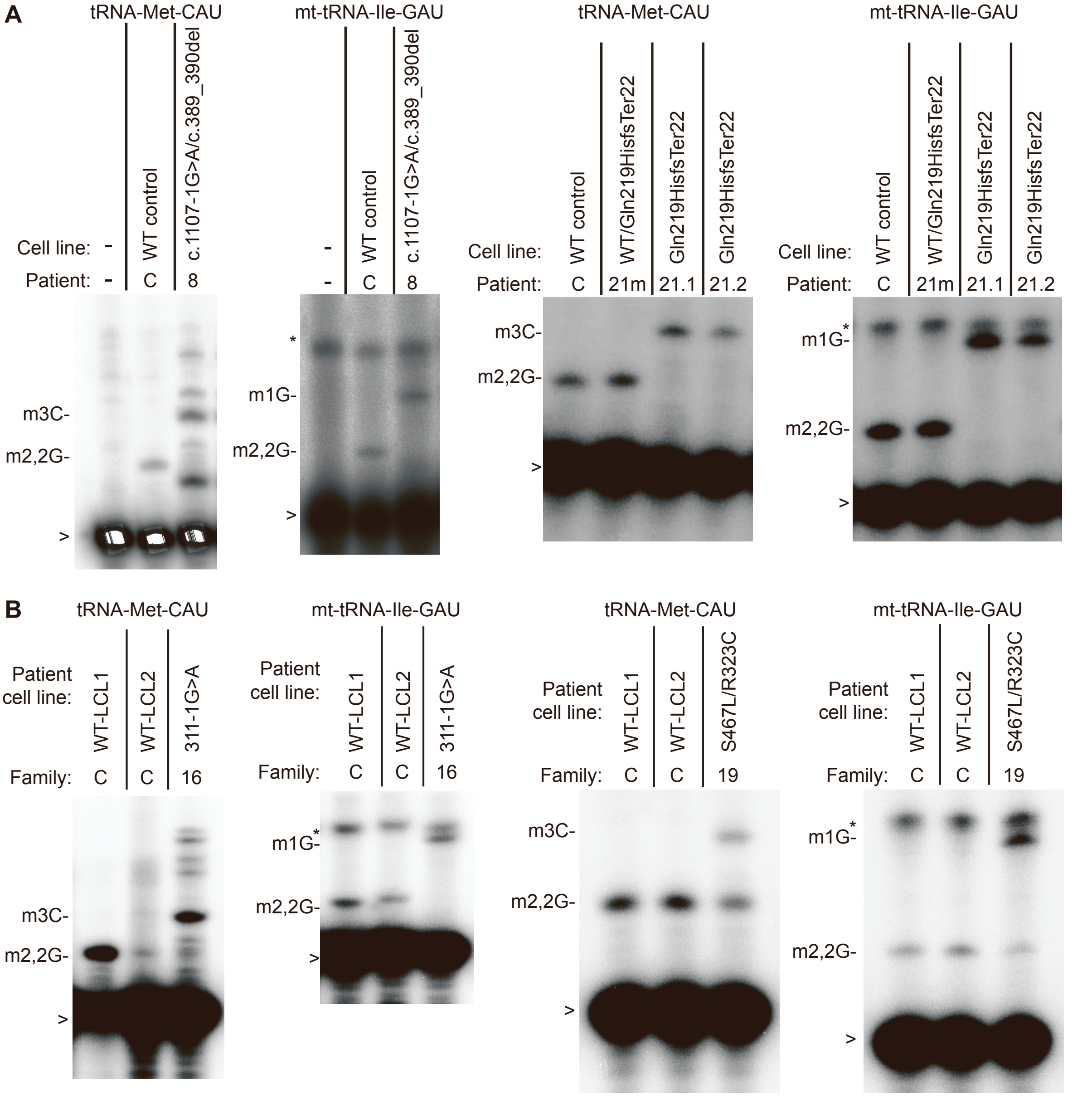


**Figure S5. Cell lines from affected individuals with biallelic *TRMT1* variants exhibit a reduction in m2,2G modifications in tRNAs.** Representative gels of primer extension assays to monitor the presence of m2,2G in tRNA-Met-CAU and mt-tRNA-Ile-GAU from: (A) fibroblast cell lines or (B) lymphoblastoid cell lines. m3C$_{20}$, 3-methylcytosine; m2,2G$_{26}$, dimethylguanosine; m1G$_{9}$, 1-methylguanosine; >, labeled oligonucleotide used for primer extension; *, background signal.

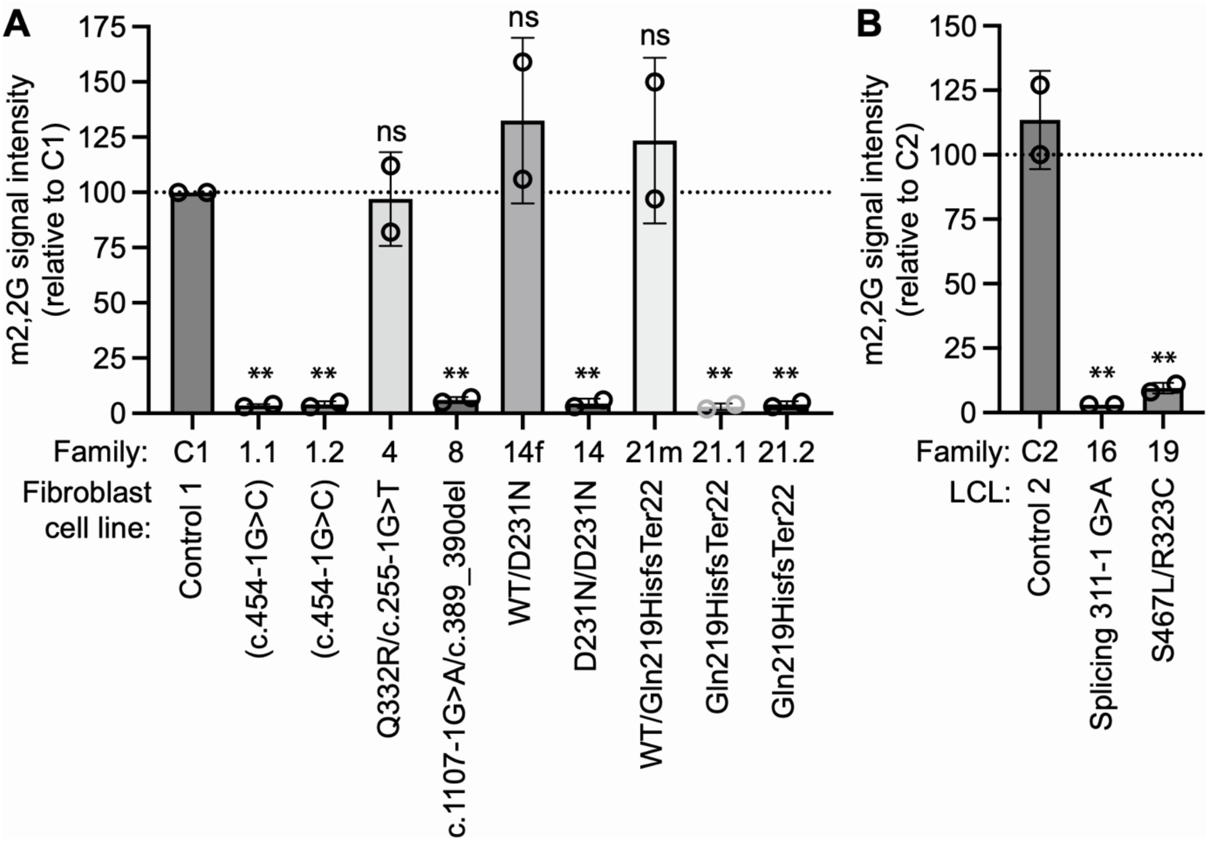


**Figure S6. Cell lines from affected individuals with biallelic *TRMT1* variants exhibit a global reduction in m2,2G modifications.** The levels of m2,2G modifications was measured by LC-MS from: (A) fibroblast cell lines and (B) lymphoblastoid cell lines (LCLs). Each cell line was tested in replicate. The m2,2G levels were normalized to A, C, G, and U. Statistical analysis was performed using one-way ANOVA. For (A), the mean of each column was compared to the Control 1 cell line. For (B), the mean of each column was compared to the Control 2 cell line. $^{**}P \leq 0.01$; ns, non-significant, $P > 0.05$.

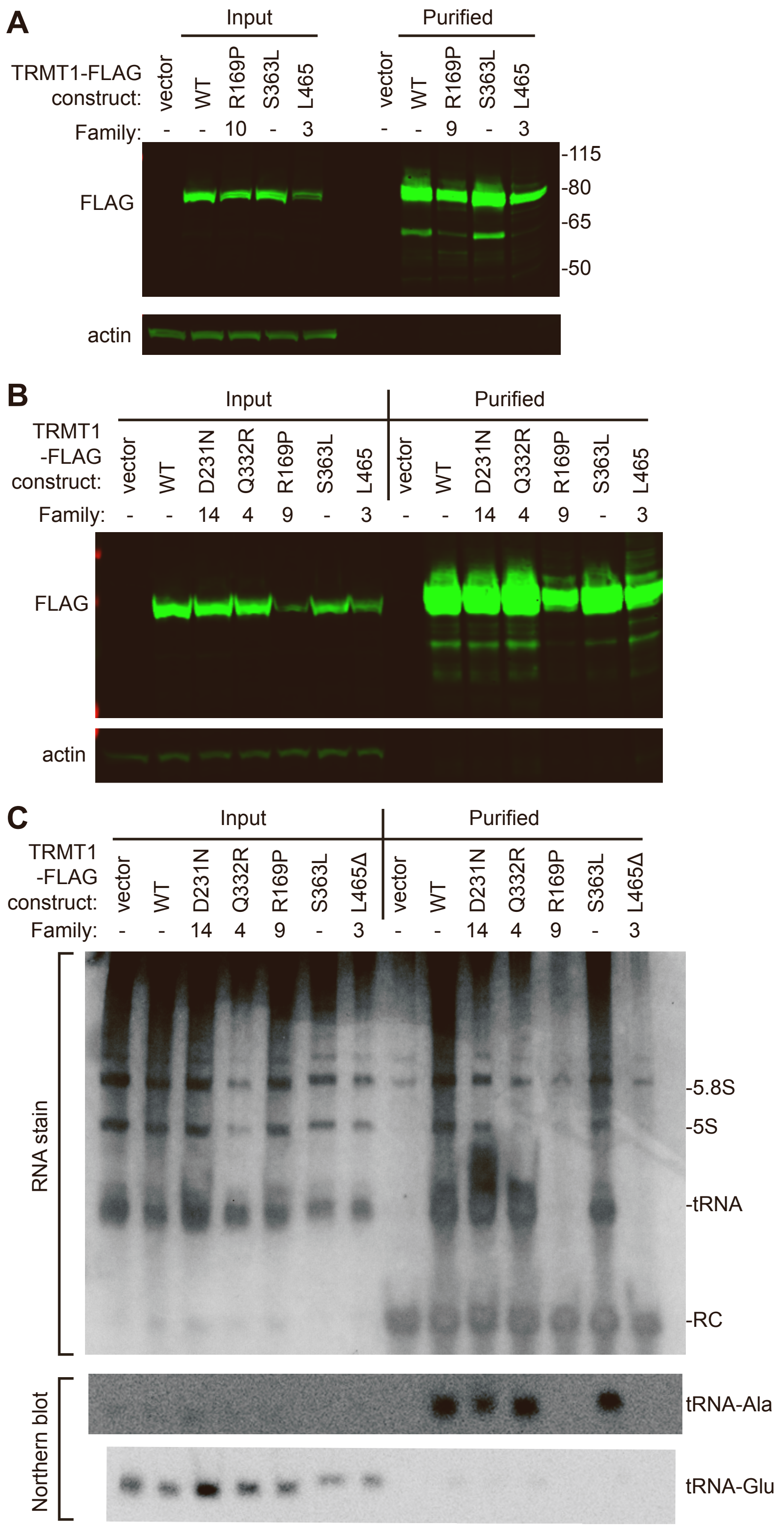


**Figure S7.** Immunoprecipitation of TRMT1-FLAG and analysis of TRMT1-tRNA interactions. (**A, B**) Immunoblot analysis of whole-cell extracts and purifications from 293T human embryonic kidney cells transfected with each of the indicated TRMT1-FLAG-tagged constructs. The immunoblot was probed with anti-FLAG or anti-actin antibodies. Molecular weight in kilodalton is denoted on the right. (**C**) Northern blot analysis of RNAs extracted from the indicated input or purified samples after denaturing PAGE. Top image shows nucleic acid stain of RNAs. The migration pattern of tRNAs, 5.8S, 5S, and recovery control (RC) is noted. Lower two panels represent Northern blot probing of the transferred gel. The L465_R466ΔinsW variant is denoted as L465Δ.

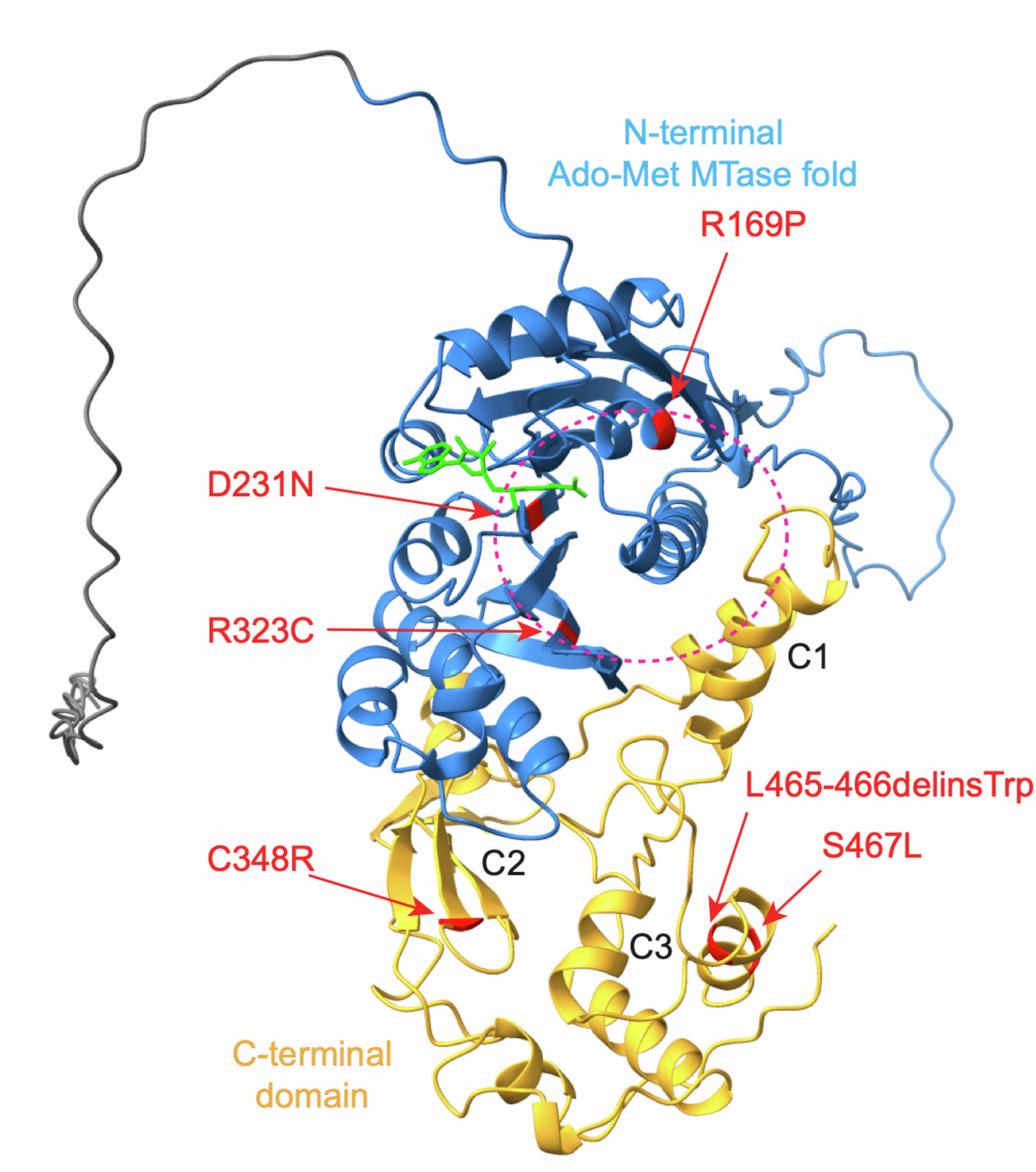


**Figure S8. Location of missense variants in the predicted structure of human TRMT1.** The methyltransferase domain along with the unstructured N-terminus (gray) are shown while the C-terminal zinc finger motif has been omitted. The human TRMT1 model was aligned with *Pyrococcus horikoshii* Trm1 bound to *S*-adenosyl-methionine (PDB: 2EJT) and domains are colored according to (20). The N-terminal Adenosyl-methionine-dependent methyltransferase domain is depicted in blue and C-terminal domain in yellow. Dashed circle represents the putative catalytic active site for binding and methylation of the G26 nucleotide in substrate tRNAs. *S*-adenosyl-methionine is denoted in green. The locations of TRMT1 variants are noted in red.

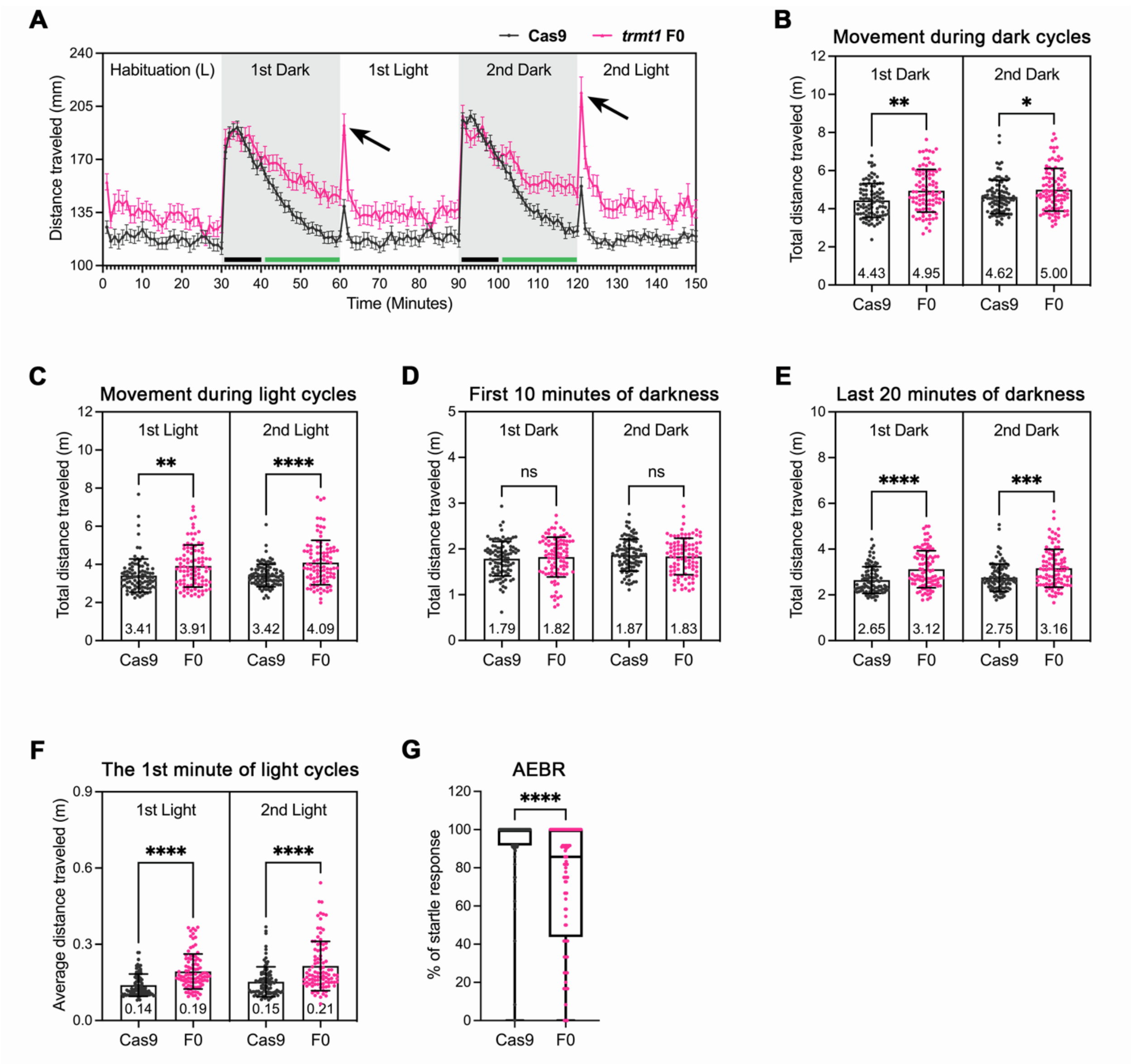


**Figure S9. Depletion of Trmt1 in zebrafish induces behavioral abnormalities.**

(**A**) Locomotor activity analysis, 96-well plates containing 96 larvae at 5 dpf were placed in a recording chamber. Larvae were habituated in the light for 30 minutes, followed by two 30-minute cycles of alternating dark and light cycles. Each point represents the average distance traveled by the animals, with n = 96 larvae each. Error bars indicate mean ± SEM. Black arrows indicate the first minute of light cycles. Black bars at the bottom indicate the first 10 minutes and green bars indicate the last 20 minutes of dark cycles. (**B**) Total distance traveled of each larva in the dark cycles. (**C**) Total distance traveled of each larva in the light cycles. (**D**) Total distance traveled of each larva in the first 10 minutes of dark cycles. (**E**) Total distance traveled of each larva in the last 20 minutes of dark cycles. (**F**) Average distance traveled calculated for each larva in the first minute of dark cycles. (**G**) A box and whisker plot showed *trmt1* F0 knockout larvae have less response to the sound stimuli. Error bars indicate mean ± SD. Each dot represents one larva. Mean value of each quantification was presented at the bottom of bar. Statistical significance was calculated by unpaired *t* test with Welch's correction: ns, non-significant, $p > 0.05$, $*p < 0.05$, $**p < 0.01$, $***p < 0.001$ and $****p < 0.0001$.

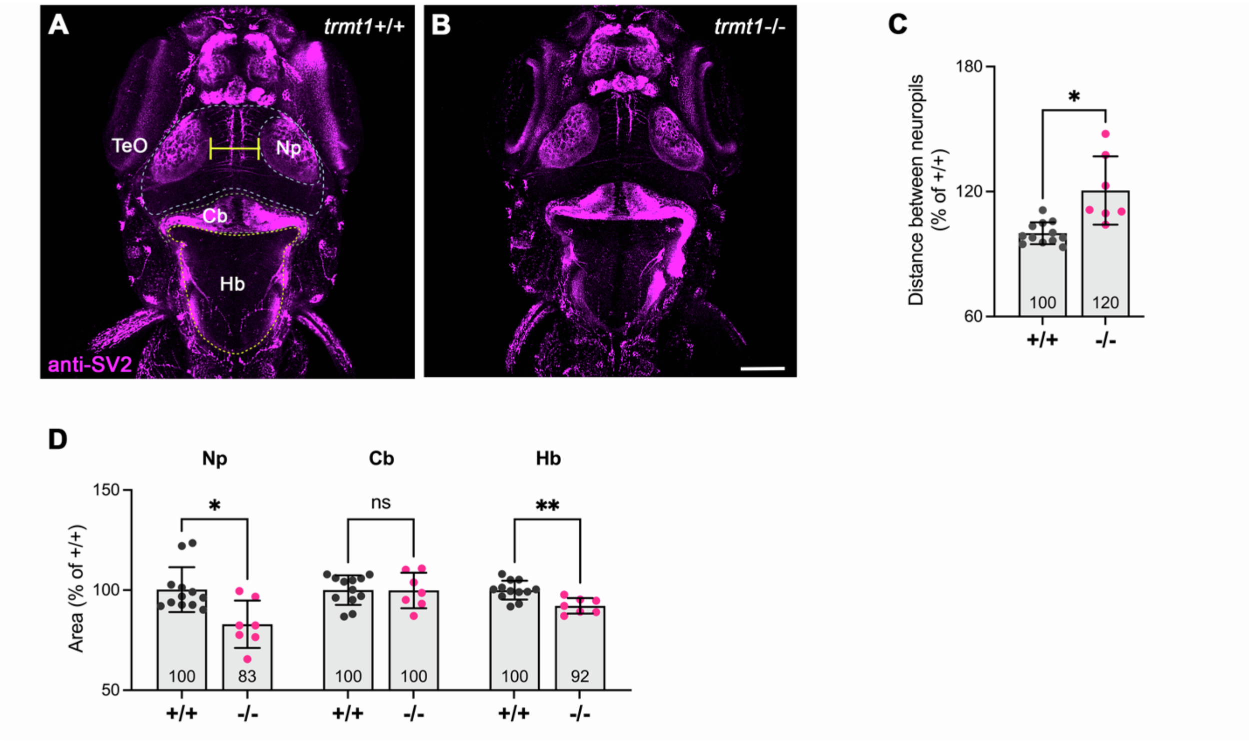


**Figure S10. Depletion of Trmt1 in zebrafish showed a reduced brain size.**
(**A, B**) Confocal images of *trmt1*+/+ (n = 12 larvae) and *trmt1*-/- (n = 7 larvae) larval brain at 5 dpf, stained with anti-SV2 (magenta). Dorsal view, anterior to the top. The brain regions are depicted by dotted line. Scale bar = 0.1 mm. (**C**) Quantification of the distance between two neuropils as indicated by the bar in A. (**D**) Quantifications of different areas as depicted by dotted lines in A. Error bars indicate mean ± SD. Values are presented as a percentage of the mean value of *trmt1*+/+ controls. Mean value of each quantification was presented at the bottom of bar. Statistical significance was calculated by unpaired *t* test with Welch's correction: ns, non-significant, $p > 0.05$, $^{*}p < 0.05$, $^{**}p < 0.01$, $^{***}p < 0.001$ and $^{****}p < 0.0001$. TeO optic tectum, Np neuropil, Cb cerebellum, Hb hindbrain.

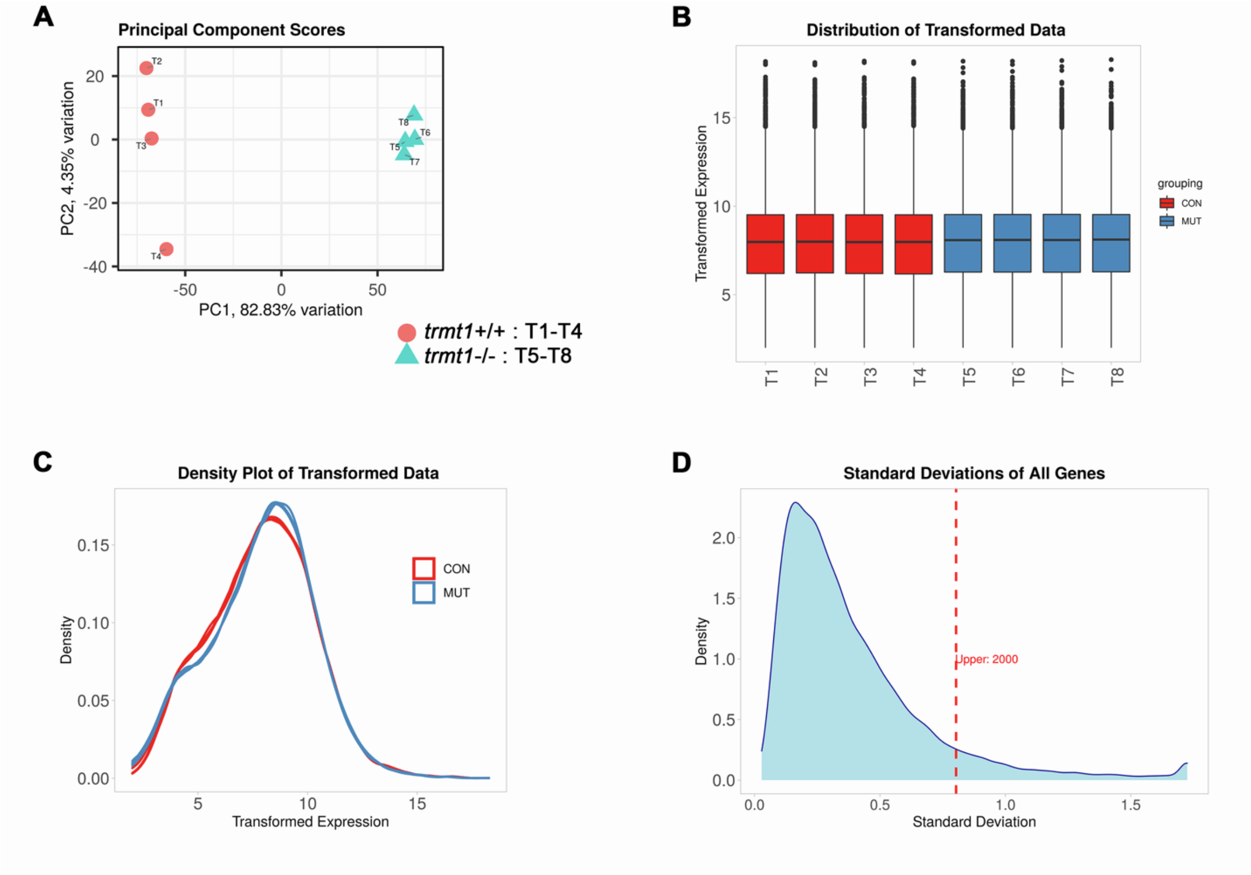


**Figure S11. Quality control (QC) plots for RNA sequencing of *trmt1*+/+ and *trmt1*-/- larvae.** (**A**) PCA plot visualizes the difference between WT controls (n = 4 biological replicates, samples named in T1 to T4) and *trmt1*-/- mutants (n = 4 biological replicates, samples named in T5 to T8). Distribution of transformed data visualized using boxplot (**B**) and density plot (**C**). (**D**) Top 2000 genes were obtained according to their standard deviation.

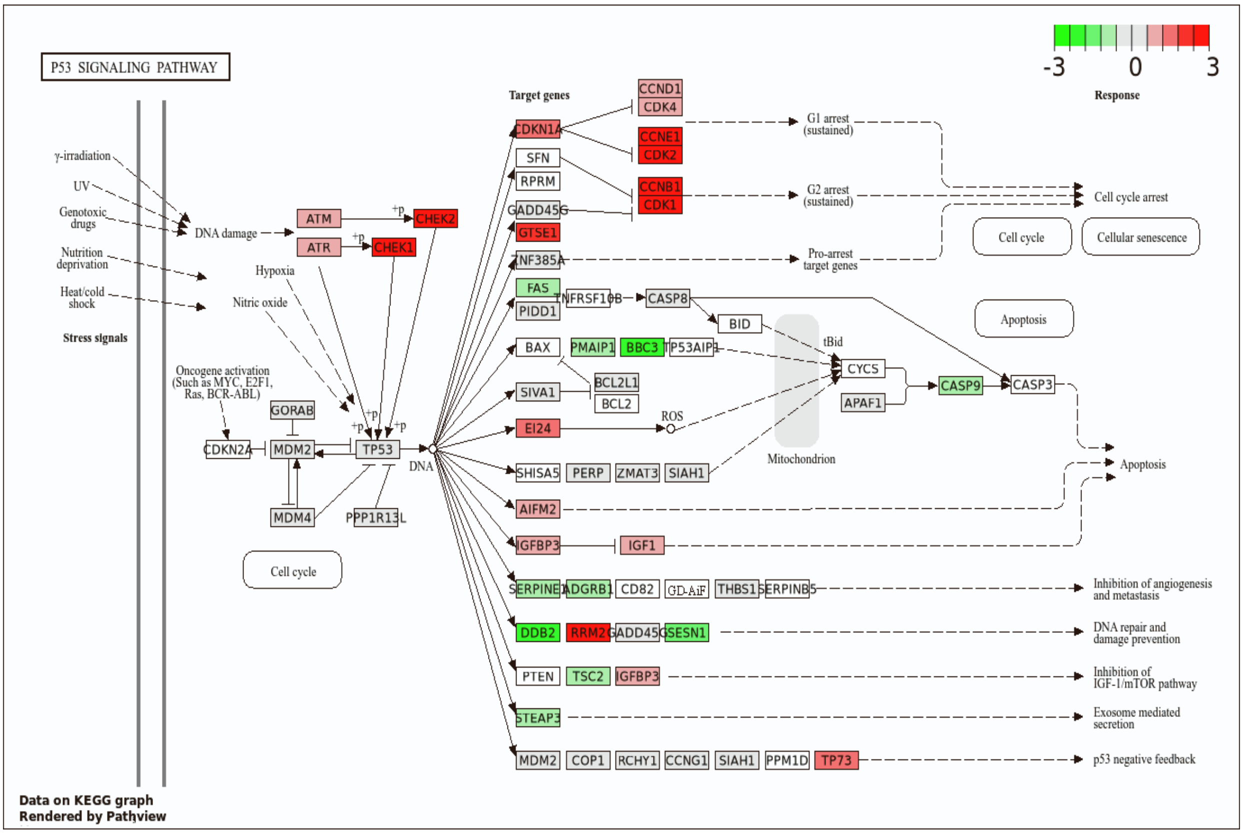


**Figure S12. KEGG pathway analysis of the p53 signaling pathway in *trmt1*-/- larvae indicates that DEGs are primarily associated with cell cycle arrest rather than cell apoptosis.**

A closed box filled with red indicates upregulated DEGs, while a box filled with green indicates downregulated DEGs. A deeper color intensity represents a stronger expression level. Gene log2-base fold change was set to ±3.

**Data S1.** Capillary fragment analyzer results for RT-PCR products of the construct 1 variants and wild-type (WT) minigenes. The numbered peaks in the upper panel of each variant analysis correspond to those in the summary table below. This analysis serves as a way to quantify the different fragment quantities expressed over the entire fragment pool.

## c.255-1G>T

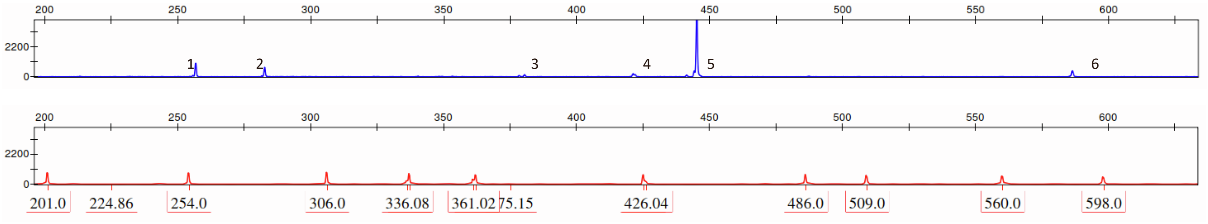


| Peak | Average size (bp) | Average height (RFU) | Average area |
|---|---|---|---|
| 1 | 256.20 | 552.50 | 4253.50 |
| 2 | 282.13 | 381.50 | 3062.50 |
| 3 | 379.33 | 124.50 | 996.00 |
| 4 | 421.54 | 188.00 | 1798.00 |
| 5 | 443.51 | 1801.00 | 16442.00 |
| 6 | 586.30 | 435.00 | 5005.00 |

## c.310+5G>C (C)

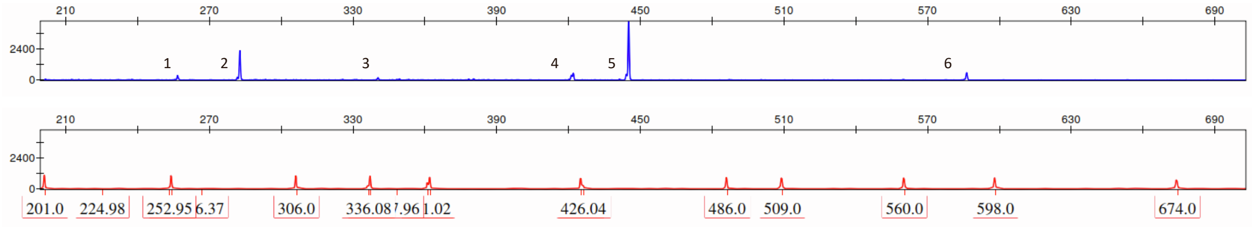


| Peak | Average size (bp) | Average height (RFU) | Average area |
|---|---|---|---|
| 1 | 256.74 | 368.00 | 2974.00 |
| 2 | 282.18 | 1262.00 | 9326.00 |
| 3 | 344.91 | 133.00 | 1069.50 |
| 4 | 421.58 | 455.00 | 4040.00 |
| 5 | 443.46 | 1786.67 | 15152.67 |
| 6 | 586.33 | 563.00 | 5664.00 |

## c.311-1G>A (T)

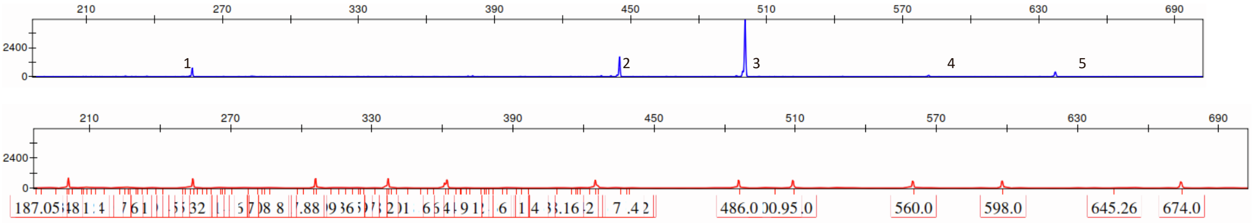


| Peak | Average size (bp) | Average height (RFU) | Average area |
|---|---|---|---|
| 1 | 256.13 | 396.00 | 2964.50 |
| 2 | 444.59 | 898.50 | 7780.00 |
| 3 | 500.01 | 2692.50 | 24511.00 |
| 4 | 581.46 | 109.00 | 1159.00 |
| 5 | 637.31 | 384.00 | 4311.00 |

## c.454-1G>C (A)

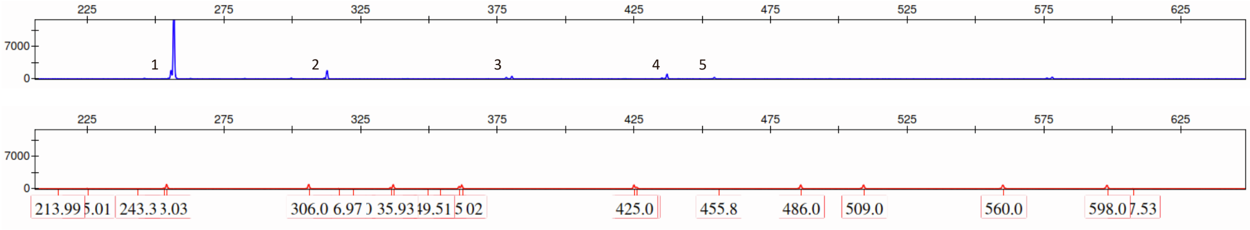


| Peak | Average size (bp) | Average height (RFU) | Average area |
|---|---|---|---|
| 1 | 255.63 | 6295.33 | 46796.33 |
| 2 | 312.20 | 999.50 | 7643.00 |
| 3 | 379.34 | 427.50 | 3241.50 |
| 4 | 436.10 | 445.00 | 3670.33 |
| 5 | 454.39 | 318.00 | 2713.00 |

WT

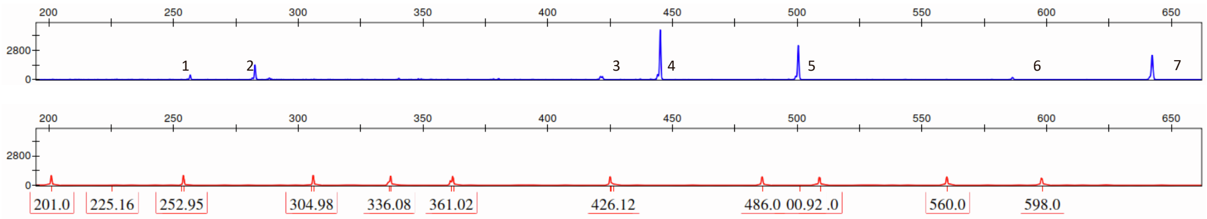


| Peak | Average size (bp) | Average height (RFU) | Average area |
|---|---|---|---|
| 1 | 256.73 | 440.00 | 3291.00 |
| 2 | 282.18 | 783.00 | 5970.00 |
| 3 | 421.59 | 297.00 | 2666.00 |
| 4 | 444.59 | 2627.50 | 22671.00 |
| 5 | 499.94 | 1795.50 | 16232.50 |
| 6 | 586.30 | 202.00 | 2203.00 |
| 7 | 642.33 | 2319.00 | 26010.00 |

**Table Legends for Excel Tables**

**Table S1.** List of the different variants in *TRMT1* including testing/methodology information, variant description, allele frequency, and in silico predictions and classifications.

**Table S2.** Clinical table of affected individuals with biallelic TRMT1 variants. Clinical description includes: Parental consanguinity, Family history, Ethnicity, Birth history, Birth weight, Birth Occipitofrontal Circumference, Birth Length, Developmental milestones, Motor changes, Intellectual disability, Behaviour, Feeding, Vision, Sleep, Abnormal movements, Seizures, other medical history, and neurological assessment.

**Table S3.** Dysmorphology assessment. Facial features of the individuals in this study. Individuals within the same family are shaded with the same color.

**Table S4.** Dysmorphology frequency. Matched HPO term and prevalence across individuals in this study.

**Table S9.** RNA-Seq analysis. Differentially expressed genes (DEGs), Normalized Expression, Heatmap cluster, Gene ontology-Biological Process (GO-BP), and Diseases pathway analysis are contained on each sheet.

**Table S5.** Splice Predictions of *TRMT1* variants. Predicted splicing changes according to the listed prediction tools. Abbreviations: AG, acceptor gain; AL, acceptor loss; DL, donor loss. [1]Acceptor loss relative to native splice acceptor site. [2]Cryptic donor gain

| Prediction Tool | c.255-1G>T | c.310+5G>C | c.311-1G>A | c.454-1G>C | c.1107-1G>A[1] | c.1194G>A[2] |
|---|---|---|---|---|---|---|
| SpliceSiteFinder-like | -100% | -100% | -100% | -100% | -100% | +77.2% |
| MaxEntScan | -100% | -100% | -100% | -100% | -100% | +35.8% |
| NNSPLICE | -100% | -98.9% | No prediction | -100% | -100% | +90.0% |
| GeneSplicer | -100% | -100% | -100% | -100% | -100% | +9.2% |
| SpliceAI 10k [≥0.2\|0.5\|0.8] | 0.79 AL (-1) | 0.92 DL (5) | 0.77 AG (-6)<br>0.90 AL (-1) | 0.50 AG (-5)<br>0.99 AL (-1) | 0.85 AG (-2)<br>0.94 AL (-1) | No change |
| AbSplice [≥0.01\|0.05\|0.2] | 0.32 | 0.31 | 0.11 | 0.37 | 0.30 | No change |

**Table S6.** Minigene assay primers. The region of interest, primer name, primer sequence, and expected product size are noted.

| Region of Interest | Primer Name | Primer Sequence 5´- 3´ | Product Size |
|---|---|---|---|
| Construct 1 Exons 3-5 | TRMT1 Ex3-5 XhoI F | aattctcgagGAATTCAATCGGGACCTGAC | 1002 bp |
| | TRMT1 Ex3-5 BamHI R | attggatccGGGCTCAAAGAGGCTAAGTC | |
| Construct 2 Exons 9-10 | TRMT1 Ex9-10 XhoI F | aattctcgagCCAGTCTAAGGGAGGAGTTGG | 416 bp |
| | TRMT1 Ex9-10 BamHI R | attggatccGATGGGCTCTGCCCACAT | |
| Construct 3 Exons 11-12 | TRMT1 Ex11-12 EcoRI F | aattGAATTCTTAGGGCCAAGTTCTCTGCA | 446 bp |
| | TRMT1 Ex11-12 NotI R | attGCGGCCGCTGGTGTGTTGCAGTGGATG | |
| pSPL3 Exons A and B | SD6 F | TCTGAGTCACCTGGACAACC | -- |
| | SA2 R | ATCTCAGTGGTATTTGTGAGC | |
| pSPL3 Exons A and B | SD6 F-FAM | FAM-TCTGAGTCACCTGGACAACC | -- |
| | SA2 R-FAM | FAM-ATCTCAGTGGTATTTGTGAGC | |
| Vector pCR2.1 | M13 F | GTAAAACGACGGCCAG | -- |
| | M13 R | CAGGAAACAGCTATGACC | |

**Table S7.** Quantitative fragment-based analysis of splice products from minigenes.

| *TRMT1* c.255-1G>T | | | | | |
|---|---|---|---|---|---|
| **Average size (bp)** | **Actual size (bp)** | **Fragment (on gel)** | **Detected on gel** | **Average area** | **Percentage** |
| 586 | 588 | - | N | 5005.00 | 15.8 |
| 444 | 445 | 5 | Y | 16442.00 | 52.1 |
| 422 | 423 | - | N | 1798.00 | 5.7 |
| 379 | 381 | - | N | 996.00 | 3.2 |
| 282 | n.a. | - | N | 3062.50 | 9.7 |
| 256 | 257 | 9 | Y | 4253.50 | 13.5 |
| | | | | | |
| *TRMT1* c.310+5G>C | | | | | |
| **Average size (bp)** | **Actual size (bp)** | **Fragment (on gel)** | **Detected on gel** | **Average area** | **Percentage** |
| 586 | 588 | - | N | 5664.00 | 14.8 |
| 443 | 445 | 5 | Y | 15152.67 | 39.6 |
| 422 | 423 | - | N | 4040.00 | 10.6 |
| 345 | n.a. | - | N | 1069.50 | 2.8 |
| 282 | n.a. | + | Y | 9326.00 | 24.4 |
| 257 | 257 | 9 | Y | 2974.00 | 7.8 |
| | | | | | |
| *TRMT1* c.311-1G>A | | | | | |
| **Average size (bp)** | **Actual size (bp)** | **Fragment (on gel)** | **Detected on gel** | **Average area** | **Percentage** |
| 637 | 644bp | 1 | Y | 4311.00 | 10.6 |
| 581 | 588 | 2 | Y | 1159.00 | 2.9 |
| 500 | 501 | 4 | Y | 24511.00 | 60.2 |
| 445 | 445 | 5 | Y | 7780.00 | 19.1 |
| 256 | 257 | 9 | Y | 2964.50 | 7.2 |
| | | | | | |
| *TRMT1* c.454-1G>C | | | | | |
| **Average size (bp)** | **Actual size (bp)** | **Fragment (on gel)** | **Detected on gel** | **Average area** | **Percentage** |
| 454 | 445 | 5 | Y | 2713,00 | 4.2 |
| 436 | 437 | 6 | Y | 3670,33 | 5.7 |
| 379 | 381 | 7 | Y | 3241,50 | 5.1 |
| 312 | 313 | - | N | 7643,00 | 12 |
| 256 | 257 | 9 | Y | 46796,33 | 73 |
| | | | | | |
| *TRMT1* WT | | | | | |
| **Average size (bp)** | **Actual size (bp)** | **Fragment (on gel)** | **Detected on gel** | **Average area** | **Percentage** |
| 642 | 644 | 1 | Y | 26010.00 | 32.9 |
| 586 | 588 | 2 | Y | 2203.00 | 2.8 |
| 500 | 501 | 4 | Y | 16232.50 | 20.5 |
| 445 | 445 | 5 | Y | 22671.00 | 28.7 |
| 422 | 423 | - | N | 2666.00 | 3.4 |
| 282 | n.a. | - | N | 5970.00 | 7.5 |
| 257 | 257 | 9 | Y | 3291.00 | 4.2 |

A plus symbol denotes a band unable to be characterized from fragment analysis

**Table S8.** Primers used for zebrafish experiments. The sgRNA target sequence for generating the F0 zebrafish knockout, sequence of primers used for genotyping, and primers used for RT-qPCR are noted.

| | |
|---|---|
| All sequences are given in 5' to 3' direction | |
| **sgRNA target sequence for generating $F_0$ knockout** | |
| trmt1_gRNA_1 | GTGTAGGATCTTCCAGTGTG |
| trmt1_gRNA_2 | GGCATGATCTTCAGTATGTG |
| trmt1_gRNA_3 | GTGTAGGATCTTCCAGTGTG |
| **Pimer sequences for genotyping** | |
| trmt1_gRNA-1_geno_Forward | GCCCGGCACTAGTTATGTTA |
| trmt1_gRNA-1_geno_Reverse | TTTGGATGCTCTTGTTCGGG |
| trmt1_gRNA-2_geno_Forward | GGAGGAAGGAGCGCTATGAT |
| trmt1_gRNA-2_geno_Reverse | TGTCTGTACAGGTGATGCAC |
| trmt1_gRNA-3_geno_Forward | ACAAGTCTCTATTGCACCGC |
| trmt1_gRNA-3_geno_Reverse | AGCAGCGCATGATATCCCAT |
| **Primers used for RT-qPCR** | |
| trmt1_set-1_Forward | TGCTCTGGAAGTTCCTGGCC |
| trmt1_set-1_Reverse | GATGACATCATAGCGCTCCTTCC |
| trmt1_set-2_Forward | GGGTGTTATGGGATATCATGCGC |
| trmt1_set-2_Reverse | CTCTTGTCCTCGAGCTCTGAGG |
| 18S_Forward | TCGCTAGTTGGCATCGTTTATG |
| 18S_Reverse | CGGAGGTTCGAAGACGATCA |
